# Linking multi-gene and morphological data in the subclass Scuticociliatia (Protista, Ciliophora) with establishment of the new family Homalogastridae fam. nov.

**DOI:** 10.1007/s42995-024-00264-8

**Published:** 2024-12-19

**Authors:** Mingjian Liu, Limin Jiang, Zhe Zhang, Fan Wei, Honggang Ma, Zigui Chen, Khaled A. S. Al-Rasheid, Hunter N. Hines, Chundi Wang

**Affiliations:** 1 Key Laboratory of Evolution and Marine Biodiversity (Ministry of Education), Institute of Evolution and Marine Biodiversity, Ocean University of China, Qingdao, 266003 China; 2 College of Marine Life Sciences, Ocean University of China, Qingdao, 266003 China; 3 Zoology Department, College of Science, King Saud University, 11451 Riyadh, Saudi Arabia; 4 Harbor Branch Oceanographic Institute, Florida Atlantic University, Fort Pierce, FL 34946 USA; 5 Laboratory of Marine Protozoan Biodiversity and Evolution, Marine College, Shandong University, Weihai, 264209 China

**Keywords:** Ciliated protists, Homalogastridae fam. nov., Phylogeny, rRNA secondary structure, Scuticociliates

## Abstract

Scuticociliatia is one of the most species-rich subclasses in the phylum Ciliophora. The evolutionary relationships among Scuticociliatia groups have long been very unclear due to the homogeneity of morphology and insufficiency of molecular data. With morphological and multi-gene-based molecular data presented here, the evolutionary phylogeny of several Scuticociliatia taxa that were hitherto especially poorly defined is analyzed and discussed. The results indicate: (1) all scuticociliates cluster into two well supported and one poorly supported group, representing three order-level taxa; (2) with the support of both morphological and molecular data, a new family Homalogastridae fam. nov. is proposed in the order Philasterida; (3) *Parauronema* is formally transferred to Uronematidae and *Potomacus* is treated as *incertae sedis* in the order Philasterida, therefore Parauronematidae is proposed to be a junior synonym of Uronematidae; (4) the genus *Madsenia* and the species *Parauronema longum* and *Pseudocyclidium longum* are treated as *incertae sedis*, while the genus *Protophyra* should be maintained in the family Ancistridae. In addition, the putative secondary structure of internal transcribed spacer 2 (ITS2) of representative taxa from the three orders of Scuticociliatia are analyzed, and consensus structures and nucleotide composition in each order are exhibited.

**Supplementary Information:**

The online version contains supplementary material available at 10.1007/s42995-024-00264-8.

## Introduction

Scuticociliates are a speciose and cosmopolitan group of ciliates that have been found in various aquatic and terrestrial habitats (Foissner et al. 1994; Lynn 2008; Wang et al. 2022a). They can exist as free-living forms, as symbionts, or as facultative parasites associated with invertebrates such as crustaceans, mollusks, and echinoderms, and with vertebrates such as many species of fishes, often leading to diseases (e.g., scuticociliatosis) and thereby economic losses in aquaculture (Gao et al. 2017; Li et al. 2024b; Lynn 2008; Magalhães Cardoso et al. 2017; Poláková et al. 2023; Song et al. 2003; Zhang and Vďačný 2024).

**Fig. 1:**
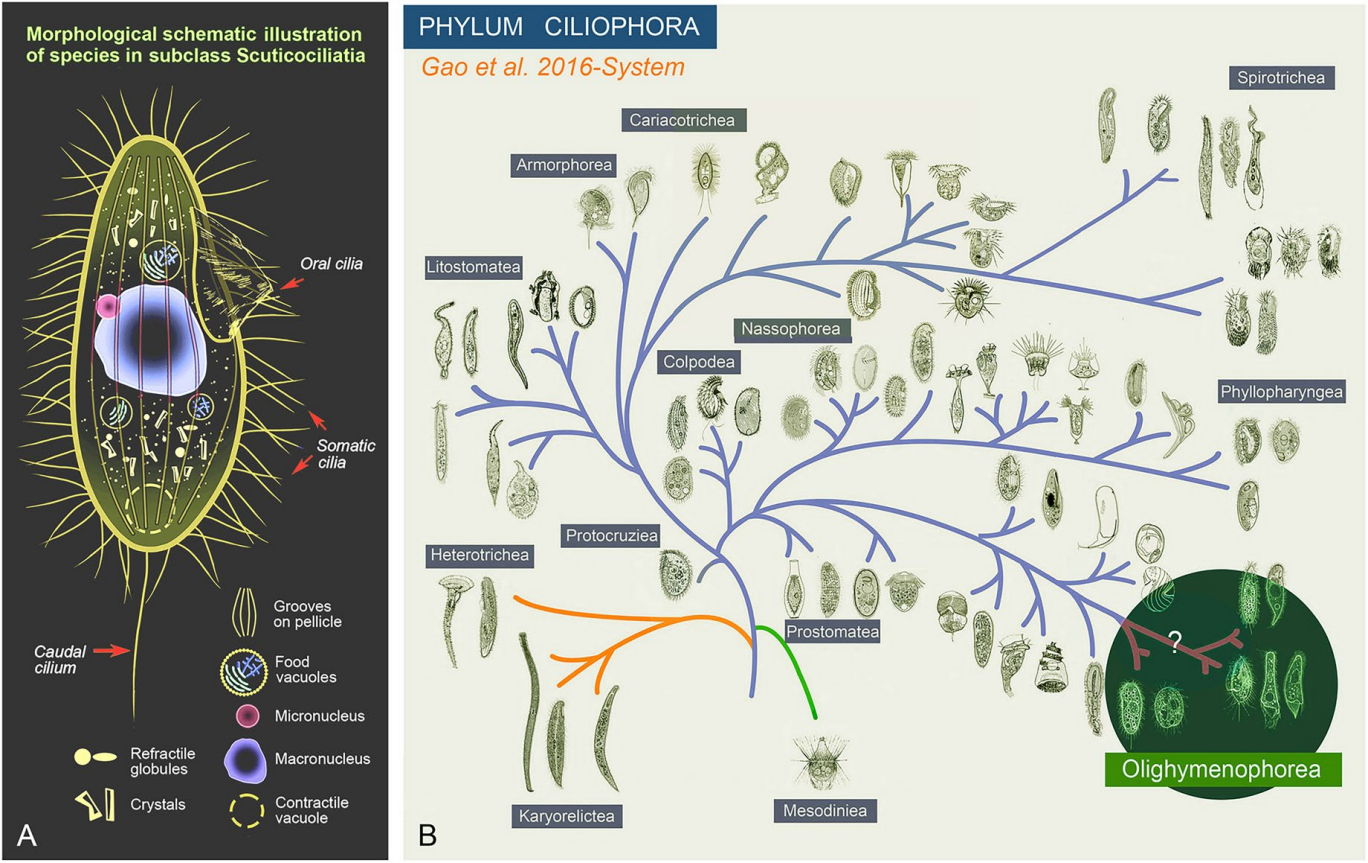
Morphological schematic illustration of a representative of the subclass Scuticociliatia (**A**) and the phylogenetic position of the class Oligohymenophorea in the phylum Ciliophora according to Gao et al. (2016) (**B**)

Scuticociliates are mainly characterized by their relatively small cell size, typically microphagous (mainly bacterivorous) feeding, and the presence of a scutica (a kinetosomal structure or organelle of scuticociliates) during stomatogenesis (Fig. 1A) (Dragesco and Dragesco-Kernéis 1986; Lynn 2008; Song et al. 2009). Scuticociliates were initially classified as the order Scuticociliatida Small, 1967 based on morphological and morphogenetic information. With further in-depth research, this taxon has been elevated to the subclass level within the class Oligohymenophorea (Fig. 1B) (Gao et al. 2016; Lynn 2008; Puytorac 1994). Currently, the subclass Scuticociliatia Small, 1967 is widely accepted to comprise three orders, namely Philasterida Small, 1967, Pleuronematida Fauré-Fremiet in Corliss, 1956, and Loxocephalida Jankowski, 1980 (Gao et al. 2016; Lynn 2008). The classification within these orders, however, has generally been established based only on morphological information, especially structures of the oral apparatus. Due to the limited diagnostic and other morphological features in vivo, and even in silver-stained cells revealing their ciliature pattern, scuticociliates are considered as one of the most systematically confused ciliate groups. Morphology-based classifications are often in conflict with molecular phylogeny, as several family-level taxa are revealed as polyphyletic, leading to uncertainty as to the true taxonomy and evolutionary relationships (Gao et al. 2012a; Lynn and Strüder-Kypke 2005; Poláková et al. 2021).

Based on extensive research conducted by the authors’ group and research teams globally over a span of 30 years, the present study reevaluated the phylogeny and systematic assignment of taxa within the subclass Scuticociliatia by integrating morphological and molecular information. Molecular phylogenetic analyses with and without environmental sequences, sequence comparison, internal transcribed spacer 2 (ITS2) secondary structure, and morphological data such as cell features, oral structure, and lifestyles were comprehensively considered for systematic reconsideration of main lineages, with emphasis on two families (Parauronematidae and Uronematidae) the systematics of which are currently confused.

## Materials and methods

### DNA extraction, PCR amplification and sequencing

The DNA of 13 morphospecies investigated in the present study was obtained from the DNA library of the Laboratory of Protozoology at the Ocean University of China. The primers 5.8S-F and 5.8S-R (Yi et al. 2009) were used for PCR amplifications of the ITS1-5.8S-ITS2 region (Supplementary Table S1). The primers 28S-F2 and 28S-R2 or 28S-F3 and 28S-R3 were used for PCR amplifications of the LSU rRNA gene (Gong et al. 2007; Moreira et al. 2007). The primers of *COI* gene were F298dT-S and R1184dT-S (Strüder-Kypke and Lynn 2010) or COI-NEW-17-F1 and COI-NEW-812-R2 (Zhang et al. 2019) (Supplementary Table S1). Q5 Hot Start High-Fidelity 2 × Master Mix (New England BioLabs) was used as DNA polymerase to minimize the possibility of PCR amplification errors (Chi et al. 2024).

The PCR program for LSU rRNA gene was as follows: one cycle of initial denaturation at 98 °C for 30 s, followed by 18 cycles of amplification (98 °C, 10 s; 69 °C–51 °C touchdown, 30 s; 72 °C, 1 min 30 s), and another 18 cycles (98 °C, 10 s; 51 °C, 30 s; 72 °C, 1 min 30 s), with a final extension of 72 °C for 5 min (Jiang et al. 2023). For the ITS1-5.8 S-ITS2 gene region, the program was basically identical to those for the LSU rRNA gene, apart from the time for the amplification cycle extension which was shortened to 45 s in both of the 18 cycles. The PCR parameters for the *COI* gene were according to Zhang et al. (2019). PCR products were purified or directly sequenced according to Lu et al. (2023) and Wu et al. (2023). Sequence fragments were assembled into contigs using Seqman 7.1.0 (DNAStar).

### Phylogenetic analyses

A total of 308 SSU rRNA gene sequences, including 245 sequences of members of the subclass Scuticociliatia, were downloaded from the GenBank database for the construction of SSU rRNA gene trees (for accession numbers, see Supplementary Table S2). There were 90 ITS1-5.8S-ITS2 gene region sequences, 64 LSU rRNA gene sequences and 116 *COI* gene sequences (including six, nine, and six newly characterized sequences, respectively) used for phylogenetic analyses (Supplementary Table S2). The sequences of each of the four genes were aligned using the MUSCLE program with the default parameters of the web server (https://www.ebi.ac.uk/Tools/msa/muscle/) (Madeira et al. 2019). The resulting alignments were refined manually by trimming the primer regions at both ends using the BioEdit 7.0.5.2 program (Hall 1999). The final alignments of the SSU rRNA gene, ITS1-5.8S-ITS2 region, LSU rRNA gene and *COI* gene used for phylogenetic analyses included 1914, 831, 2180 and 1163 sites, respectively. The alignments of each gene were concatenated using SeaView v. 4 (Gouy et al. 2010), forming three-gene (alignment order: SSU rRNA gene, ITS1-5.8S-ITS2 region, LSU rRNA gene) and four-gene (*COI* gene, SSU rRNA gene, ITS1-5.8S-ITS2 region, LSU rRNA gene) alignments. Alignment order was mainly according to Wang et al. (2022b).

For the selection of environmental or uncultured SSU rRNA gene sequences that were related to Scuticociliatia (Supplementary Table S3), each sequence of scuticociliate taxa in the present study was performed a BLAST (Basic Local Alignment Search Tool) in NCBI (National Center for Biotechnology Information). The result of BLAST was descending-reordered by sequence identity, and the environmental/uncultured sequences from the resulting top 100 sequences were downloaded. After deleting duplicate sequences or sequences that clustered within other subclasses, a total of 458 sequences, including 147 environmental/uncultured sequences and 311 Scuticociliatia-related sequences, were trimmed (alignment length: 2050 sites) and then used for phylogenetic analyses.

For each dataset, maximum likelihood (ML) analysis with 1000 bootstrap replicates was performed using RAxML-HPC2 on XSEDE 8.2.10 (Stamatakis 2014) under the GTRGAMMA model at CIPRES Science Gateway (http://www.phylo.org/sub_sections/portal) (Miller et al. 2010). Bayesian inference (BI) analysis was performed with MrBayes 3.2.6 on XSEDE 3.2.6 (Ronquist and Huelsenbeck 2003) at the CIPRES Science Gateway with the best-fit model GTR + I + G, selected under the Akaike Information Criterion using MrModeltest 2 (Nylander 2004). Markov chain Monte Carlo (MCMC) simulations were run with two sets of four chains for 10,000,000 generations at a sampling frequency of 100 and a burn-in of 10,000 trees (10%). All remaining trees were used to calculate the posterior probability (PP) using a 50% majority rule consensus. Tree topologies were visualized using SeaView v. 4 (Gouy et al. 2010) and Figtree v.1.4.4 (Rambaut 2018), and further modified using tvBOT (Xie et al. 2023). In all phylogenetic analyses, Colpodea sequences were selected as outgroups.

### ITS2 secondary structure prediction

The boundaries of the ITS2 molecule were determined by constructing the secondary structure of the ITS-5.8S rRNA molecule using the R2DT (RNA 2D Templates) web server (https://rnacentral.org/r2dt) (Sweeney et al. 2021) with *Tetrahymena thermophila* as a template and searching for the highly characteristic 5.8S-28S rRNA proximal stem. Formation of the hybridized 5.8S-28S rRNA proximal stem was forced using homology modeling and ‘mfold’ v. 3.0 (http://www.unafold.org/) (Zuker 2003). Homologies of the secondary structures of the predicted thermodynamically optimal ITS2 molecules were compared with consensus ITS2 secondary structures of the most closely related oligohymenophorean ciliates (Miao et al. 2008; Zhang and Vďačný 2024). The putative models were then prepared in VARNA v.3.93 (Darty et al. 2009). The consensus structure of the ITS2 molecule for representative species of each order of Scuticociliatia was calculated in 4SALE v.1.7.1 (Seibel et al. 2006), and the corresponding nucleotide composition logo was created with the online program WebLogo 3 (https://weblogo.threeplusone.com/) (Crooks et al. 2004).

## Results

### Characteristics of the sequences used for phylogenetic analyses

We obtained 21 novel sequences, including six of the ITS1-5.8S-ITS2 region (425–510 nucleotides; G + C contents 31.66–43.06%), nine of the LSU rRNA gene (1747–1800 nucleotides; G + C contents 44.28–47.99%) and six of the *COI* gene (695–760 nucleotides; G + C contents 25.47–27.76%) from 13 morphospecies. The accession number, length and G + C content of each of the newly characterized sequences are summarized in Table 1.

**Table 1:** Length and G + C content of the newly characterized sequences in the present study

Genes	Species/populations	Habitat	Accession number	Length (nt)	G + C content (%)
LSU rRNA gene	*Citrithrix smalli*	M	PP784539	1792	47.99
	*Homalogastra similis*	B	PP784540	1747	44.30
	*Paramesanophrys typica*	M	PP784541	1775	44.28
	*Philaster sinensis*	M	PP784536	1788	45.30
	*Uronema apomarinum*	B	PP784544	1782	44.89
	*Uronema nigricans*	F	PP784543	1789	44.61
	*Uronema orientalis* pop. 3	M	PP784538	1782	44.44
	*Uronemita filificum* pop. 2	B	PP784542	1789	45.28
	*Uronemita parabinucleata*	M	PP784537	1800	45.61
ITS1-5.8S rRNA-ITS2 region	*Citrithrix smalli*	M	PP852881	510	39.22
	*Philaster sinensis*	M	PP852879	470	34.68
	*Pleuronema parasmalli*	F	PP852884	425	43.06
	*Uronema nigricans*	F	PP852883	478	31.80
	*Uronema orientalis* pop. 3	M	PP852880	486	34.57
	*Uronemita filificum* pop. 2	B	PP852882	477	31.66
*COI* gene	*Citrithrix smalli*	M	PQ256152	721	27.46
	*Homalogastra parasetosa*	B	PQ256155	721	27.18
	*Pleuronema parasmalli*	F	PQ256156	695	25.47
	*Pleuronema puytoraci*	B	PQ256154	703	27.17
	*Pleuronema wiackowskii*	M	PQ256153	759	26.09
	*Uronema orientalis* pop. 3	M	PQ256151	760	27.76

*B* brackish water, *F* freshwater, *M* marine water

With the addition of these new sequences, in total, 245 SSU rRNA gene sequences, 55 LSU rRNA gene sequences, 73 ITS1-5.8S-ITS2 region sequences and 90 *COI* gene sequences of identified scuticociliate species/populations were used in the analyses (for GenBank accession numbers, see Supplementary Table S2). The lengths and G + C contents within the three orders of Scuticociliatia are summarized in Table 2. The G + C contents of the three regions of the rRNA gene in scuticociliates are between 31.4 and 54.02% while those of the *COI* gene are relatively low and range from 23.14 to 30.60%. The pairwise sequence identities of the SSU rRNA gene, LSU rRNA gene, ITS1-5.8S-ITS2 region and *COI* gene of subclass Scuticociliatia are 76.0–100%, 41.7–100%, 37.9–100% and 59.2–100%, respectively, with the lowest average identity in the ITS1-5.8S-ITS2 region (61.0%) (Supplementary Table S4).

**Table 2:** The length and G + C content of SSU rRNA gene, LSU rRNA gene, ITS1-5.8S-ITS2 region, and *COI* gene sequences of subclass Scuticociliatia and its three orders

	Genes	Groups	Min	Max	Mean	Median	*n*
Length	SSU rRNA gene^a^	Scuticociliatia	1519	1756	1689.0	1708	239
		Loxocephalida	1640	1733	1688.9	1692	24
		Pleuronematida	1630	1756	1695.4	1707	107
		Philasterida	1519	1718	1682.6	1710	108
	LSU rRNA gene	Scuticociliatia	884	1946	1668.9	1817	55
		Loxocephalida	1717	1846	1816.7	1838	7
		Pleuronematida	884	1946	1469.5	1790.5	16
		Philasterida	1211	1836	1736.3	1817.5	32
	ITS1-5.8S-ITS2 region	Scuticociliatia	378	563	481.4	481	73
		Loxocephalida	378	550	467.6	463	7
		Pleuronematida	425	563	482.5	462	22
		Philasterida	419	542	483.1	484	44
	*COI* gene	Scuticociliatia	634	764	726.1	749	90
		Loxocephalida	745	749	748.2	749	5
		Pleuronematida	686	764	720.8	706.5	42
		Philasterida	634	752	728.7	749	43
G + C content (%)	SSU rRNA gene^a^	Scuticociliatia	40.57	47.46	43.58	43.31	239
		Loxocephalida	42.52	47.46	44.53	44.36	24
		Pleuronematida	40.57	47.21	43.17	42.89	107
		Philasterida	42.03	45.33	43.78	43.98	108
	LSU rRNA gene	Scuticociliatia	43.92	54.02	46.09	45.35	55
		Loxocephalida	46.17	50.91	47.87	47.00	7
		Pleuronematida	43.92	54.02	46.71	46.88	16
		Philasterida	44.18	48.50	45.39	45.25	32
	ITS1-5.8S-ITS2 region	Scuticociliatia	31.40	45.18	36.10	35.53	73
		Loxocephalida	32.65	45.18	39.51	38.88	7
		Pleuronematida	33.41	43.06	37.46	37.00	22
		Philasterida	31.40	41.47	34.88	34.27	44
	*COI* gene	Scuticociliatia	23.14	30.60	26.91	26.97	90
		Loxocephalida	26.97	30.60	28.66	28.30	5
		Pleuronematida	23.14	29.24	26.10	26.63	42
		Philasterida	25.90	29.26	27.50	27.31	43

*Max* maximum, *Mean* arithmetic mean, *Min* minimum, *n* number of specimens observed

^a^Six SSU rRNA gene sequences of Scuticociliatia that are significantly shortened were deleted from the 245 Scuticociliatia sequences when calculating the length and G + C content

**Fig. 2:**
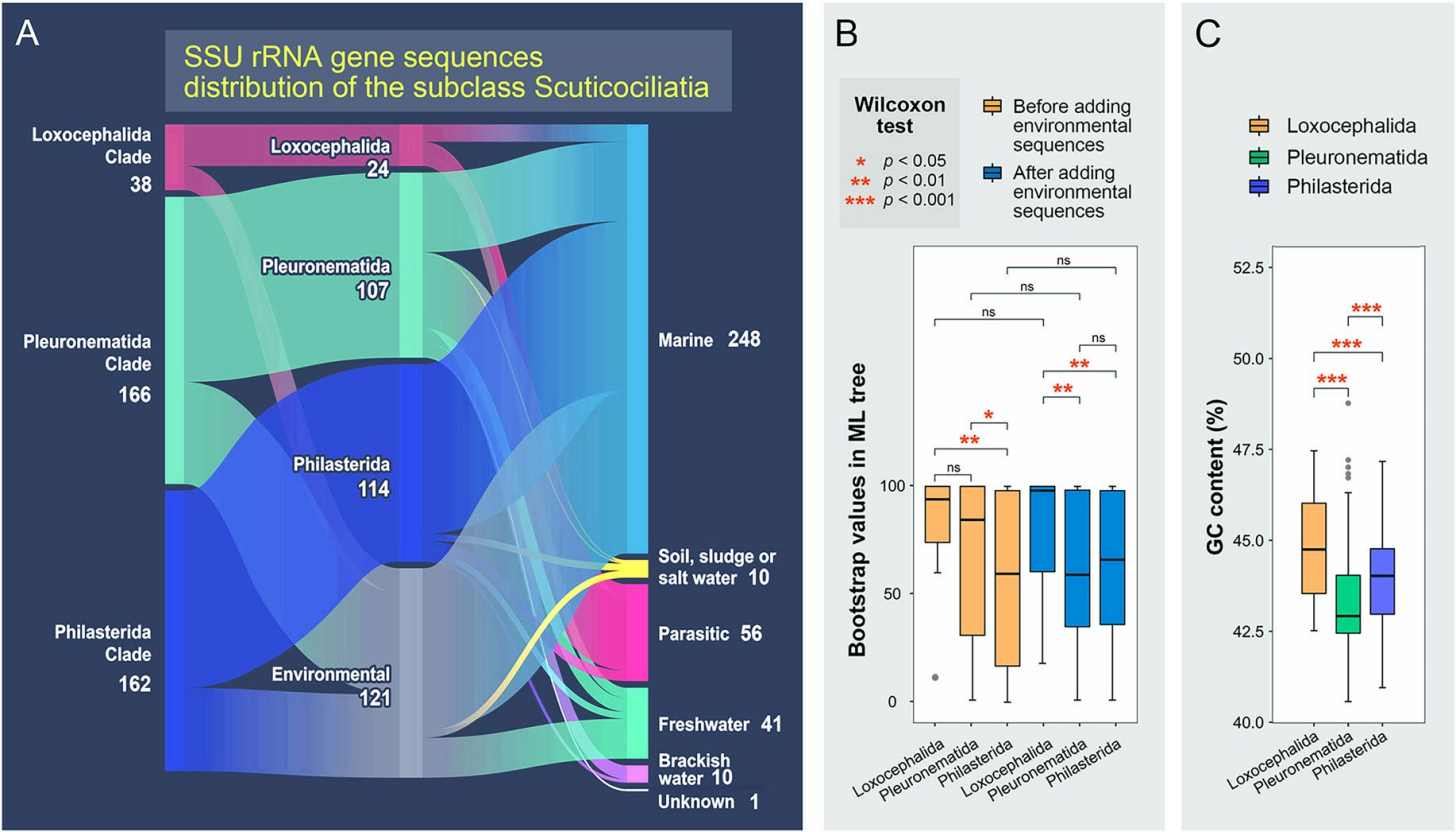
Source distribution of SSU rRNA gene sequences of three Scuticociliatia clades (**A**), numbers indicate the number of sequences; comparison of maximum likelihood (ML) tree bootstrap values before and after adding environmental sequences (**B**); and comparison of G + C content percentage of SSU rRNA gene sequences from three Scuticociliatia orders (**C**). Wilcoxon test was used for comparison. *p < 0.05; **p < 0.01; ***p < 0.001; *ns* not significant

In the GenBank database, along with sequences from the identified scuticociliates, there are numerous environmental sequences. We included these in the matrix of the aforementioned SSU rRNA gene sequences and classified them using a maximum likelihood tree. In total, 121 of the environmental sequences cluster with the orders Loxocephalida, Pleuronematida and Philasterida (Fig. 2A), and most of these environmental sequences (over 100) are from microbial community studies of specific habitats. These environmental sequences, along with the identified sequences, are from scuticociliates living in different habitats/environments, such as marine (248), parasitic in various hosts (56), freshwater (41), soil, sludge or salt water (10), and brackish water (10).

The addition of environmental sequences increased the mean supporting values in phylogenetic analyses of Loxocephalida and Philasterida, while those of Pleuronematida are decreased although the difference was not statistically significant (Wilcoxon test, *p* > 0.05) (Fig. 2B). In terms of G + C content, the Wilcoxon test showed that there are significant differences among the Loxocephalida, Pleuronematida, and Philasterida clades with environmental sequences included (*p* < 0.001). The Loxocephalida clade had the highest average percentage of G + C content, followed by Philasterida and then Pleuronematida (Fig. 2C).

### General phylogeny of the subclass Scuticociliatia based on SSU rRNA gene sequence data

**Fig. 3:**
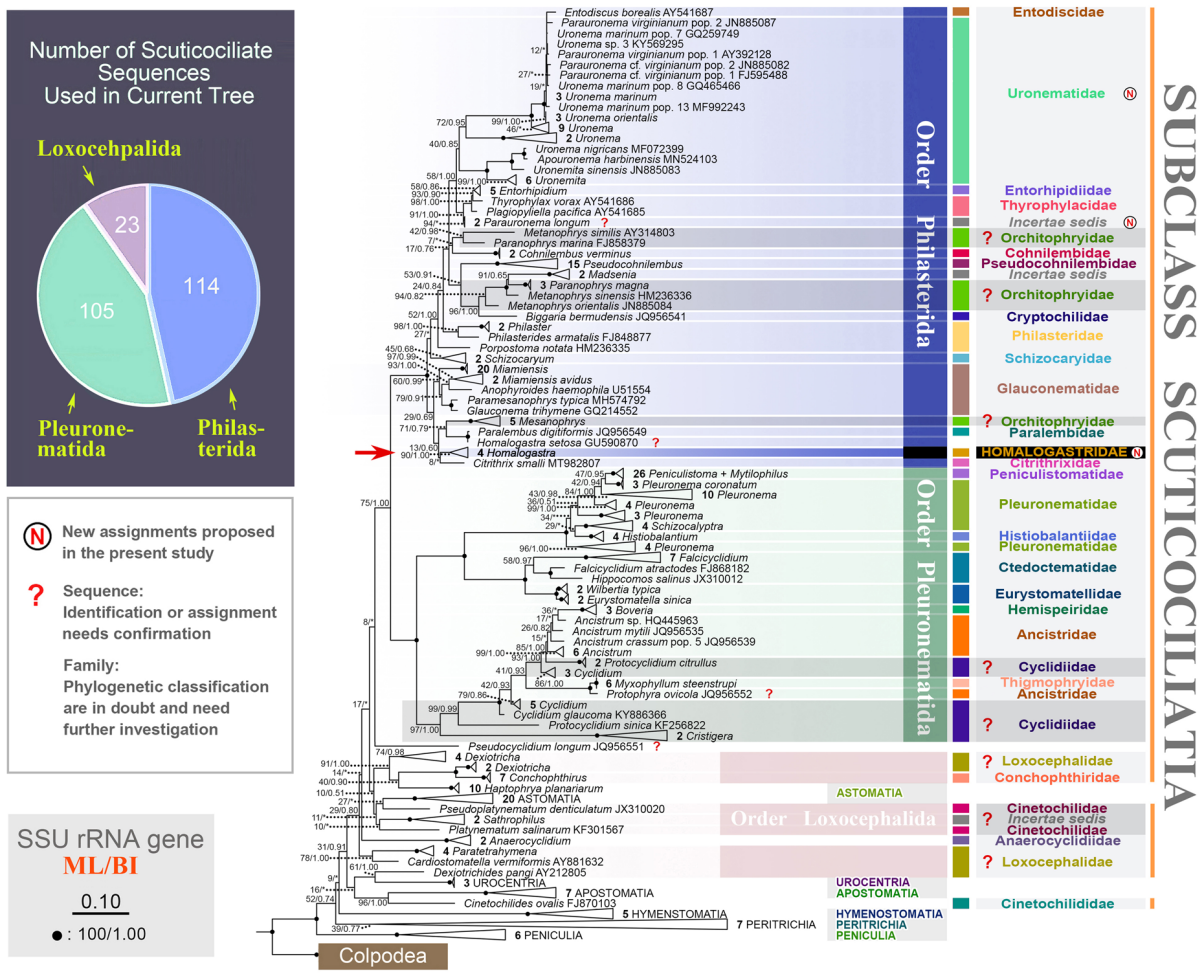
Maximum likelihood (ML) tree based on SSU rRNA gene sequence data, showing the phylogenetic positions of the taxa of subclass Scuticociliatia. Numbers at nodes denote ML bootstrap values/Bayesian inference (BI) posterior probabilities. Asterisks (*) indicate a mismatch in topologies between ML and BI analyses. Fully supported (100%/1.00) nodes are marked with solid circles. The scale bar corresponds to 10 substitutions per 100 nucleotide positions. All branches are drawn to scale. The systematic classification mainly follows Gao et al. (2016). New assignments proposed in the present study are marked with the letter “N” in the circles. Sequences with question marks indicate the identity or taxonomic assignment of these sequences needs to be confirmed. Family names with question marks indicate families that are polyphyletic and need further investigation in future studies. The number of sequences from each of the three orders of Scuticociliatia is provided in the pie chart

The topologies of the ML and BI trees are generally concordant, thus only the ML tree, with support values generated from both analyses, is presented. In SSU rRNA gene tree (Fig. 3), the order Loxocephalida is separated into several poorly supported clades. Among the three families of Loxocephalida represented in this tree, only Conchophthiridae forms a monophyletic group with full support (100% ML/1.00 BI), while Loxocephalidae and Cinetochilidae are polyphyletic (Fig. 3).

The order Pleuronematida is polyphyletic because the sequence of *Pseudocyclidium longum* is located outside the Pleuronematida + Philasterida clade, albeit with low support (8%), in the ML tree (Fig. 3). In the BI tree, *Pseudocyclidium longum* clusters with two *Sathrophilus* sequences to form a poorly supported clade (0.57 BI) that nests within the order Loxocephalida (data not shown). Except for *Pseudocyclidium longum*, all the Pleuronematida sequences cluster together (100% ML/1.00 BI) to form a group that is divided into two major clades. The first clade is fully supported and comprises five families, i.e., Eurystomatellidae, Ctedoctematidae, Pleuronematidae, Histiobalantiidae, and Peniculistomatidae, all of which are monophyletic apart from Pleuronematidae which is polyphyletic since four *Histiobalantium* sequences cluster with *Schizocalyptra*. The second clade consists of four families, i.e., Cyclidiidae, Ancistridae, Thigmophryidae and Hemispeiridae, the first two of which are polyphyletic (97% ML/1.00 BI).

The order Philasterida, which includes 14 families, forms a monophyletic group (100% ML/1.00 BI) that is sister to the main clade of Pleuronematida (Fig. 3). Within the Philasterida clade, there are two main groups, the Entodiscidae-Uronematidae-Orchitophryidae-Pseudocohnilembidae-Philasteridae-Schizocaryidae group and the Glauconematidae-*Mesanophrys*-Paralembidae-Homalogastridae-Citrithrixidae group, though the support values are poor (27% ML and 29% ML, respectively). In Philasterida, four families, i.e., Uronematidae, Orchitophryidae, Philasteridae, and Thyrophylacidae, are polyphyletic. Entodiscidae, Entorhipidiidae and Thyrophylacidae are located in the Uronematidae clade and four *Homalogastra* sequences group with Citrithrixidae rather than with other uronematids. In particular, these four *Homalogastra* sequences can be clearly separated from others within the *Mesanophrys*-*Paralembus*-*Citrithrix* group by having 60–91 unmatched nucleotides (75 on average) and 93.8–95.9% sequence identity (94.9% on average). Sequences of Orchitophryidae are widely distributed in the Philasterida clade. In Philasteridae, *Philaster* and *Philasterides* group together, while *Porpostoma* (represented by *P. notata*) and *Madsenia* are separated from them. *Thyrophylax vorax* clusters with Entorhipidiidae and then with *Plagiopyliella pacifica*, making Thyrophylacidae polyphyletic (Fig. 3).

### Phylogeny of the subclass Scuticociliatia based on LSU rRNA and* COI* gene sequence data

**Fig. 4:**
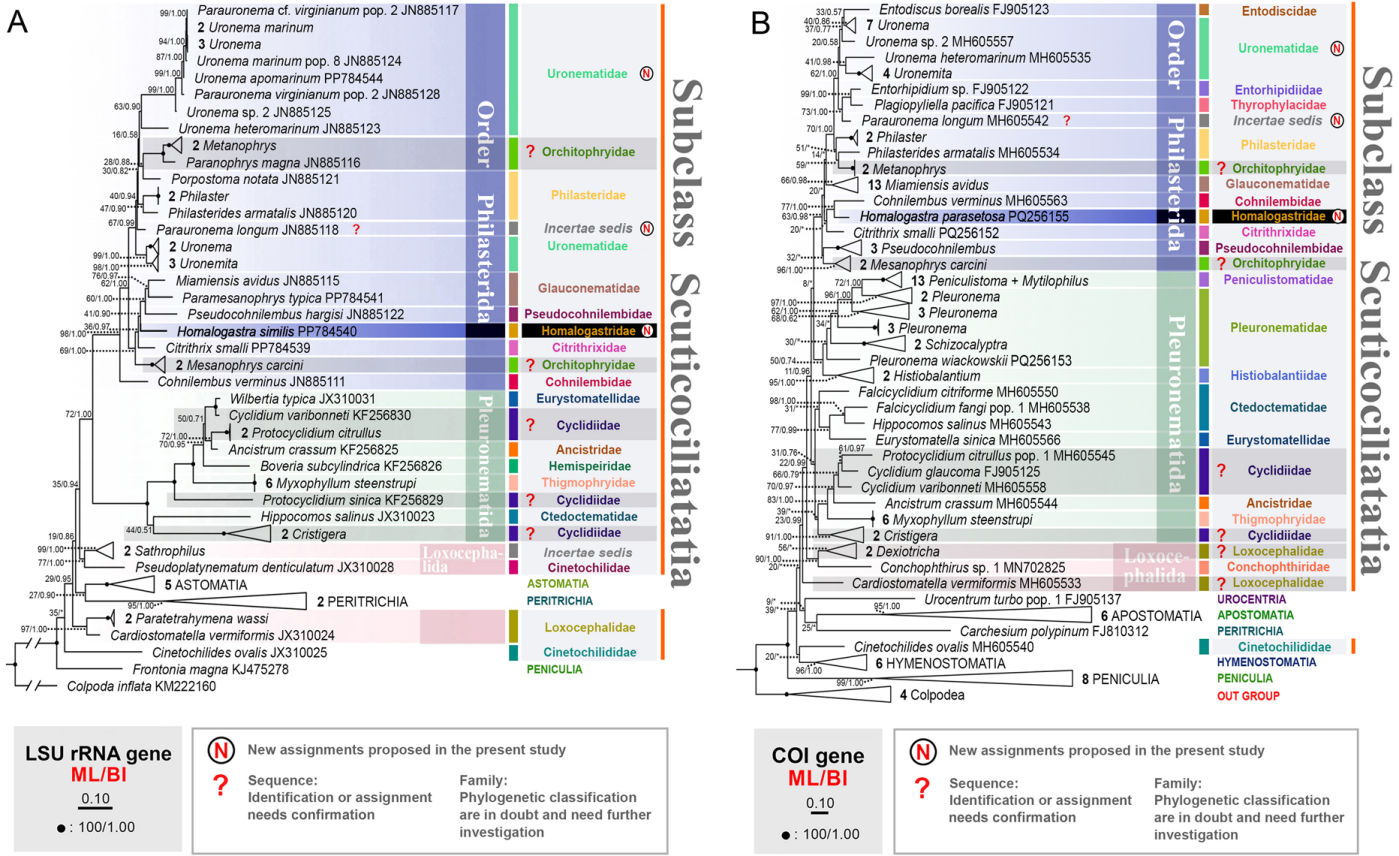
Maximum likelihood (ML) tree based on LSU rRNA gene (**A**) and *COI* gene sequence data (**B**). Numbers at nodes denote ML bootstrap values/Bayesian inference (BI) posterior probabilities. Asterisks (*) indicate a mismatch in topologies between ML and BI analyses. Fully supported (100%/1.00) nodes are marked with solid circles. The scale bar corresponds to 10 substitutions per 100 nucleotide positions. All branches are drawn to scale except for the basal branch in the LSU rRNA gene tree. The systematic classification mainly follows Gao et al. (2016). New taxonomic assignments proposed in the present study are marked with letter “N” in the circles. Sequences with question marks indicate the identity or taxonomic assignment of these sequences should be confirmed, while family names with question marks indicate families that are polyphyletic and need further investigation in future studies

The topology of the tree constructed with the LSU rRNA gene is similar to that of the SSU rRNA tree in that the Pleuronematida clade is sister to Philasterida, and Loxocephalida branches separately (Figs. 3, 4A). In the *COI* gene tree, however, Histiobalantiidae, Pleuronematidae, Peniculistomatidae and Philasterida form a large assemblage that is sister to the other members of Pleuronematida, although the support values are low (Fig. 4B). In addition, the pleurostomatid families Eurystomatellidae and Ctedoctematidae group with Cyclidiidae in the LSU rRNA tree, whereas they are widely separated from Cyclidiidae in the SSU rRNA tree (Fig. 4A).

The positions of the families in the Philasterida branch differ from those in the SSU rRNA tree. In the LSU rRNA tree, the main differences are as follows: (1) Cohnilembidae occupies the basal position of Philasterida; (2) *Pseudocohnilembus* clusters within the Citrithrixidae-Glauconematidae group; (3) *Parauronema longum*, Philasteridae, and Orchitophryidae (represented by *Metanophrys* and *Paranophrys*) nest within the *Uronemita* + *Uronema* + *Parauronema* clade (Fig. 4A), whereas in the SSU rRNA tree, they are placed outside of this clade (Fig. 3). *Homalogastra similis* is located in the group that includes *Citrithrix*, *Mesanophrys* and other taxa, which corresponds to the topology of the SSU rRNA tree. However, the SSU rRNA sequence of *H*. *similis* differs significantly from other sequences within this group, with 113–147 (132 on average) unmatched nucleotides and a sequence identity of 87.4–90.3% (88.7% on average). Furthermore, the LSU rRNA sequence of *H*. *similis* differs from other Philasterida sequences by 137–191 nucleotides (176 on average), with a sequence identity of 83.7–88.3% (85.0% on average).

The positions of Cohnilembidae and Pseudocohnilembidae in the *COI* tree also differ from those in the SSU rRNA tree, the former clustering with *Homalogastra*, while the latter clusters with Orchitophryidae (represented by *Mesanophrys*) (Fig. 4B). The only representative of *Homalogastra*, *H. parasetosa*, clusters with *Cohnilembus verminus* and *Citrithrix smalli* but can be distinguished from them by 112 and 106 nucleotides, respectively (sequence identity 80.9% and 81.9%, respectively). In addition, *H. parasetosa* can be clearly separated from other Philasterida sequences in the *COI* tree by 101–148 nucleotides (114 on average) with a sequence identity of 74.8–82.8% (80.6% on average).

### Systematic reconstruction of the subclass Scuticociliatia based on concatenated data

**Fig. 5:**
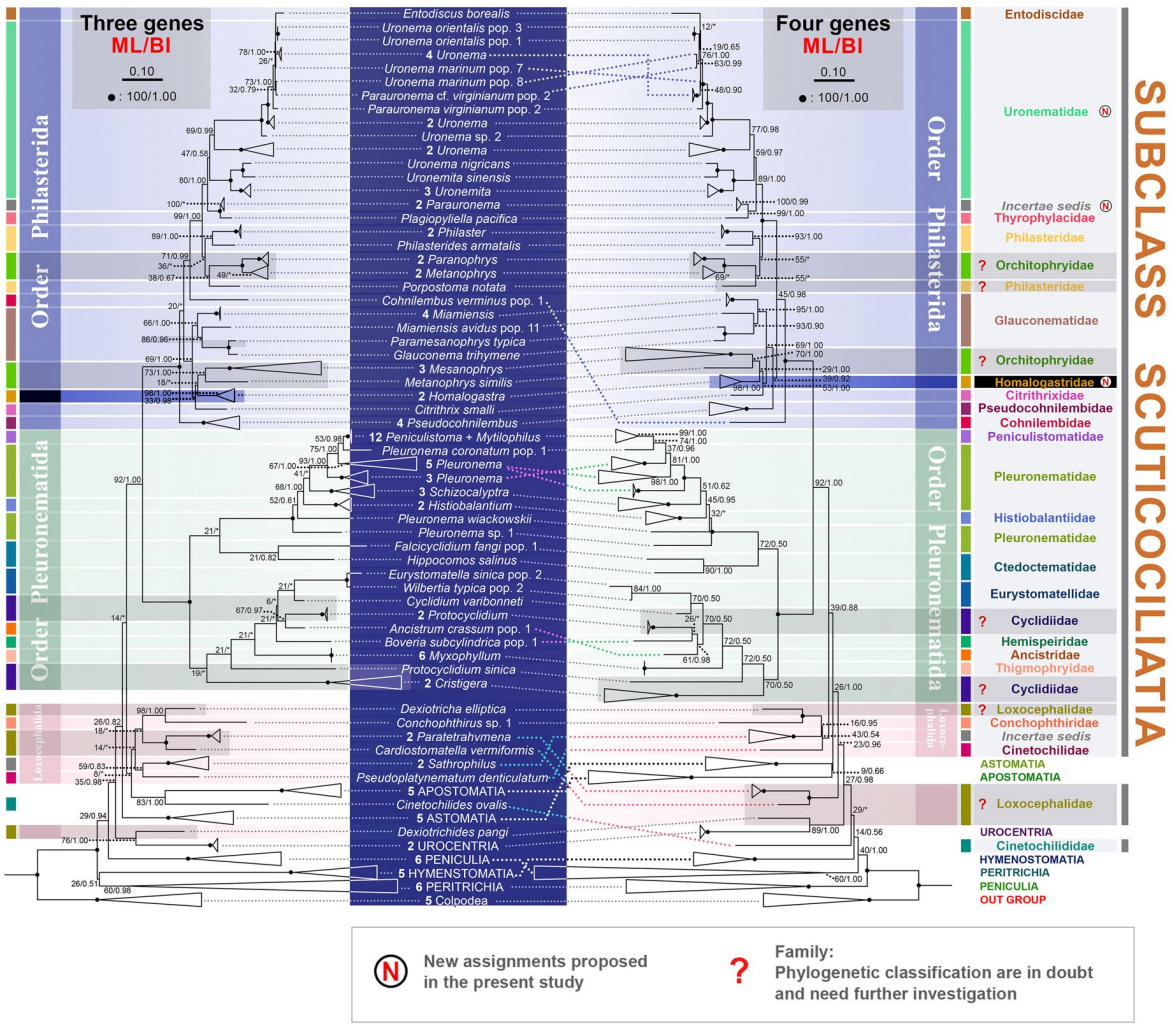
Maximum likelihood (ML) tree based on concatenated gene sequence data. In the three-gene tree, the sequences are concatenated in the order: SSU rRNA gene, LSU rRNA gene and ITS1-5.8S-ITS2 gene region; in the four-gene tree, the order is: *COI* gene, SSU rRNA gene, LSU rRNA gene and ITS1-5.8S-ITS2 gene region. The SSU rRNA gene sequence is available for all the taxa whereas the *COI* gene, LSU rRNA gene and ITS1-5.8S-ITS2 gene region are available for only a subset of these taxa (Supplementary Table S2). Numbers at nodes denote ML bootstrap values/Bayesian inference (BI) posterior probabilities. Asterisks (*) indicate a mismatch in topologies between ML and BI analyses. Fully supported (100%/1.00) nodes are marked with solid circles. The scale bar corresponds to 10 substitutions per 100 nucleotide positions. All branches are drawn to scale. The systematic classification mainly follows Gao et al. (2016). New assignments proposed in the present study are marked with the letter “N” in the circles. Family names with question marks indicate families that are polyphyletic and need further investigation in future studies

The topologies of three-gene (SSU rRNA, LSU rRNA, and ITS1-5.8S-ITS2 region) and four-gene (*COI*, SSU rRNA, LSU rRNA and ITS1-5.8S-ITS2 region) phylogenetic trees basically match well with that of SSU rRNA tree (Fig. 5). The order Loxocephalida and the family Loxocephalidae remain polyphyletic in the multi-gene trees.

The order Pleuronematida forms a fully supported clade in both the three-gene and the four-gene trees. Histiolalantiidae, Peniculistomatidae and Pleuronematidae group together in both trees, which is identical to the topology of the SSU rRNA tree (Fig. 5). The main difference between the concatenated trees and the SSU rRNA tree is that the family Eurystomatellidae does not cluster with Ctedoctematidae but is located within the Cyclidiidae + Hemispeiridae + Ancistridae + Thigmophryidae clade (Fig. 5).

The order Philasterida is monophyletic with full support in the concatenated trees (Fig. 5). Uronematidae, Philasteridae and Orchitophryidae are polyphyletic, which is consistent with the SSU rRNA tree. The concatenated trees differ from the SSU rRNA tree mainly in the positions of the families Cohnilembidae and Pseudocohnilembidae. In the three-gene tree, Pseudocohnilembidae branches first within the Philasterida clade, whereas in the four-gene tree, Cohnilembidae occupies the basal position of the Philasterida clade and Pseudocohnilembidae locates next to the Citrithrixidae-Homalogastridae-*Mesanophrys*-*Metanophrys similis*-Glauconematidae group (Fig. 5). In the SSU rRNA tree, Cohnilembidae is sister to the *Metanophrys similis* and *Paranophrys marina* clade, whereas Pseudocohnilembidae is sister to the *Madsenia* + *Paranophrys* + *Metanophrys* + *Biggaria* clade (Fig. 3).

### Topology of the SSU rRNA gene tree revealed by a combination of well-identified and environmental sequences of Scuticociliatia

In the GenBank database, there are many SSU rRNA gene sequences from environmental samples that are assigned to Scuticociliatia but lack detailed identification or vouchered specimens. These sequences were excluded from most previous phylogenetic analyses. In this study, we constructed a maximum likelihood tree with both environmental and well-identified sequences (for the sequence information, see Supplementary Table S3).

**Fig. 6:**
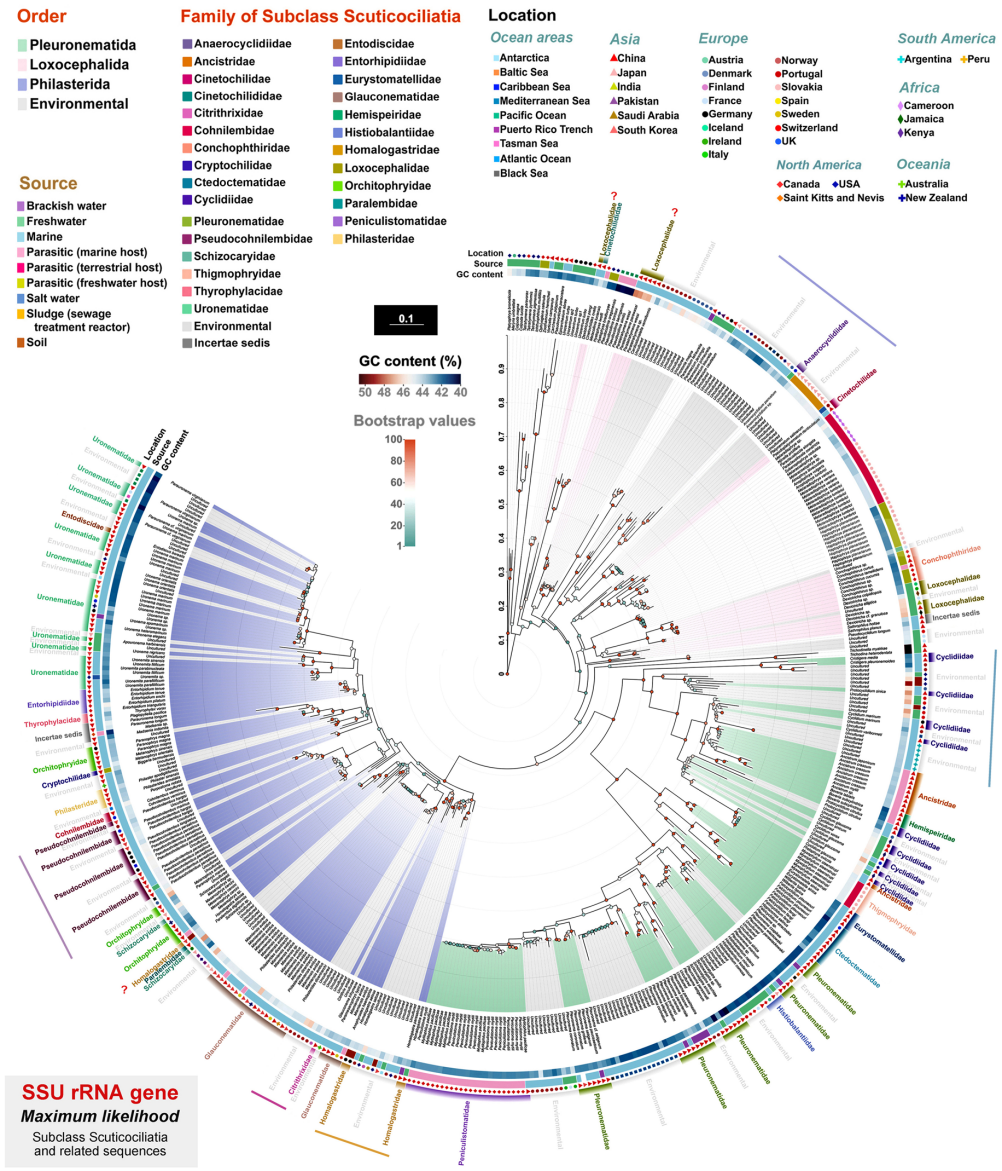
Maximum likelihood (ML) tree based on SSU rRNA gene sequence data of Scuticociliatia (in colored blocks), other subclass (with no blocks) and related environmental sequences (in grey blocks). ML bootstrap values and G + C content (%) are represented by different colors. Source and location information are obtained from the NCBI database or related references. The scale bar corresponds to 10 substitutions per 100 nucleotide positions. All branches are drawn to scale. The systematic classification mainly follows Gao et al. (2016). Family names with question marks indicate the identity of the sequences or family assignment of the species should be confirmed. Straight lines after family names and environmental sequences indicate those environmental sequences are possibly members of the corresponding families. For detailed sequence information, see Supplymentary Table S3

In total, 147 environmental sequences were included, 121 of which group within the Scuticociliatia (Fig. 6). Fourteen environmental sequences cluster with the 24 Loxocephalida sequences, among which 12 cluster with *Cardiostomatella vermiformis* (98% ML), and two cluster with *Conchophthirus* and *Dexiotricha* (100% ML) (Fig. 6). A total of 59 environmental sequences cluster with 107 Pleuronematida sequences, accounting for 35.5% of representatives of this order. *Pseudocyclidium longum* is sister to the Pleuronematida + Philasterida clade, and two sequences from Peritrichia (*Trichodina* and *Trichodinella*) are located between *Pseudocyclidium longum* and Pleuronematida + Philasterida clade.

The environmental sequences that cluster within the clade of Pleuronematida do so with relatively low supporting values and most are located near Cyclidiidae and Pleuronematidae-Histiobalantiidae-Peniculistomatidae (21 and 35, respectively) (Fig. 6). The order Philasterida contains 48 environmental sequences. Among these, 10 clusters with *Homalogastra*, and six clusters with *Citrithrix smalli*, forming two clades with high support values (92% and 99% in ML, respectively) (Fig. 6).

### Oral structures of taxa in subclass Scuticociliatia

**Fig. 7:**
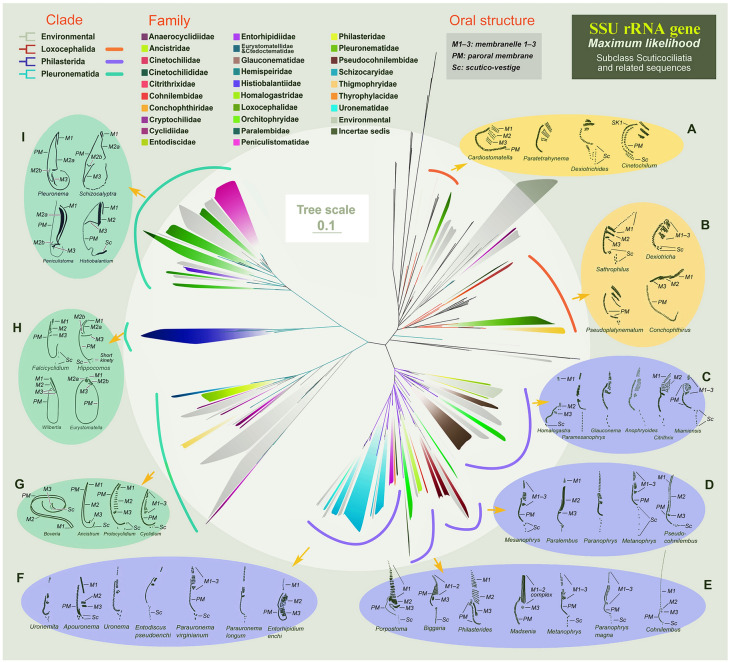
Collapsed maximum likelihood tree of Scuticociliatia based on SSU rRNA gene sequence data with schematic oral structure diagrams of some representative members of the three Scuticociliatia orders. The colored clades represent family assignment, and the colored lines at the outer side of the clades represent order assignment. The scale bar corresponds to 10 substitutions per 100 nucleotide positions. All branches are drawn to scale. The systematic classification mainly follows Gao et al. (2016). M1–3, membranelle 1–3; PM, paroral membrane; Sc, scutico-vestige; SK, somatic kinety

Based on previous work, we here summarize and provide redrawn schematic diagrams of the oral structure of representative members of the three orders of Scuticociliatia (Fig. 7). In general, the oral structure of Scuticociliatia is composed of three oral membranelles (M1–3) and a paroral membrane (PM). In Loxocephalida, the basal bodies of M1–3 are arranged obliquely, and the basal bodies of the PM are arranged in an arc-shape. M1–3 of *Cardiostomatella* and *Paratetrahymena* are almost equal in length, and each membranelle has two or three rows of basal bodies while the lengths of M1–3 vary in both *Dexiotrichides* and *Cinetochilum*, and a multi-rowed scutico-vestige (Sc, the residual field of recognizable scutico-kinetosomes) appears below PM (Fig. 7A). The M1–3 of *Sathrophilus*, *Dexiotricha*, *Pseudoplatynematum* and *Conchophthirus* are arranged obliquely or transversely, and further variations appear in membranelle length and structure (Fig. 7B).

The oral membranelles of Philasterida are vertically arranged. In *Anophryoides*, *Citrithrix*, and *Miamiensis*, the M2 forms a remarkable horizontally oriented multi-rowed structure, which is not observed in their closely related genera such as *Homalogastra*, *Paramesanophrys*, and *Glauconema* (Fig. 7C). Most other Philasterida genera generally have a one- to three-rowed M1, and a two- to four-rowed M2 (Fig. 7D–F). Specifically, *Philasterides*, *Porpostoma*, and *Biggaria* (Fig. 7E) have a well-developed M1 and M2, and *Entorhipidium* (Fig. 7F) has a well-developed M2, each with multiple transverse rows. The oral structure of *Cohnilembus*/*Pseudocohnilembus* is highly specialized since when compared to other philasterids, the M1 of *Cohnilembus* is prominently long while M2 is reduced, and the M1 and M2 of *Pseudocohnilembus* are significantly elongated and commence anteriorly at the about same level (Fig. 7D, E). In members of the clade that includes *Uronema*, *Uronemita* and *Parauronema*, the oral structure is further simplified, and the M1 usually has only one or two basal body rows (Fig. 7F).

The oral membranelles of Pleuronematida are more complex and the paroral membrane is quite prominent. The oral structure of Cyclidiidae genera, such as *Cyclidium* and *Protocyclidium*, is similar to that of Loxocephalida, with the M1–3 not significantly specialized, and the double-rowed PM located on the right side of the membranelles (Fig. 7G). However, the membranelles of Cyclidiidae are vertically arranged. Another Pleuronematida clade that comprises *Falcicyclidium*, *Hippocomos*, *Wilbertia*, and *Eurystomatella*, has elongated and multi-rowed M1–3. In addition, their PM is prominently developed, and the posterior end of the PM in the latter two genera extends downwards and then turns upward, forming a ring structure around the oral apparatus (Fig. 7H). In other representatives of Pleuronematida, i.e., *Pleuronema*, *Schizocalyptra*, *Peniculistoma*, and *Histiobalantium*, the M1–3 and PM are well developed, and the M2 in some genera is bipartite (Fig. 7I).

### Comparison of putative secondary structure of ITS2* region*

**Fig. 8:**
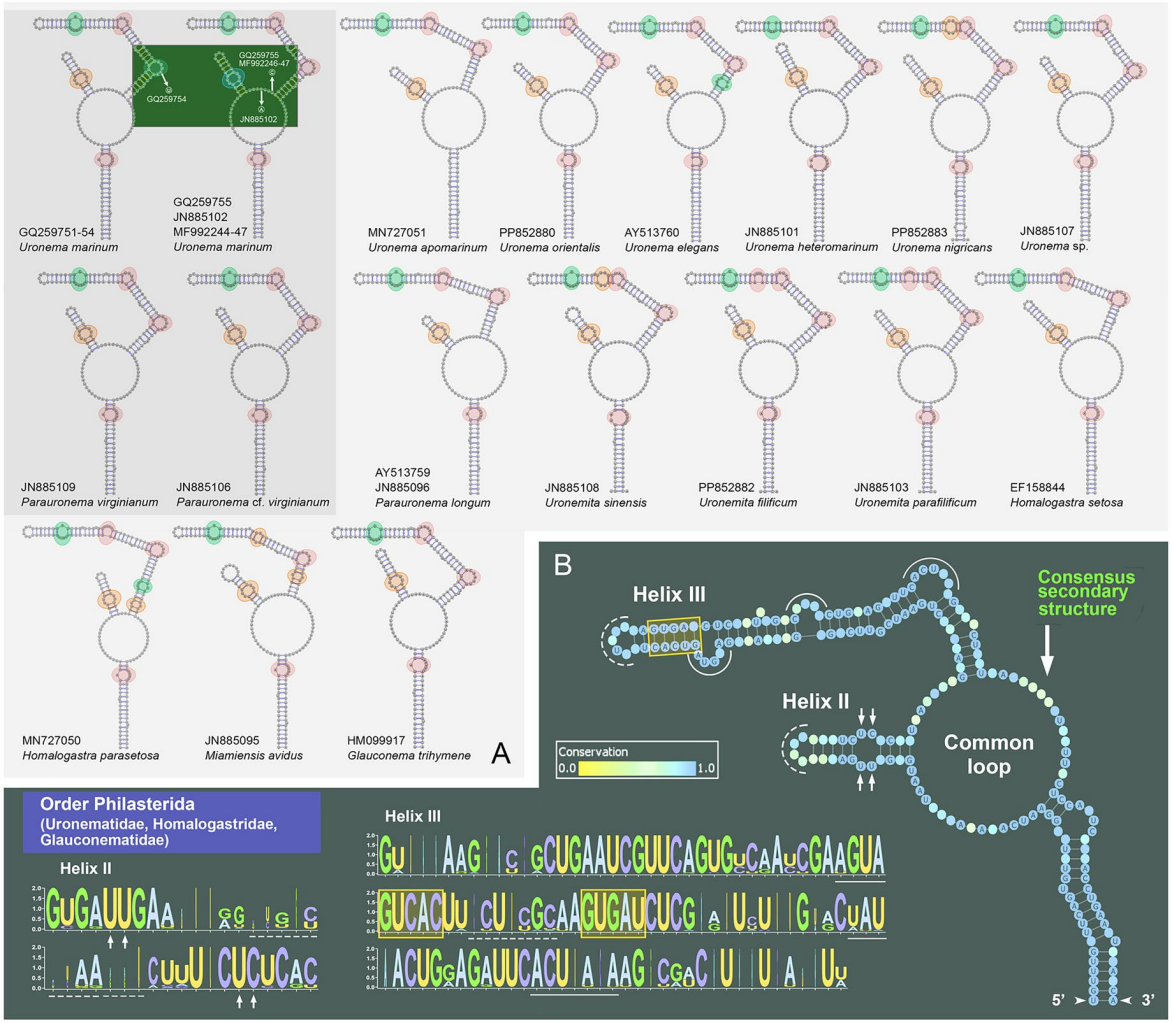
Comparison of putative ITS2 region secondary structure of the taxa from Uronematidae, Homalogastridae, and Glauconematidae of the order Philasterida (**A**) and the consensus secondary structure based on these selected sequences, with nucleotides composition in different helixes (**B**). Colored circles in A indicate bulges with at least two nucleotides. Yellow circles represent bulges with the same number of nucleotides at both sides, green circles represent bulges with more nucleotides at 5’ ends than 3’ ends of ITS2 region, and pink circles represent bulges with more nucleotides at 3’ ends than 5’ ends of ITS2 region. Small arrows in B indicate pyrimidine-pyrimidine mismatch of Helix II, box indicates the highly conserved motif in Helix III, dash lines indicate terminal bulges in helixes, and solid lines indicate bulges in helixes with more than one nucleotide. Fully conserved positions of the consensus structure are marked with specific nucleotides

The secondary structures of the ITS2 region of Philasterida, Pleuronematida and Loxocephalida were predicted using Mfold (Fig. 8; Supplementary Figs. S1–S3). The topological characteristics are summarized in Supplementary Tables S5 and S6. The length of the ITS2 region ranges from 130 to 242 nucleotides (nt), with an average length of 176 nt (*n* = 64), and the G + C content ranges from 30.46% to 50.28% (average 37.76%).

Generally, the ITS2 secondary structures of these three orders are all composed of a common loop and at least two unequal-length helices, corresponding to Helix II and III. In Helix II, all taxa of these three orders possess a pyrimidine-pyrimidine mismatch (mostly 5′-UU vs. UC-3′), which is located after the first four pairs of nucleotides. Helix III is longer and more variable than Helix II (Supplementary Tables S5, S6). Helix III also has a highly conserved motif (5′-GUCAC vs. GUGAU-3′) and a bulge (usually 5′-AGUA vs. C-3′) located next to this motif (Fig. 8; Supplementary Figs. S1–S3).

In Philasterida, the ITS2 secondary structure of 42 species/populations was predicted (Fig. 8; Supplementary Fig. S1, Tables S5, S6). All of these have two helices, i.e., Helix II and III. The length of Helix II is from 22 to 32 nt (27 nt on average) while that of Helix III is from 89 to 113 nt (94 nt on average) (Supplementary Table S5). It is noteworthy that the ITS2 secondary structures of *Parauronema virginianum* (JN885109) and *Parauronema* cf. *virginianum* (JN885106) are identical with those of ten populations of *Uronema marinum* in terms of the length of both helices and the central loop, and the number of unpaired bases in each helix (Fig. 8A; Supplementary Table S6). For the nucleotide composition of the ITS2 region, *P. virginianum* (JN885109) and *P.* cf. *virginianum* (JN885106) differ from six *Uronema marinum* sequences (GQ259755, JN885102, MF992244–47) in only one or two positions (sequence identity 99.5% or 99.0%, respectively), whereas compared to *Uronema marinum* (GQ259751–54), *P. virginianum* (JN885109) and *P.* cf. *virginianum* (JN885106) have eight or nine nucleotide differences (sequence identity 96.0% or 95.5%). The number of unmatched nucleotides of ITS2 within the ten *Uronema marinum* sequences ranges from zero to eight (sequence identity 96.0–100%) (Supplementary Table S7).

For the order Pleuronematida, the secondary structures of 15 species/populations were predicted (Supplementary Fig. S2, Tables S5, S6). Among these, *Schizocalyptra sinica* JX310010 and *Protocyclidium citrullus* (KF256832, KF256833) each possess three helices (Helix I, II and III in *S. sinica*; Helix II, III and IV in *P. citrullus*), while others only have Helix II and III (Supplementary Fig. S2).

The ITS2 secondary structures of seven species in the order Loxocephalida were predicted (Supplementary Fig. S3, Tables S5, S6). Five of these possess Helix IV (11–20 nt) though it is not conserved and does not appear in the consensus secondary structure (Supplementary Fig. S3). The secondary structure of *Cinetochilides ovalis* (JX310025) lacks Helix IV but has a furcation loop in Helix III (Supplementary Fig. S3A) which is unique to Loxocephalida. There is no variation in the motif of Helix III (5′-GUCAC vs. GUGAU-3′) among the seven Loxocephalida species (Supplementary Fig. S3).

## Discussion

### Multi-gene-based phylogeny and systematic reconsideration of the subclass Scuticociliatia

The classification of several groups within the subclass Scuticociliatia has been unclear and confused for a long time due to their homogeneity in morphology and polyphyly in molecular phylogeny. Previous phylogenetic analyses revealed that the families in Scuticociliatia are well grouped at the order level and that Philasterida and Pleuronematida are generally well outlined, while Loxocephalida often clusters with members from other subclasses and likely represents a relatively primitive group of scuticociliates (Antipa et al. 2020; Gao et al. 2012a, 2013; Poláková et al. 2021, 2023; Qu et al. 2020; Zhang et al. 2019). In this study, we obtained more sequences of Scuticociliatia and performed in-depth phylogenetic analysis. The results matched well with previous studies and confirmed the polyphyly of several taxonomic groups (e.g., Parauronematidae, Uronematidae, Philasteridae). Furthermore, we found the identity or classification of some taxa should be reconsidered.

Parauronematidae and Uronematidae are two typical families of Philasterida with similar morphological characteristics, but phylogenetic analyses repeatedly revealed they were polyphyletic, as *Glauconema* and *Miamiensis* were separated from the type genus *Parauronema*, and *Parauronema virginianum* clustered closely with *Uronema marinum*, which is the type species of *Uronema* (Uronematidae) (Bourland et al. 2024; Hao et al. 2022; Li et al. 2024a; Liu et al. 2020b). The reassignment of the members of Parauronematidae has been proposed in previous studies (Foissner 1971; Gao et al. 2012a, b; Ma et al. 2018; Zhang et al. 2019), however, some of these taxa were ambiguous or not fully discussed, which suggests that reinvestigations based on morpho-molecular data are needed. Another genus of Uronematidae, *Homalogastra*, was clearly separated from the core members of Uronematidae, indicating that it may potentially represent a family-level group in the order Philasterida. In addition, the classification of *Madsenia*, *Protophrya*, and *Pseudocyclidium* needs to be discussed as they are separated from the related sequences of their families in SSU rRNA gene trees. Here we provide a reconsideration of the classification of the aforementioned genera along with other members of Scuticociliatia based on morphological and molecular evidence.

In addition, there are several families (e.g., Orchitophryidae in Philasterida, Cyclidiidae in Pleuronematida, and Loxocephalidae and Cinetochilidae in Loxocephalida) which are polyphyletic, their members being dispersed within their respective orders, thus the assignment, classification, and/or validity of some species/genera are in doubt. The phylogeny of these four families, i.e., Orchitophryidae, Cyclidiidae, Loxocephalidae and Cinetochilidae, is beyond the scope of this study as the molecular data are largely insufficient and would benefit from reinvestigations in future studies.

### *Parauronema* is transferred from Parauronematidae to Uronematidae as a valid genus, with remarks on *Parauronema virginianum*/*P. *cf. *virginianum*

The genus *Parauronema* was established by Thompson (1967) with the description of its type species *P.* *virginianum*, while its family-level assignment was not mentioned. In the classification of Corliss (1979), *Parauronema* was assigned to the family Philasteridae Kahl, 1931. Subsequently, Small and Lynn (1985) established the family Parauronematidae for *Parauronema* and related genera, though the type genus of this family was not assigned. In Lynn’s classification (2008), the diagnosis of Parauronematidae was modified and the classification of *Parauronema* remained consistent with that in Small and Lynn (1985).

In all the phylogenetic trees of the present work, *Parauronema virginianum* and *P*. cf. *virginianum* grouped with *Uronema*, forming the well-supported *Uronema* + *Parauronema* + *Entodiscus* clade (Figs. 3, 4, 5 and 6). This is consistent with previous phylogenetic studies based on SSU rRNA or LSU rRNA genes that show *P*. *virginianum* and *P*. cf. *virginianum* consistently clustering with *Entodiscus borealis* and *Uronema* (Gao et al. 2012a, b; Hao et al. 2022; Liu et al. 2020b; Pan et al. 2015; Zhang and Vďačný 2021).

For SSU rRNA gene sequence comparison, two *Parauronema virginianum* sequences (AY392128, JN885087; sequence identity 99.3% with 10 unmatched nucleotides) differ from two *P*. cf. *virginianum* sequences (FJ595488, JN885082; sequence identity 99.2% with 11 unmatched nucleotides) by 8–13 nucleotides (sequence identity 99.1–99.4%) (Supplementary Table S8). When compared with the *Uronema* sequences from the same clade, *P*. *virginianum* (AY392128, JN885087) differs from them by 2–11 nucleotides (identity 99.2–99.8%), while *P*. cf. *virginianum* (FJ595488, JN885082) differs from them by 5–12 nucleotides (identity 99.1–99.6%) (Supplementary Table S8). Specifically, *P*. *virginianum* (AY392128) is the closest sequence to *Uronema* sp. (KY569295) and *Uronema marinum* (GQ259749), with only two and three unmatched nucleotides, respectively (sequence identity 99.7–99.8%) (Supplementary Table S8). The ITS2 region of *P*. *virginianum* and *P*. cf. *virginianum* each has one or two nucleotide differences when compared with *U. marinum* (GQ259754–55, JN885102, MF992244–47), while it is identical with other ITS2 sequences of *Uronema marinum*.

A key differentiating character between *Uronema* and *Parauronema* is the structure of M1, which is single-rowed in the former and two-rowed in the latter. However, a recent study revealed that some members of *Uronema* possess a partly two-rowed M1 (Liu et al. 2020a). Considering that the type species of *Parauronema*, *P. virginianum*, has a close relationship with *Uronema* in phylogenetic analyses (Hao et al. 2022; Liu et al. 2020a, b), it has been suggested that *Parauronema* should become a junior synonym of *Uronema*, the type species *P. virginianum* should be transferred to *Uronema*, and other genera currently classified in Parauronematidae should be transferred to Uronematidae (Foissner 1971; Gao et al. 2012a; Pan et al. 2015). However, some of these designations seem premature considering their divergence in morphology and phylogeny.

Morphologically, *Parauronema virginianum*/*P*. cf. *virginianum* resembles *Uronema* in the general pyriform to ovoid cell shape with a truncated apical plate, M1 and M2 that are about equal in length and closely juxtaposed, and Y-shaped scutico-vestige (Fig. 7F) (Pan et al. 2015; Song and Wilbert 2000; Thompson 1967). The morphological differences between them are not significant and mainly lie in the structure of M1, the distance between M1 and M2, and the appearance of the apical plate (Gao et al. 2012a; Liu et al. 2020a; Song et al. 2009). *Uronema* has a single-rowed or partly two-rowed M1 while *P*. *virginianum* has fully two-rowed M1 (Liu et al. 2020a). In addition, M1 and M2 are clearly separated in the former, the gap being equivalent to about twice the length of M1, whereas in *P*. *virginianum*, the gap is shorter than the length of M1 (Fig. 7F). Furthermore, the apical plate of *Uronema* is prominent, whereas in *P*. *virginianum* it is inconspicuous (Liu et al. 2020a; Pan et al. 2015; Song and Wilbert 2000). Considering the close phylogenetic relationship and high sequence similarity between the type species of *Parauronema* and *Uronema*, here we formally transfer *Parauronema* to the family Uronematidae. However, due to the obvious divergence in their morphology mentioned above, we do not agree that *Parauronema* is a synonym of *Uronema* (Table 3) but prefer to retain *Parauronema* as a valid genus (Foissner 1971; Gao et al. 2012a).

**Table 3:** Previous and present proposed classifications of related members from the order Philasterida

A. Lynn (2008)^a^	B. Present study
A1. Parauronematidae	B1. Uronematidae
*1. Glauconema*	*1. Parauronema*
*2. Miamiensis*	*2. Pseuduronema*
*3. Parauronema* (to B1.1)	*3. Urocyclon*
*4. Potomacus* (to B6.2)	*4. Uronema*
	*5. Uronemita*
A2. Uronematidae	*6. Uronemopsis*
*1. Homalogastra* (to B2.1)	*7. Uropedalium*
*2. Pseuduronema*	
*3. Urocyclon*	B2. Homalogastridae fam. nov
*4. Uronema*	*1. Homalogastra*
*5. Uronemita*	
*6. Uronemopsis*	B3. Philasteridae
*7. Uropedalium*	*1. Helicostoma*
	*2. Kahlilembus*
A3. Philasteridae	*3. Paraphilaster*
*1. Helicostoma*	*4. Philaster*
*2. Kahlilembus*	*5. Philasterides*
*3. Madsenia* (to B6.1)	*6. Porpostoma*
*4. Paraphilaster*	
*5. Philaster*	B4. Glauconematidae
*6. Philasterides*	*1. Anophryoides*
*7. Porpostoma*	*2. Glauconema*
	*3. Miamiensis*
A4. Orchitophryidae	*4. Paramesanophrys*
*1. Anophryoides*	
*2. Mesanophrys*	B5. Orchitophryidae
*3. Metanophrys*	*1. Mesanophrys*
*4. Orchitophrya*	*2. Metanophrys*
*5. Paranophrys*	*3. Orchitophrya*
	*4. Paranophrys*
	B6. *Incertae sedis* in Philasterida
	*1. Madsenia*
	*2. Potomacus*

^a^Genera with new assignments are annotated with their positions in Column B

### Remarks on *Parauronema longum*

Unlike *Parauronema virginianum* and *P.* cf. *virginianum*, *P. longum* clusters with Thyrophylacidae and/or Entorhipidiidae in the present phylogenetic trees, but it can be easily separated from these two families by cell shape (cylindrical, not laterally flattened in *P*. *longum* vs. laterally flattened, with caudal projection in the latter two families) and living style (free-living in *P*. *longum* vs. endocommensal in the intestines of sea urchins/echinoids) (Long et al. 2007; Lynn 2008; Lynn and Strüder-Kypke 2005). *Parauronema longum* can be separated from another related family, Orchitophryidae, by having a prominent apical plate (vs. no apical plate in Orchitophryidae), a relatively sub-anteriorly positioned M1 and cytostome (vs. anteriorly positioned in Orchitophryidae), and a Y-shaped scutico-vestige (vs. linear in Orchitophryidae) (Lynn 2008; Song 1995; Song et al. 2009).

*Parauronema longum* has a cylindrical cell shape and its M1 is about twice the length of M2 (Fig. 7F), which differs from the type species *Parauronema virginianum* (cell pyriform to ovoid in shape, M1 about equal to M2 in length) (Pan et al. 2015; Song 1995; Song and Wilbert 2000; Song et al. 2009). Morphologically, *P. longum* resembles the family Uronematidae in having a prominent apical plate, M1 and M2 that are clearly separated from each other, a Y-shaped scutico-vestige, an anteriorly positioned oral field, and its cytostome located in the mid-body region (Lynn 2008; Song and Wilbert 2000; Song et al. 2009). However, when compared with the type genus *Uronema* and other members of Uronematidae, *P*. *longum* has a more elongated, cylindrical body (vs. basically ovoid to pyriform in Uronematidae) and a longer M1 (seven to 11 pairs of basal bodies in *P*. *longum* vs. usually five or six basal bodies arranged in single row) (Lynn 2008; Song and Wilbert 2000; Song et al. 2009). In addition, phylogenetic analyses show that *P*. *longum* is clearly separated from the “core” Uronematidae sequences (Figs. 3, 4B). In summary, the morphological and molecular data both suggest the separation of *P. longum* from *P*. *virginianum*, and do not support the transfer of *P*. *longum* to Uronematidae. Thus, here we consider *P*. *longum* as *incertae sedis* within the order Philasterida.

### *Potomacus* should be treated as* incertae sedis* within the order Philasterida

*Potomacus* was established with the description of *P. pottsi* (Thompson 1966) which was primarily characterized by its polymorphic size and forms. Two microstome and one macrostome forms were reported. One microstome form is non-tailed with a pointed anterior end and rounded posterior end. The other microstome form is tailed and is pointed at both ends. The macrostome form may be pointed or rounded in either end, and is much larger in size when compared with the two microstome forms (Thompson 1966).

*Potomacus* was originally assigned to the family Uronematidae based on the three small linearly arranged oral membranelles and the general characteristics of the buccal apparatus (Thompson 1966). Corliss (1979) assigned *Potomacus* to Philasteridae, but it was subsequently transferred to the family Parauronematidae based on its cell shape, general oral apparatus, and microstome-macrostome transformation during its life cycle (Lynn 2008; Small and Lynn 1985).

Ramsey et al. (1980) reported a population under “*Potomacus pottsi*” with a description of its morphology and stomatogenesis. However, the oral apparatus of its microstome trophont differs from that of the original population, especially in the anterior position of the PM (at mid-portion of M1 vs. at mid-portion of M2 in the original population), the structure of M1 (short, about four-rowed vs. relatively longer and narrowed, in a trianglular pattern in the original population) and the shape of the scutico-vestige (generally linear vs. Y-shaped in the original population). Therefore, the identification of this population remains doubtful.

After reinvestigating *Potomacus*, we found that *Potomacus* matches the characteristics of the family Uronematidae in cell shape (basically pyriform in representative cells), and the oral apparatus (three linear membranelles and a *Uronema*-like PM), which strongly resembles that of *Uronema,* the type genus of Uronematidae (Lynn 2008; Song et al. 2009; Thompson 1966). However, *Potomacus* possesses a pointed anterior end with its M1 anteriorly located, which resembles the family Orchitophryidae (Cawthorn et al. 1996; Lynn 2008; Song and Wilbert 2000; Thompson 1966). Molecular data on *Potomacus* is still lacking. Based on the current morphological data, we suggest treating *Potomacus* as *incertae sedis* within the order Philasterida until more detailed morphological and molecular information is available (Table 3).

### Parauronematidae is proposed as a junior synonym of Uronematidae

The definition of Parauronematidae and Uronematidae share many morphological features, such as cell size and shape (small, pyriform to ovoid), an anteriorly positioned oral region in a shallow cavity, and a basically linear arrangement of the oral membranelles (Lynn 2008; Small et al. 1985). The main difference between them is that several genera from the former family include the microstome-macrostome transformation, which is not reported in Uronematidae (Lynn 2008). In the present study, the type genus of Parauronematidae, *Parauronema*, is transferred to Uronematidae based both on its morphological features (especially the oral structure) and molecular phylogenetic analyses, while another genus, *Potomacus* is treated as *incertae sedis* within the order Philasterida. In a recent study, the other two Parauronematidae genera, *Glauconema* and *Miamiensis*, along with two Orchitophryidae genera, *Paramesanophrys* and *Anophryoides*, were formally assigned to a new family Glauconematidae (Li et al. 2024a). Our results support this finding (Table 3). Consequently, all four genera of Parauronematidae are transferred to other families, so we suggest the family Parauronematidae Small and Lynn, 1985 to be a junior synonym of Uronematidae Thompson, 1964.

### Separation of *Homalogastra* from Uronematidae and establishment of Homalogastridae fam. nov.

The genus *Homalogastra* was established by Kahl (1926) based on the description of a new species, *H. setosa,* which has a spindle-like cell shape with a subequatorial cytostome. Its anterior end is slightly truncated while the posterior part gradually tapers, the widest part of the cell being in the pre-equatorial region. This genus now comprises three nominal species, *H. setosa* Kahl, 1926, *H. parasetosa* Liu et al., 2020 and *H. similis* (Liu et al., 2020) Liu et al., 2021.

In most classification systems, *Homalogastra* has been assigned to the family Uronematidae Thompson, 1964 based on its small size, non-ciliated apical pole and the presence of a caudal cilium (Corliss 1979; Puytorac 1994; Lynn 2008; Small et al. 1985). However, in phylogenetic trees, *Homalogastra* rarely clusters within Uronematidae regardless of the gene markers used for analysis (Figs. 3–6). Moreover, there are significant morphological differences between *Homalogastra* and Uronematidae, such as cell shape, the position of the cytostome, and the scutico-vestige with two or three basal bodies in *Homalogastra* (vs. Y-shaped in Uronematidae) (Kahl 1928, 1931; Liu et al. 2020a, 2020b; Lynn 2008; Small et al. 1985; Song et al. 2009). Previous studies also mentioned discrepancies in morphogenesis and molecular phylogeny, which have questioned the assignment of *Homalogastra* to Uronematidae (Gao et al. 2012a; Pomp and Wilbert 1988). Considering the morphological differences and phylogenetic deviation, here we suggest that *Homalogastra* should be removed from the family Uronematidae. It is noteworthy that *Uropedalium*, a genus in Uronematidae, has similar morphological features to *Homalogastra*, however its molecular data are not available, hence, we retain *Uropedalium* in Uronematidae pending further information (Table 3).

In terms of cell size and shape, somatic ciliature and oral structure, two other related families, namely Orchitophryidae and Citrithrixidae, should be considered for the assignment of *Homalogastra*. *Homalogastra* can be distinguished from Orchitophryidae by possessing a small, truncated apical plate (vs. pointed and without apical plate in Orchitophryidae), spiral grooves on the pellicle (vs. longitudinal grooves in Orchitophryidae), significantly reduced M1 and M2 that are widely spaced (Fig. 7C) (vs. multi-rowed M1 and M2 that are closely juxtaposed in Orchitophryidae; Fig. 7E, D), and the structure of the scutico-vestige (two or three basal bodies in *Homalogastra* vs. basically linear or Y-shaped in Orchitophryidae). In addition, *Homalogastra* differs from Orchitophryidae in the subequatorial (vs. anterior) position of the cytostome (Liu et al. 2020a, b; Lynn 2008; Song et al. 2009).

*Homalogastra* resembles Citrithrixidae in having a spindle-like cell shape and a subequatorial cytostome located in a depression (Liu et al. 2020a, b; Lynn 2008; Song et al. 2009). Nevertheless, *Homalogastra* can be separated from Citrithrixidae by its spiral (vs. longitudinal) pellicular grooves, its significantly reduced and widely separated M1 and M2 (vs. M1 and M2 multi-rowed and closely juxtaposed in Citrithrixidae) (Fig. 7C), and the structure of the scutico-vestige which comprises two or three basal bodies (vs. two or three pairs of basal bodies in Citrithrixidae) (Liu et al. 2020a, b; Lynn 2008; Song et al. 2009).

According to both the morphological and molecular data available, *Homalogastra* does not correspond to the diagnosis of Uronematidae or other known families in Philasterida, and therefore represents an undescribed taxon at family level. Hence a new family, Homalogastridae fam. nov., is suggested. This new monotypic family currently contains only the taxa assigned in the genus *Homalogastra*.

*Taxonomic assignment*: phylum Ciliophora: subphylum Intramacronucleata: class Oligohymenophorea: subclass Scuticociliatia: order Philasterida.

Homalogastridae fam. nov.

*ZooBank registration*: urn:lsid:zoobank.org:act:FA2A74A2-FC15-4FE1-A459-8C6C90919E30.

*Diagnosis*: free-living philasterids; cell small, typically spindle-shaped with an apical plate; three reduced, well-outlined oral polykinetids, cytostome conspicuously subequatorially positioned; scutico-vestige short and inconspicuous.

*Type genus*: *Homalogastra* Kahl, 1926

### *Madsenia* is regarded as* incertae sedis* in the order Philasterida

*Madsenia* was established by Kahl (1934) as *incertae sedis.* Jankowski (1973) established the family Entodiscidae to contain *Madsenia* and other related genera, but Corliss (1979) subsequently re-assigned *Madsenia* to the family Philasteridae.

*Madsenia* is mainly characterized by its bilaterally flattened, slender and elongate cell shape, narrow or truncated anterior end, pointed posterior end, and pre-equatorial cytostome (Long et al. 2007). There are several features that separate *Madsenia* from the family Philasteridae. *Madsenia* has a prominent apical suture formed by the anterior ends of the somatic kineties, which is not observed in the species of Philasteridae. The M1 and M2 form a complex in *Madsenia* whereas the M1 is triangular and is clearly separated from M2 in Philasteridae (Fig. 7E). Moreover, *Madsenia* is a genus of marine endocommensal forms whereas philasterids are predominantly free-living (Long et al. 2007; Lynn 2008). In the present SSU rRNA trees (Figs. 3, 6), *Madsenia* clusters with members of Orchitophryidae, rather than with the members of Philasteridae, which is consistent with previous studies (Liu et al. 2016, 2017, 2020a, b). Based on a combination of morphological and molecular information, we suggest *Madsenia* should be removed from the family Philasteridae.

*Madsenia* shares characteristics with Entodiscidae and Entorhipidiidae in the bilaterally flattened cell, narrowed and concave oral region that is anteriorly located, and its marine endocommensal lifestyle (Long et al. 2007; Lynn 2008). Nevertheless, *Madsenia* can be distinguished from Entodiscidae or Entorhipidiidae in the general cell shape (slender and elongated in *Madsenia*, posterior end pointed but with no tail vs. usually oval in Entodiscidae, sigmoid with anterior folded and posterior forming to a tail in Entorhipidiidae) and the structure of the oral apparatus (M1 and M2 incorporated into a single structure in *Madsenia* vs. *Uronema*-like, M1 and M2 with clear separation in Entodiscidae and Entorhipidiidae) (Fig. 7E, F) (Long et al. 2007; Lynn 2008).

*Madsenia* can be distinguished from the phylogenetically related family Orchitophryidae by its cell shape (bilaterally flattened, anterior narrow or truncated, posterior narrowed and pointed in *Madsenia* vs. pyriform to ovoid, not laterally flattened, anterior usually pointed, posterior rounded in Orchitophryidae), the presence of an apical suture (vs. absent in Orchitophryidae), and the structure of the oral apparatus (M1 and M2 incorporated into a single structure, forming a complex in *Madsenia* vs. M1 and M2 well separated in Orchitophryidae) (Fig. 7D, E) (Long et al. 2007; Lynn 2008; Song and Wilbert 2000; Song et al. 2009). Considering the morphological and phylogenetical deviations of *Madsenia* from the above families, as well as the unique M1 + M2 complex in the type species *M. indomita*, we regard *Madsenia* as *incertae sedis* in the order Philasterida (Table 3) and may represent a novel-family level taxon.

### Comments on the taxonomic assignment of *Pseudocyclidium longum*

The order Pleuronematida is mainly characterized by having an expansive oral region with prominent paroral cilia forming a curtain or velum as the organism filter feeds (Lynn 2008). After treating thigmotrichids as a subgroup of Pleuronematida and transferring the former “*Cyclidium porcatum*” to the newly established *Anaerocyclidium* (Gao et al. 2016; Poláková et al. 2023), the monophyly of Pleuronematida has been confirmed as more ribosomal RNA gene sequences were used in phylogenetic analyses (Antipa et al. 2016; Gao et al. 2013; Liu et al. 2022; Lynn and Strüder-Kypke 2005; Zhang and Vďačný 2023; Zhang et al. 2019). However, the discovery of *Pseudocyclidium longum* challenged the monophyly of Pleuronematida since *P. longum* clusters outside of Philasterida + Pleuronematida clade rather than within Pleuronematida (McGowan et al. 2023; Poláková et al. 2021, 2023).

*Pseudocyclidium* Small and Lynn, 1985 is characterized by having three distinct linear oral membranelles, with the first one longer than the second, and both longer than the third. *Pseudocyclidium longum*, which is parasitic in the marine mollusc *Cyclina sinensis*, was originally reported by Xu and Song (1998) with the following characteristics: cell about 55–80 × 15–25 μm in vivo, slender and elongated with a small apical plate; oral region posteriorly extended to mid-portion of cell; single caudal cilium; dikinetids in the anterior three-quarters of somatic kineties, monokinetids in the rest. However, it differs from the type species *P. marylandi* in cell shape (elongated spindle-shaped with both ends narrowed in *P. longum* vs. basically oval in *P. marylandi*), oral length (two-fifths of cell length in *P. longum* vs. more than half of cell length in *P. marylandi*), and the structure of the oral membranelles (short, forming a small patch, oral cilia not prominent in *P. longum* vs. linear, oral cilia prominent forming a sail-like structure in *P. marylandi*) (Small and Lynn 1985; Xu and Song 1998). Therefore, “*Pseudocyclidium longum*” is very likely misidentified and is not a member of *Pseudocyclidium*. The sole sequence of “*P. longum*” (JQ956551) was originally reported in a doctoral dissertation without corresponding morphological information or vouchered specimens, thus the systematic position of *Pseudocyclidium* in the phylogenetic trees is still in doubt. Considering the inconsistency in morphology and the absence of well-identified sequences, we regard *Pseudocyclidium longum* as *incertae sedis* in the subclass Scuticociliatia.

### Comments on the taxonomic assignment of *Protophyra*

*Protophyra* Kofoid, 1903, a monotypic genus, was originally classified as belonging to the subfamily Protophryinae, family Hemispeiridae, with the description of *P. ovicola* which was found in the brood sac of the sea snail *Littorina saxatilis* (Kofoid 1903). In the present SSU rRNA gene trees, *Protophyra* clusters in the clade that includes Hemispeiridae, Ancistridae, Thigmophryidae and Cyclidiidae, and is most closely related to *Myxophyllum* (Thigmophryidae) (Figs. 3, 6), which is consistent with previous studies (Zhang and Vďačný 2021, 2023, 2024). However, the morphological features of *Protophyra* do not precisely fit the definitions of any of these families, resulting in the confusion of its classification.

*Protophyra ovicola* is characterized by an ovoid and strongly laterally flattened cell shape, a narrowed oral region that occupies about three-quarters of the cell length, and a prominently posteriorly positioned cytostome that is located in a depression with conspicuous oral cilia (Fenchel 1965; Raabe 1970). The description of its oral apparatus is, however, incomplete. Observations of silver-stained specimens revealed that it has a prominent paroral membrane and a linear oral M2, both of which are strongly curved at their posterior end (Raabe 1970). In addition, a thigmotactic area is presented in the left-anterior region of the cell and is not separated from the somatic kineties (Fenchel 1965). Based on these features, *Protophyra* can be clearly distinguished from Cyclidiidae in cell shape (ovoid to elongated ovoid, not strongly flattened laterally in Cyclidiidae), oral structure (membranelles often highly fragmented, M2 not curved at posterior end in Cyclidiidae), and lifestyle (marine endocommensal vs. free-living in Cyclidiidae) (Fenchel 1965; Lynn 2008; Raabe 1970).

*Protophyra* was assigned to Ancistridae by Lynn (2008), although in *Protophyra* the cell is strongly laterally-flattened (vs. generally cylindrical to slightly flattened in most other ancistrids) (Fenchel 1965; Raabe 1970). *Protophyra* also resembles two other families, namely Thigmophryidae and Hemispeiridae, in the ovoid cell outline and posteriorly positioned cytostome. However, it differs from Thigmophryidae in the oral apparatus (conspicuous in *Protophyra* vs. reduced and inconspicuous with a single oral membranelle in Thigmophryidae) (Fenchel 1965; Lynn 2008; Raabe 1970). Hemispeiridae has a M2 that is hook-like to strongly curved at the posterior end, which is similar to *Protophyra*. Hemispeiridae, however, often has reduced oral ciliature (vs. conspicuous in *Protophyra*) and a distinct thigmotactic area with reduced dorsal kineties enclosed in a secant system (vs. kineties not reduced, thigmotactic area not separated from somatic kineties in *Protophyra*) (Fenchel 1965; Lynn 2008; Raabe 1970). Combining these comparisons, *Protophyra* better fits the definitions of Ancistridae, so we retain *Protophyra* in the family Ancistridae in contrast with a previous proposal (Zhang and Vďačný 2023).

The morphological features of *Protophrya* are inconsistent with the results inferred by phylogenetic analysis in the present and previous studies (Zhang and Vďačný 2021, 2023, 2024), i.e., the sequence named “*Protophyra ovicola*” (JQ956552) is separated from other Ancistridae sequences and clusters with *Myxophyllum steenstrupi* (family Thigmophryidae) in SSU rRNA trees. The sequence “*P. ovicola* JQ956552” was submitted to GenBank with no vouchered specimens and no corresponding morphological information either in its original record (a doctoral dissertation) or subsequent research articles. Considering the high morphological similarity between *Protophyra ovicola* and *Myxophyllum steenstrupi* (ovoid and strongly laterally flattened cell shape, prominently posteriorly-positioned cytostome located in a depression, and endocommensal lifestyle) (Fenchel 1965; Raabe 1970; Zhang and Vďačný 2021), the misidentification of the sequence “*Protophyra ovicola* JQ956552” cannot be excluded. That is, this sequence may have been extracted from a *Myxophyllum* species, suggesting further studies with more morphological and molecular data are still needed.

## Conclusions

Here, we reconsidered the systematic relationships within the subclass Scuticociliatia according to both morphological and molecular information. Phylogenetic analyses with and without environmental sequences, oral structures, and secondary structures of ITS2 are provided, thereby increasing our understanding of various long-term unresolved systematic relationships within Scuticociliatia. The main conclusions are as follows:

All scuticociliates are assigned to two well-defined clades and one poorly defined group, representing Philasterida, Pleuronematida, and Loxocephalida, respectively.

1. *Homalogastra* differs from all the families in Philasterida and represents a new family, Homalogastridae fam. nov.
2. *Parauronema* is formally transferred to Uronematidae, *Potomacus* is treated as *incertae sedis* in the order Philasterida, and Parauronematidae is considered as a junior synonym of Uronematidae.
3. *Madsenia*, *Parauronema longum*, and *Pseudocyclidium longum* are regarded as *incertae sedis* in the order Philasterida (for the former two) and the subclass Scuticociliatia (for *Pseudocyclidium longum*).
4. *Protophyra* is retained in the family Ancistridae, pending the availability of reliable sequence data.
5. After these adjustments, the order Philasterida contains 15 families: Citrithrixidae, Cohnilembidae, Cryptochilidae, Entodiscidae, Entorhipidiidae, Glauconematidae, Homalogastridae, Orchitophryidae, Paralembidae, Philasteridae, Pseudocohnilembidae, Schizocaryidae, Thyrophylacidae, Uronematidae, and Urozonidae.

## Supplementary Information

Below is the link to the electronic supplementary material.

Supplementary file1 (PDF 3807 KB)

## Data Availability

All data generated or analyzed during this study can be found in online repositories. The names of the repositories and accession numbers can be found at: https://www.ncbi.nlm.nih.gov/genbank/.
