# Supplementary material for: Linking multi-gene and morphological data in the subclass Scuticociliatia (Protista, Ciliophora) with establishment of the new family Homalogastridae fam. nov
Source: Mar Life Sci Technol. 2024 Dec 19;7(1):1–22. doi: 10.1007/s42995-024-00264-8 (PMC11871206; doi:10.1007/s42995-024-00264-8)
Supplement: Supplementary file 1 — Supplementary file1 (PDF 3807 KB) [file 42995_2024_264_MOESM1_ESM.pdf]

Supplementary file

Linking multi-gene and morphologic data in the subclass Scuticociliatia (Protista, Ciliophora) with establishment of the new family Homalogastridae fam. nov.

Mingjian Liu[1,2,*], Limin Jiang[1,*], Zhe Zhang[1,*], Fan Wei[1,*], Honggang Ma[1], Zigui Chen[1], Khaled A.S. Al-Rasheid[3], Hunter N. Hines[4], Chundi Wang[5,**]

[1] *Key Laboratory of Evolution & Marine Biodiversity (Ministry of Education), and Institute of Evolution & Marine Biodiversity, Ocean University of China, Qingdao 266003, China*

[2] *College of Marine Life Sciences, Ocean University of China, Qingdao 266003, China*

[3] *Zoology Department, College of Science, King Saud University, Riyadh 11451, Saudi Arabia*

[4] *Harbor Branch Oceanographic Institute, Florida Atlantic University, Fort Pierce, FL 34946, USA*

[5] *Laboratory of Marine Protozoan Biodiversity & Evolution, Marine College, Shandong University, Weihai 264209, China*

*These authors contributed equally to this work.

**Corresponding author: Laboratory of Marine Protozoan Biodiversity & Evolution, Marine College, Shandong University. E-mail address: wangcd@sdu.edu.cn (C. Wang).

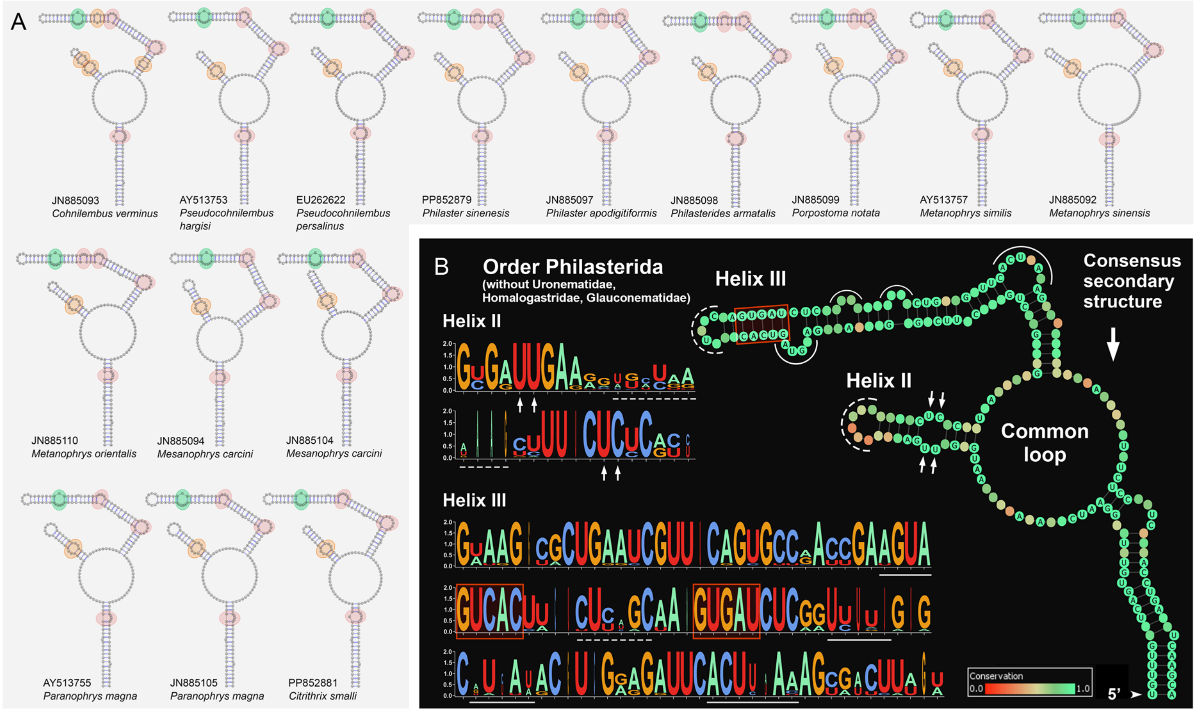

Supplementary Fig. S1 Comparison of putative ITS2 region secondary structure of the taxa from the order Philasterida except for Uronematidae, Homalogastridae, Glauconematidae (A) and the consensus secondary structure based on these sequences, with nucleotides composition in different helixes (B). Colored circles in A indicate bulges with at least two nucleotides. Yellow circles represent bulges with same number of nucleotides at both sides, green circles represent bulges with more nucleotides at 5' ends than 3' ends of ITS2 region, and pink circles represent bulges with more nucleotides at 3' ends than 5' ends of ITS2 region. Arrows in B indicate pyrimidine-pyrimidine mismatch of Helix II, box indicates the highly conserved motif in Helix III, dash lines indicate terminal bulges in helixes, and solid lines indicate bulges in helixes with more than one nucleotide. Fully conserved positions of the consensus structure are marked with specific nucleotides.

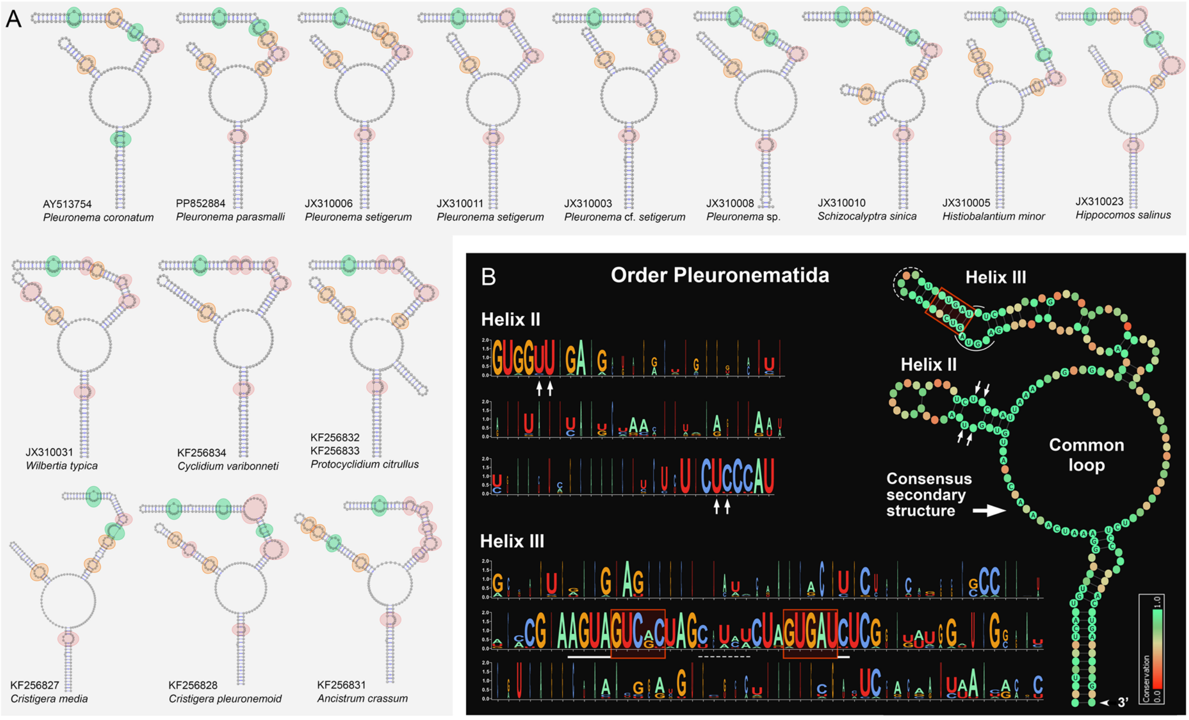

Supplementary Fig. S2 Comparison of putative ITS2 region secondary structure of the taxa from the order Pleuronematida (A) and the consensus secondary structure based on these selected sequences, with nucleotides composition in different helixes (B). Colored circles in A indicate bulges with at least two nucleotides. Yellow circles represent bulges with same number of nucleotides at both sides, green circles represent bulges with more nucleotides at 5' ends than 3' ends of ITS2 region, and pink circles represent bulges with more nucleotides at 3' ends than 5' ends of ITS2 region. Arrows in B indicate pyrimidine-pyrimidine mismatch of Helix II, box indicates the relatively conserved motif in Helix III, dash lines indicate terminal bulges in helixes, and solid lines indicate bulges in helixes with more than one nucleotide. Fully conserved positions of the consensus structure are marked with specific nucleotides.

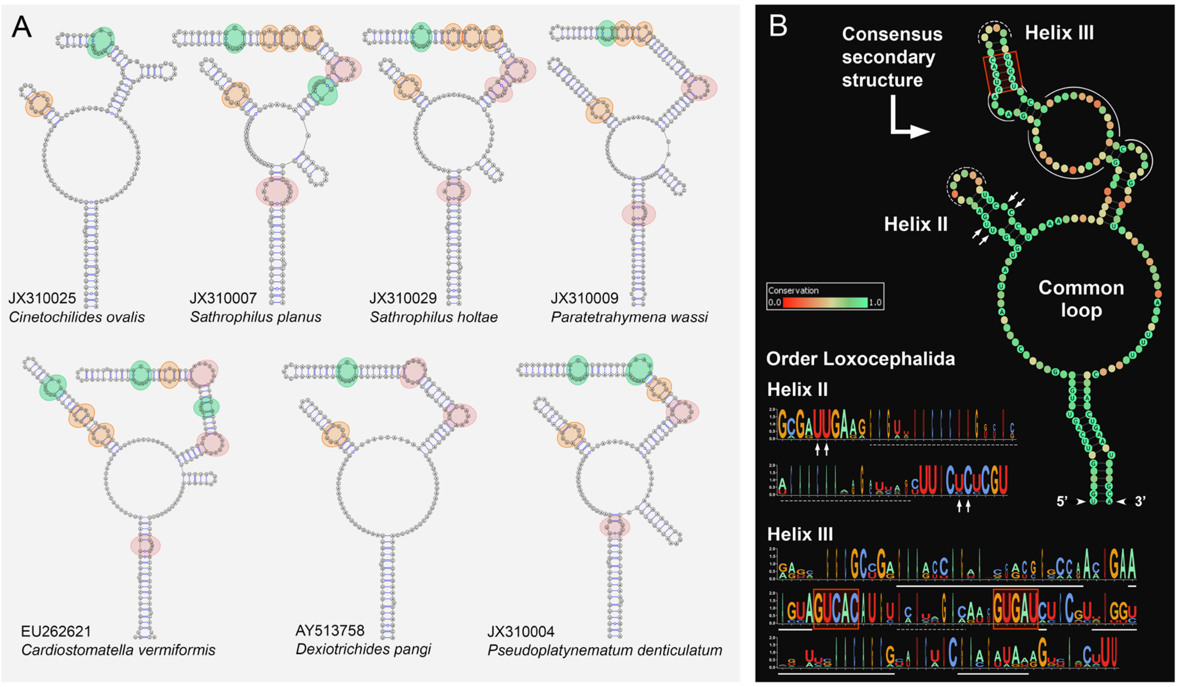

Supplementary Fig. S3 Comparison of putative ITS2 region secondary structure of the taxa from the order Loxocephalida (A) and the consensus secondary structure based on these selected sequences, with nucleotides composition in different helixes (B). Colored circles in A indicate bulges with at least two nucleotides. Yellow circles represent bulges with same number of nucleotides at both sides, green circles represent bulges with more nucleotides at 5' ends than 3' ends of ITS2 region, and pink circles represent bulges with more nucleotides at 3' ends than 5' ends of ITS2 region. Arrows in B indicate pyrimidine-pyrimidine mismatch of Helix II, box indicates the highly conserved motif in Helix III, dash lines indicate terminal bulges in helixes, and solid lines indicate bulges in helixes with more than one nucleotide. Fully conserved positions of the consensus structure are marked with specific nucleotides.

Supplementary Table S1 Primers used for PCR amplification in the present study

Genes	Primers	Sequences (5′–3′)	Reference
ITS1-5.8S-ITS2 region	5.8SF	GTAGGTGAACCTGCGGAAGGATCATTA	Yi et al. (2009)
	5.8SR	TACTGATATGCTTAAGTTCAGCGG	Yi et al. (2009)
LSU rRNA gene	28S-F2	AC(C/G)CGCTG(A/G)A(T/C)TTAAGCAT	Moreira et al. (2007)
	28S-R2	AACCTTGGAGACCTGAT	Moreira et al. (2007)
	28S-F3	ACCCGCTGAACTTAAGCAT	Moreira et al. (2007)
	28S-R3	CATTCGGCAGGTGAGTTGTTACAC	Gong et al. (2007)
COI gene	F298dT-S	TGTAAAACGACGGCCAGTGCNCAYGGTYTAATNATGGT	Strüder-Kypke and Lynn (2010)
	R1184dT-S	CAGGAAACAGCTATGACTADACYTCAGGGTGACCRAAAAATCA	Strüder-Kypke and Lynn (2010)
	COI-NEW-17-F1	TGGTNTTTTTTGTWGTWGTDCC	Zhang et al. (2019)
	COI-NEW-812-R2	GTWGTTTGTCAATGHCKRTCTA	Zhang et al. (2019)

Supplementary Table S2 GenBank accession numbers of the sequences used in the present work

Subclasses	Species/Populations	SSU rRNA gene	ITS-5.8S-ITS2 region	LSU rRNA gene	*COI* gene
ASTOMATIA	*Almophrya bivacuolata*	HQ446281	-	-	-
SCUTICOCILIATIA	***Ancistrum crassum*** **pop. 1**	HM236340	KF256831	KF256825	MH605544
SCUTICOCILIATIA	*Ancistrum crassum* pop. 2	HQ445964	-	-	-
SCUTICOCILIATIA	*Ancistrum crassum* pop. 3	JQ956537	-	-	-
SCUTICOCILIATIA	*Ancistrum crassum* pop. 4	JQ956538	-	-	-
SCUTICOCILIATIA	*Ancistrum crassum* pop. 5	JQ956539	-	-	-
SCUTICOCILIATIA	*Ancistrum crassum* pop. 6	MF407351	-	-	-
SCUTICOCILIATIA	*Ancistrum japonicum*	JQ956536	-	-	-
SCUTICOCILIATIA	*Ancistrum mytili*	JQ956535	-	-	-
SCUTICOCILIATIA	*Ancistrum* sp.	HQ445963	-	-	-
SCUTICOCILIATIA	*Anophyroides haemophila*	U51554	-	-	-
ASTOMATIA	***Anoplophrya lumbrici***	MN121061	MN897871	MN897871	-
ASTOMATIA	***Anoplophrya vulgaris***	MN121065	MN897875	MN897875	-
SCUTICOCILIATIA	*Apouronema harbinensis*	MN524103	-	-	-
SCUTICOCILIATIA	*Biggaria bermudensis*	JQ956541	-	-	-
SCUTICOCILIATIA	*Boveria labialis*	MF407350	-	-	-
SCUTICOCILIATIA	***Boveria subcylindrica*** **pop. 1**	FJ848878	-	KF256826	-
SCUTICOCILIATIA	*Boveria subcylindrica* pop. 2	HQ445965	-	-	-
PERITRICHIA	***Carchesium polypinum***	HM852991	FJ810354	FJ810354	FJ810312
SCUTICOCILIATIA	***Cardiostomatella vermiformis***	AY881632	EU262621	JX310024	MH605533
SCUTICOCILIATIA	***Cinetochilides ovalis***	FJ870103	JX310025	JX310025	MH605540
SCUTICOCILIATIA	***Citrithrix smalli***	MT982807	**PP852881**	**PP784539**	**PQ256152**
SCUTICOCILIATIA	***Cohnilembus verminus*** **pop. 1**	HM236339	JN885093	JN885111	MH605563
SCUTICOCILIATIA	*Cohnilembus verminus* pop. 2	Z22878	-	-	-
COLPODEA	***Colpoda lucida***	EU039895	-	-	FJ905159
COLPODEA	***Colpoda magna***	EU039896	-	-	FJ905160
COLPODEA	***Colpoda inflata***	KM222106	KM222071	KM222160	-
SCUTICOCILIATIA	*Conchophthirus cucumis*	JQ956543	-	-	-
SCUTICOCILIATIA	*Conchophthirus curtus*	MN704274	-	-	-
SCUTICOCILIATIA	*Conchophthirus lamellidens*	JQ956542	-	-	-
SCUTICOCILIATIA	***Conchophthirus*** **sp. 1**	MN704275	-	-	MN702825
SCUTICOCILIATIA	*Conchophthirus* sp. 2	MN704276	-	-	-
SCUTICOCILIATIA	*Conchophthirus* sp. 3	MN704277	-	-	-
SCUTICOCILIATIA	*Conchophthirus* sp. 4	MN704278	-	-	-
SCUTICOCILIATIA	***Cristigera media***	FJ868180	KF256827	KF256827	MH605569
SCUTICOCILIATIA	***Cristigera pleuronemoides***	KF256816	KF256828	KF256828	MH605546
SCUTICOCILIATIA	*Cyclidium glaucoma* pop. 1	EU032356	-	-	-
SCUTICOCILIATIA	*Cyclidium glaucoma* pop. 2	KY476313	-	-	-
SCUTICOCILIATIA	*Cyclidium glaucoma* pop. 3	KY886366	-	-	-
SCUTICOCILIATIA	*Cyclidium glaucoma* pop. 4	Z22879	-	-	-
SCUTICOCILIATIA	*Cyclidium glaucoma* pop. 5	-	-	-	FJ905125
SCUTICOCILIATIA	*Cyclidium marinum* pop. 1	JQ956553	-	-	-
SCUTICOCILIATIA	*Cyclidium marinum* pop. 2	KY886367	-	-	-
SCUTICOCILIATIA	*Cyclidium porcatum*	Z29517	-	-	-
SCUTICOCILIATIA	*Cyclidium sinicum*	KX853100	-	-	-
SCUTICOCILIATIA	*Cyclidium* sp.	LC497866	-	-	-
SCUTICOCILIATIA	***Cyclidium varibonneti***	KF256817	KF256834	KF256830	MH605558
SCUTICOCILIATIA	*Cyclidium vorax*	MN524102	-	-	-
SCUTICOCILIATIA	*Dexiotricha* cf. *granulosa*	KF878931	-	-	-
SCUTICOCILIATIA	*Dexiotricha colpidiopsis*	MG819725	-	-	-
SCUTICOCILIATIA	***Dexiotricha elliptica***	KF878932	-	-	MH605568
SCUTICOCILIATIA	*Dexiotricha* sp. 1	JQ723963	-	-	-
SCUTICOCILIATIA	*Dexiotricha* sp. 2	MK354012	-	-	-
SCUTICOCILIATIA	*Dexiotricha* sp. 3	MN704273	-	-	-
SCUTICOCILIATIA	*Dexiotricha* sp. 4	-	-	-	FJ905124
SCUTICOCILIATIA	***Dexiotrichides pangi***	AY212805	AY513758	-	-
SCUTICOCILIATIA	***Entodiscus borealis***	AY541687	-	-	FJ905123
SCUTICOCILIATIA	*Entorhipidium enchi*	JQ956545	-	-	-
SCUTICOCILIATIA	*Entorhipidium pilatum*	AY541689	-	-	-
SCUTICOCILIATIA	*Entorhipidium tenue* pop. 1	AY541688	-	-	-
SCUTICOCILIATIA	*Entorhipidium tenue* pop. 2	JQ956544	-	-	-
SCUTICOCILIATIA	*Entorhipidium triangularis*	AY541690	-	-	-
SCUTICOCILIATIA	*Entorhipidium* sp.	-	-	-	FJ905122
PERITRICHIA	***Epicarchesium abrae***	DQ190462	EU340854	-	-
SCUTICOCILIATIA	*Eurystomatella sinica* pop. 1	FJ012143	-	-	-
SCUTICOCILIATIA	***Eurystomatella sinica*** **pop. 2**	JX310021	-	-	MH605566
SCUTICOCILIATIA	*Falcicyclidium atractodes*	FJ868182	-	-	-
SCUTICOCILIATIA	*Falcicyclidium citriforme*	-	-	-	MH605550
SCUTICOCILIATIA	***Falcicyclidium fangi*** **pop. 1**	FJ868183	-	-	MH605538
SCUTICOCILIATIA	*Falcicyclidium fangi* pop. 2	FJ868184	-	-	-

SCUTICOCILIATIA	*Falcicyclidium fangi* pop. 3	FJ868185	-	-	-
SCUTICOCILIATIA	*Falcicyclidium plouneouri* pop. 1	FJ868181	-	-	-
SCUTICOCILIATIA	*Falcicyclidium plouneouri* pop. 2	KF256818	-	-	-
SCUTICOCILIATIA	*Falcicyclidium plouneouri* pop. 3	KF256819	-	-	-
SCUTICOCILIATIA	*Falcicyclidium plouneouri* pop. 4	U27816	-	-	-
PENICULIA	***Frontonia magna***	FJ876953	KJ475219	KJ475278	KJ475340
PENICULIA	*Frontonia magna*	-	-	-	KJ475343
SCUTICOCILIATIA	***Glauconema trihymene***	GQ214552	HM099917	-	-
ASTOMATIA	*Haptophrya planariarum* pop. 1	MH035978	-	-	-
ASTOMATIA	*Haptophrya planariarum* pop. 2	MK454737	-	-	-
ASTOMATIA	*Haptophrya planariarum* pop. 3	MK454738	-	-	-
ASTOMATIA	*Haptophrya planariarum* pop. 4	MK454740	-	-	-
ASTOMATIA	*Haptophrya planariarum* pop. 5	MK454741	-	-	-
ASTOMATIA	*Haptophrya planariarum* pop. 6	MK454742	-	-	-
ASTOMATIA	*Haptophrya planariarum* pop. 7	MK454743	-	-	-
ASTOMATIA	*Haptophrya planariarum* pop. 8	MK454744	-	-	-
ASTOMATIA	*Haptophrya planariarum* pop. 9	MK454746	-	-	-
ASTOMATIA	*Haptophrya planariarum* pop. 10	MK454747	-	-	-
SCUTICOCILIATIA	***Hippocomos salinus***	JX310012	JX310023	JX310023	MH605543
SCUTICOCILIATIA	*Histiobalantium bodamicum*	MT886454	-	-	-
SCUTICOCILIATIA	***Histiobalantium comosa***	KU665372	-	-	KU665391
SCUTICOCILIATIA	***Histiobalantium minor***	JX310013	JX310005	-	MH605548
SCUTICOCILIATIA	*Histiobalantium natans*	AB450957	-	-	-
SCUTICOCILIATIA	***Homalogastra similis***	MT982808	-	**PP784540**	-
SCUTICOCILIATIA	***Homalogastra parasetosa***	MG581969	MN727050	-	**PQ256155**
SCUTICOCILIATIA	*Homalogastra setosa* pop. 1	EF158847	-	-	-
SCUTICOCILIATIA	*Homalogastra setosa* pop. 2	EF158848	-	-	-
SCUTICOCILIATIA	*Homalogastra setosa* pop. 3	-	EF158844	-	-
SCUTICOCILIATIA	*Homalogastra setosa* pop. 4	GU590870	-	-	-
APOSTOMATIA	*Hyalophysa bradburyae*	MN537438	-	-	-
APOSTOMATIA	*Hyalophysa lwoffi*	EU503538	-	-	-
HYMENOSTOMATIA	***Ichthyophthirius multifiliis***	U17354	-	-	GU439201
SCUTICOCILIATIA	*Madsenia indomita*	JQ956550	-	-	-
SCUTICOCILIATIA	*Madsenia* sp.	MT271859	-	-	-
COLPODEA	***Maryna umbrellata***	JF747217	-	-	FJ905161
SCUTICOCILIATIA	***Mesanophrys carcini* pop. 1**	AY103189	AY513756	-	-
SCUTICOCILIATIA	***Mesanophrys carcini* pop. 2**	JN885085	JN885094	JN885112	MH605547
SCUTICOCILIATIA	***Mesanophrys carcini* pop. 3**	JN885086	JN885104	JN885113	MH605556
SCUTICOCILIATIA	*Mesanophrys* sp. 1	FJ936002	-	-	-
SCUTICOCILIATIA	*Mesanophrys* sp. 2	MN260367	-	-	-
APOSTOMATIA	***Metacollinia luciensis***	MH200618	-	-	MH182620
SCUTICOCILIATIA	***Metanophrys orientalis***	JN885084	JN885110	JN885129	MH605551
SCUTICOCILIATIA	***Metanophrys similis***	AY314803	AY513757	-	-
SCUTICOCILIATIA	***Metanophrys sinensis***	HM236336	JN885092	JN885114	MH605565
ASTOMATIA	***Metaradiophrya lumbrici* pop. 1**	MN121068	MN897880	MN897880	-
ASTOMATIA	*Metaradiophrya lumbrici* pop. 2	MN121069	-	-	-
ASTOMATIA	*Metaradiophrya lumbrici* pop. 3	MN121070	-	-	-
ASTOMATIA	*Metaradiophrya lumbrici* pop. 4	MN121071	-	-	-
ASTOMATIA	*Metaradiophrya lumbrici* pop. 5	MN121072	-	-	-
ASTOMATIA	*Metaradiophrya lumbrici* pop. 6	MN121073	-	-	-
ASTOMATIA	*Metaradiophrya lumbrici* pop. 7	MN121074	-	-	-
ASTOMATIA	*Metaradiophrya lumbrici* pop. 8	MN121075	-	-	-
ASTOMATIA	*Metaradiophrya* sp.	HQ446279	-	-	-
ASTOMATIA	***Metaradiophrya varians* pop. 1**	MN121076	MN897886	MN897886	-
ASTOMATIA	*Metaradiophrya varians* pop. 2	MN121077	-	-	-
ASTOMATIA	*Metaradiophrya varians* pop. 3	MN121078	-	-	-
ASTOMATIA	*Metaradiophrya varians* pop. 4	MN121079	-	-	-
SCUTICOCILIATIA	*Miamiensis avidus* pop. 1	AY550080	-	-	-
SCUTICOCILIATIA	*Miamiensis avidus* pop. 2	AY642280	-	-	-
SCUTICOCILIATIA	***Miamiensis avidus* pop. 3**	EU831192	-	-	EU831213
SCUTICOCILIATIA	***Miamiensis avidus* pop. 4**	EU831193	-	-	EU831214
SCUTICOCILIATIA	*Miamiensis avidus* pop. 5	EU831194	-	-	-
SCUTICOCILIATIA	***Miamiensis avidus* pop. 6**	EU831195	-	-	EU831216
SCUTICOCILIATIA	*Miamiensis avidus* pop. 7	EU831196	-	-	-
SCUTICOCILIATIA	*Miamiensis avidus* pop. 8	EU831198	-	-	-
SCUTICOCILIATIA	*Miamiensis avidus* pop. 9	JN689229	-	-	-
SCUTICOCILIATIA	*Miamiensis avidus* pop. 10	JN689230	-	-	-
SCUTICOCILIATIA	***Miamiensis avidus* pop. 11**	JN885091	JN885095	JN885115	MH605562
SCUTICOCILIATIA	*Miamiensis avidus* pop. 12	KU720304	-	-	-
SCUTICOCILIATIA	*Miamiensis avidus* pop. 13	KU992658	-	-	-
SCUTICOCILIATIA	***Miamiensis avidus* pop. 14**	KX259260	-	-	KX259258
SCUTICOCILIATIA	*Miamiensis avidus* pop. 15	KX357144	-	-	-
SCUTICOCILIATIA	*Miamiensis avidus* pop. 16	KY082893	-	-	-
SCUTICOCILIATIA	*Miamiensis avidus* pop. 17	MN611447	-	-	-

SCUTICOCILIATIA	*Miamiensis avidus* pop. 18	MN611448	-	-	-
SCUTICOCILIATIA	*Miamiensis avidus* pop. 19	-	-	-	EU831218
SCUTICOCILIATIA	*Miamiensis avidus* pop. 20	-	-	-	EU831226
SCUTICOCILIATIA	*Miamiensis avidus* pop. 21	-	-	-	EU831227
SCUTICOCILIATIA	*Miamiensis avidus* pop. 22	-	-	-	EU831233
SCUTICOCILIATIA	*Miamiensis avidus* pop. 23	-	-	-	GQ855300
SCUTICOCILIATIA	*Miamiensis avidus* pop. 24	-	-	-	MH078246
SCUTICOCILIATIA	*Miamiensis avidus* pop. 25	-	-	-	MH078247
SCUTICOCILIATIA	*Miamiensis avidus* pop. 26	-	-	-	MH078249
SCUTICOCILIATIA	*Miamiensis* sp.	FJ936000	-	-	-
SCUTICOCILIATIA	*Mytilophilus pacificae* pop. 1	JQ956546	-	-	-
SCUTICOCILIATIA	*Mytilophilus pacificae* pop. 2	KU665346	-	-	-
SCUTICOCILIATIA	***Mytilophilus pacificae* pop. 3**	KU665347	-	-	KU665375
SCUTICOCILIATIA	*Mytilophilus pacificae* pop. 4	KU665348	-	-	-
SCUTICOCILIATIA	*Mytilophilus pacificae* pop. 5	KU665349	-	-	-
SCUTICOCILIATIA	*Mytilophilus pacificae* pop. 6	KU665350	-	-	-
SCUTICOCILIATIA	*Mytilophilus pacificae* pop. 7	KU665351	-	-	-
SCUTICOCILIATIA	*Mytilophilus pacificae* pop. 8	KU665353	-	-	-
SCUTICOCILIATIA	***Mytilophilus pacificae* pop. 9**	KU665354	-	-	KU665374
SCUTICOCILIATIA	***Myxophyllum steenstrupi* pop. 1**	MT649635	MT649641	MT649641	MT648665
SCUTICOCILIATIA	***Myxophyllum steenstrupi* pop. 2**	MT649636	MT649642	MT649642	MT648666
SCUTICOCILIATIA	***Myxophyllum steenstrupi* pop. 3**	MT649637	MT649643	MT649643	MT648667
SCUTICOCILIATIA	***Myxophyllum steenstrupi* pop. 4**	MT649638	MT649644	MT649644	MT648668
SCUTICOCILIATIA	***Myxophyllum steenstrupi* pop. 5**	MT649639	MT649645	MT649645	MT648669
SCUTICOCILIATIA	***Myxophyllum steenstrupi* pop. 6**	MT649640	MT649646	MT649646	MT648670
ASTOMATIA	*Njinella prolifera*	HQ446276	-	-	-
HYMENOSTOMATIA	***Ophryoglena catenula***	U17355	-	-	FJ905118
ASTOMATIA	*Paraclausilocola constricta*	HQ446275	-	-	-
ASTOMATIA	*Paraclausilocola elongata*	HQ446274	-	-	-
SCUTICOCILIATIA	*Paralembus digitiformis*	JQ956549	-	-	-
PENICULIA	***Paramecium biaurelia***	KU729877	-	-	DQ912534
PENICULIA	***Paramecium caudatum***	KX302699	-	-	FN424190
PENICULIA	***Paramecium jenningsi***	HE662760	-	-	FJ905139
PENICULIA	***Paramecium primaurelia***	AF100315	-	-	FJ905141
PENICULIA	*Paramecium sexaurelia*	-	-	-	FJ905154
PENICULIA	***Paramecium tetraurelia***	X03772	-	-	DQ912542
SCUTICOCILIATIA	***Paramesanophrys typica***	MH574792	-	**PP784541**	-
SCUTICOCILIATIA	***Paranophrys magna* pop. 1**	AY103191	AY513755	-	-
SCUTICOCILIATIA	***Paranophrys magna* pop. 2**	JN885089	JN885105	JN885116	-
SCUTICOCILIATIA	*Paranophrys magna* pop. 3	JQ956548	-	-	-
SCUTICOCILIATIA	*Paranophrys magna* pop. 4	FJ858379	-	-	-
SCUTICOCILIATIA	*Paratetrahymena parawassi*	FJ876969	-	-	-
SCUTICOCILIATIA	*Paratetrahymena* sp.	EU744176	-	-	-
SCUTICOCILIATIA	***Paratetrahymena wassi* pop. 1**	GQ292767	-	JX310027	-
SCUTICOCILIATIA	***Paratetrahymena wassi* pop. 2**	JX310019	JX310009	JX310026	-
SCUTICOCILIATIA	*Parauronema* cf. *virginianum* pop. 1	FJ595488	-	-	-
SCUTICOCILIATIA	***Parauronema* cf. *virginianum* pop. 2**	JN885082	JN885106	JN885117	-
SCUTICOCILIATIA	***Parauronema longum* pop. 1**	AY212807	AY513759	-	-
SCUTICOCILIATIA	***Parauronema longum* pop. 2**	HM236338	JN885096	JN885118	MH605542
SCUTICOCILIATIA	*Parauronema virginianum* pop. 1	AY392128	-	-	-
SCUTICOCILIATIA	***Parauronema virginianum* pop. 2**	JN885087	JN885109	JN885128	-
SCUTICOCILIATIA	***Peniculistoma mytili* pop. 1**	KU665355	-	-	KU665390
SCUTICOCILIATIA	***Peniculistoma mytili* pop. 2**	KU665356	-	-	KU665389
SCUTICOCILIATIA	*Peniculistoma mytili* pop. 3	KU665357	-	-	-
SCUTICOCILIATIA	*Peniculistoma mytili* pop. 4	KU665358	-	-	-
SCUTICOCILIATIA	***Peniculistoma mytili* pop. 5**	KU665359	-	-	KU665387
SCUTICOCILIATIA	***Peniculistoma mytili* pop. 6**	KU665360	-	-	KU665388
SCUTICOCILIATIA	***Peniculistoma mytili* pop. 7**	KU665361	-	-	KU665382
SCUTICOCILIATIA	***Peniculistoma mytili* pop. 8**	KU665362	-	-	KU665380
SCUTICOCILIATIA	*Peniculistoma mytili* pop. 9	KU665363	-	-	-
SCUTICOCILIATIA	*Peniculistoma mytili* pop. 10	KU665364	-	-	-
SCUTICOCILIATIA	*Peniculistoma mytili* pop. 11	KU665365	-	-	-
SCUTICOCILIATIA	*Peniculistoma mytili* pop. 12	KU665366	-	-	-
SCUTICOCILIATIA	*Peniculistoma mytili* pop. 13	KU665367	-	-	-
SCUTICOCILIATIA	***Peniculistoma mytili* pop. 14**	KU665368	-	-	KU665386
SCUTICOCILIATIA	***Peniculistoma mytili* pop. 15**	KU665369	-	-	KU665384
SCUTICOCILIATIA	***Peniculistoma mytili* pop. 16**	KU665370	-	-	KU665385
SCUTICOCILIATIA	***Peniculistoma mytili* pop. 17**	KU665371	-	-	KU665383
SCUTICOCILIATIA	*Peniculistoma mytili* pop. 18	-	-	-	KU665381
SCUTICOCILIATIA	***Philaster apodigitiformis***	FJ648350	JN885097	JN885119	MH605532
SCUTICOCILIATIA	***Philaster sinensis***	KJ815049	**PP852879**	**PP784536**	MH605552
SCUTICOCILIATIA	***Philasterides armatalis***	FJ848877	JN885098	JN885120	MH605534
SCUTICOCILIATIA	*Philasterides dicentrarchi* pop. 1	GU572375	-	-	-
SCUTICOCILIATIA	*Philasterides dicentrarchi* pop. 2	JX914665	-	-	-

SCUTICOCILIATIA	*Philasterides dicentrarchi* pop. 3	MK002746	-	-	-
SCUTICOCILIATIA	***Plagiopyliella pacifica***	AY541685	-	-	FJ905121
SCUTICOCILIATIA	*Platynematum salinarum*	KF301567	-	-	-
COLPODEA	***Platyophrya bromelicola***	EU039905	-	-	FJ905158
SCUTICOCILIATIA	*Pleuronema binucleatum*	KT033424	-	-	-
SCUTICOCILIATIA	***Pleuronema* cf. *setigerum***	FJ848875	JX310003	-	MH605539
SCUTICOCILIATIA	***Pleuronema coronatum* pop. 1**	AY103188	AY513754	-	-
SCUTICOCILIATIA	*Pleuronema coronatum* pop. 2	HM140396	-	-	-
SCUTICOCILIATIA	***Pleuronema coronatum* pop. 3**	JX310014	-	-	MH605531
SCUTICOCILIATIA	*Pleuronema coronatum* pop. 4	JX310018	-	-	-
SCUTICOCILIATIA	*Pleuronema coronatum* pop. 5	-	-	-	MH605561
SCUTICOCILIATIA	*Pleuronema czapikae*	EF486863	-	-	-
SCUTICOCILIATIA	***Pleuronema elegans***	KF840518	-	-	MH605554
SCUTICOCILIATIA	*Pleuronema foissneri*	OL654416	-	-	-
SCUTICOCILIATIA	***Pleuronema grolierei***	KF840519	-	-	MH605549
SCUTICOCILIATIA	*Pleuronema marinum*	KF206428	-	-	-
SCUTICOCILIATIA	*Pleuronema orientale*	KF206429	-	-	-
SCUTICOCILIATIA	*Pleuronema paraorientale*	OL654419	-	-	-
SCUTICOCILIATIA	*Pleuronema parasalmastra*	OL654418	-	-	-
SCUTICOCILIATIA	***Pleuronema parasmalli***	OL654417	**PP852884**	-	**PQ256156**
SCUTICOCILIATIA	*Pleuronema parawiackowskii*	KT033423	-	-	-
SCUTICOCILIATIA	*Pleuronema paucisaetosum*	KF206430	-	-	-
SCUTICOCILIATIA	***Pleuronema puytoraci***	KF840520	-	-	**PQ256154**
SCUTICOCILIATIA	***Pleuronema setigerum* pop. 1**	FJ848874	JX310006	-	MH605537
SCUTICOCILIATIA	***Pleuronema setigerum* pop. 2**	JX310015	JX310011	-	-
SCUTICOCILIATIA	*Pleuronema sinica*	EF486864	-	-	-
SCUTICOCILIATIA	***Pleuronema* sp. 1**	FJ848876	JX310008	-	-
SCUTICOCILIATIA	*Pleuronema* sp. 2	JX310017	-	-	-
SCUTICOCILIATIA	***Pleuronema wiackowskii***	JX310016	-	-	**PQ256153**
SCUTICOCILIATIA	***Porpostoma notata***	HM236335	JN885099	JN885121	-
SCUTICOCILIATIA	***Protocyclidium citrullus* pop. 1**	KF256820	KF256832	KF256823	MH605545
SCUTICOCILIATIA	***Protocyclidium citrullus* pop. 2**	KF256821	KF256833	KF256824	-
SCUTICOCILIATIA	***Protocyclidium sinica***	KF256822	-	KF256829	-
SCUTICOCILIATIA	*Protophyra ovicola*	JQ956552	-	-	-
SCUTICOCILIATIA	***Pseudocohnilembus hargisi* pop. 1**	AY212806	AY513753	-	-
SCUTICOCILIATIA	*Pseudocohnilembus hargisi* pop. 2	AY833087	-	-	-
SCUTICOCILIATIA	***Pseudocohnilembus hargisi* pop. 3**	JN885090	JN885100	JN885122	MH605559
SCUTICOCILIATIA	***Pseudocohnilembus longisetus***	FJ899594	-	-	GQ500580
SCUTICOCILIATIA	*Pseudocohnilembus marinus*	Z22880	-	-	-
SCUTICOCILIATIA	*Pseudocohnilembus persalinus* pop. 1	AY551906	-	-	-
SCUTICOCILIATIA	*Pseudocohnilembus persalinus* pop. 2	AY835669	-	-	-
SCUTICOCILIATIA	***Pseudocohnilembus persalinus* pop. 3**	GQ265955	EU262622	-	-
SCUTICOCILIATIA	*Pseudocohnilembus persalinus* pop. 4	GU584096	-	-	-
SCUTICOCILIATIA	*Pseudocohnilembus persalinus* pop. 5	JQ956554	-	-	-
SCUTICOCILIATIA	*Pseudocohnilembus persalinus* pop. 6	MG452732	-	-	-
SCUTICOCILIATIA	*Pseudocohnilembus persalinus* pop. 7	MG452733	-	-	-
SCUTICOCILIATIA	*Pseudocohnilembus persalinus* pop. 8	MG452734	-	-	-
SCUTICOCILIATIA	*Pseudocohnilembus persalinus* pop. 9	MG452735	-	-	-
SCUTICOCILIATIA	*Pseudocohnilembus persalinus* pop. 10	MT081565	-	-	-
SCUTICOCILIATIA	*Pseudocohnilembus persalinus* pop. 11	-	-	-	GQ500579
APOSTOMATIA	***Pseudocollinia beringensis***	HQ591477	HQ591477	-	HQ591500
APOSTOMATIA	***Pseudocollinia brintoni* pop.1**	HQ591470	-	-	HQ591490
APOSTOMATIA	*Pseudocollinia brintoni* pop.2	-	-	-	HQ591489
APOSTOMATIA	***Pseudocollinia oregonensis***	HQ591473	HQ591473	-	HQ591494
APOSTOMATIA	***Pseudocollinia similis***	HQ591485	HQ591485	-	HQ591492
SCUTICOCILIATIA	*Pseudocyclidium longum*	JQ956551	-	-	-
SCUTICOCILIATIA	***Pseudoplatynematum denticulatum***	JX310020	JX310004	JX310028	-
PERITRICHIA	***Pseudovorticella paracratera***	DQ662847	EU340855	-	-
SCUTICOCILIATIA	***Sathrophilus holtae***	FJ868188	JX310029	JX310029	-
SCUTICOCILIATIA	***Sathrophilus planus***	FJ868186	JX310007	JX310030	-
SCUTICOCILIATIA	***Schizocalyptra aeschtae***	DQ777744	-	-	MH605530
SCUTICOCILIATIA	*Schizocalyptra similis*	EU744177	-	-	-
SCUTICOCILIATIA	***Schizocalyptra sinica***	FJ156106	JX310010	-	-
SCUTICOCILIATIA	***Schizocalyptra* sp.**	FJ848873	-	-	MH605536
SCUTICOCILIATIA	*Schizocaryum dogieli*	AF527756	-	-	-
SCUTICOCILIATIA	*Schizocaryum* sp.	EU597807	-	-	-
ASTOMATIA	***Subanoplophrya nodulata***	MN121063	MN897873	MN897873	-
HYMENOSTOMATIA	*Tetrahymena malaccensis*	-	-	-	DQ927303
HYMENOSTOMATIA	***Tetrahymena paravorax***	EF070253	-	-	DQ927304
HYMENOSTOMATIA	***Tetrahymena pigmentosa***	M26358	-	-	DQ927305
HYMENOSTOMATIA	***Tetrahymena pyriformis***	M98021	-	-	EF070300
SCUTICOCILIATIA	*Thyrophylax vorax*	AY541686	-	-	-
PERITRICHIA	***Trichodina heterodentata***	AY788099	EF569681	-	-
PERITRICHIA	*Trichodinella myakkae*	AY102176	-	-	-

UROCENTRIA	***Urocentrum turbo* pop. 1**	AF255357	-	-	FJ905137
UROCENTRIA	***Urocentrum turbo* pop. 2**	EF114299	EF114293	-	-
UROCENTRIA	*Urocentrum turbo* pop. 3	EF114300	-	-	-
SCUTICOCILIATIA	***Uronema apomarinum***	MG581965	MN727051	**PP784544**	-
SCUTICOCILIATIA	***Uronema elegans***	AY103190	AY513760	-	-
SCUTICOCILIATIA	***Uronema heteromarinum***	FJ870100	JN885101	JN885123	MH605535
SCUTICOCILIATIA	*Uronema marinum* pop. 1	-	GQ259751	-	-
SCUTICOCILIATIA	*Uronema marinum* pop. 2	-	GQ259752	-	-
SCUTICOCILIATIA	*Uronema marinum* pop. 3	-	GQ259753	-	-
SCUTICOCILIATIA	*Uronema marinum* pop. 4	AY551905	-	-	-
SCUTICOCILIATIA	*Uronema marinum* pop. 5	DQ867072	-	-	-
SCUTICOCILIATIA	***Uronema marinum* pop. 6**	GQ259744	GQ259754	-	-
SCUTICOCILIATIA	***Uronema marinum* pop. 7**	GQ259749	GQ259755	-	-
SCUTICOCILIATIA	***Uronema marinum* pop. 8**	GQ465466	JN885102	JN885124	MH605541
SCUTICOCILIATIA	*Uronema marinum* pop. 9	MF418591	-	-	-
SCUTICOCILIATIA	***Uronema marinum* pop. 10**	MF992240	MF992244	MF992248	MG001900
SCUTICOCILIATIA	***Uronema marinum* pop. 11**	MF992241	MF992245	MF992249	MG001901
SCUTICOCILIATIA	***Uronema marinum* pop. 12**	MF992242	MF992246	MF992250	MG001902
SCUTICOCILIATIA	***Uronema marinum* pop. 13**	MF992243	MF992247	MF992251	MG001903
SCUTICOCILIATIA	*Uronema marinum* pop. 14	Z22881	-	-	-
SCUTICOCILIATIA	***Uronema nigricans***	MF072399	**PP852883**	**PP784543**	-
SCUTICOCILIATIA	***Uronema orientalis* pop. 1**	KF840517	-	-	MH605553
SCUTICOCILIATIA	*Uronema orientalis* pop. 2	MH574791	-	-	-
SCUTICOCILIATIA	***Uronema orientalis* pop. 3**	MT982806	**PP852880**	**PP784538**	**PQ256151**
SCUTICOCILIATIA	*Uronema* sp. 1	FJ936001	-	-	-
SCUTICOCILIATIA	***Uronema* sp. 2**	JN885088	JN885107	JN885125	MH605557
SCUTICOCILIATIA	*Uronema* sp. 3	KY569295	-	-	-
SCUTICOCILIATIA	*Uronema* sp. 4	MG452736	-	-	-
SCUTICOCILIATIA	*Uronemita filificum* pop. 1	EF486866	-	-	-
SCUTICOCILIATIA	***Uronemita filificum* pop. 2**	MH574793	**PP852882**	**PP784542**	MH605567
SCUTICOCILIATIA	*Uronemita filificum* pop. 3	-	-	-	MH605560
SCUTICOCILIATIA	***Uronemita parabinucleata***	KU199245	-	**PP784537**	MH605555
SCUTICOCILIATIA	***Uronemita parafilificum* pop. 1**	HM236337	JN885103	JN885127	MH605564
SCUTICOCILIATIA	*Uronemita parafilificum* pop. 2	MW082832	-	-	-
SCUTICOCILIATIA	***Uronemita sinensis***	JN885083	JN885108	JN885126	-
SCUTICOCILIATIA	*Uronemita* sp.	KT266872	-	-	-
PERITRICHIA	***Vorticella campanula***	DQ662849	KF524384	-	-
SCUTICOCILIATIA	*Wilbertia typica* pop. 1	FJ490551	-	-	-
SCUTICOCILIATIA	***Wilbertia typica* pop. 2**	JX310022	JX310031	JX310031	-
PERITRICHIA	***Zoothamnium hentscheli***	KM222118	KM222089	KM222178	-

Species/Populations names in bold indicate the corresponding sequences were used in the construction of the concatenated trees.
Accession numbers in bold are newly characterized gene sequences

Supplementary Table S3 Information of Scuticociliatia-related taxa and environmental or uncultured SSU rRNA gene sequences used in the present study

Subclass or Order	Family or environmental	Sequence name	GenBank accession no.	Short ID	Source	Location	GC content (%)
Loxocephalida	Cinetochilidae	KF301567_Platynematum_salinarum	KF301567	Platynematum salinarum	Salt water	Portugal	45.00
Loxocephalida	Cinetochilidae	JX310020_Pseudoplatynematum_denticulatum	JX310020	Pseudoplatynematum denticulatum	Marine	China	44.45
Loxocephalida	Conchophthiridae	JQ956543_Conchophthirus_cucumis	JQ956543	Conchophthirus cucumis	Parasitic (marine host)	China	42.63
Loxocephalida	Conchophthiridae	MN704274_Conchophthirus_curtus	MN704274	Conchophthirus curtus	Parasitic (freshwater host)	Canada	42.78
Loxocephalida	Conchophthiridae	JQ956542_Conchophthirus_lamellid	JQ956542	Conchophthirus lamellidens	Parasitic (freshwater host)	China	42.52
Loxocephalida	Conchophthiridae	MN704275_Conchophthirus_sp.	MN704275	Conchophthirus sp.	Parasitic (freshwater host)	Canada	42.89
Loxocephalida	Conchophthiridae	MN704276_Conchophthirus_sp.	MN704276	Conchophthirus sp.	Parasitic (freshwater host)	Canada	42.91
Loxocephalida	Conchophthiridae	MN704277_Conchophthirus_sp.	MN704277	Conchophthirus sp.	Parasitic (freshwater host)	Canada	42.80
Loxocephalida	Conchophthiridae	MN704278_Conchophthirus_sp.	MN704278	Conchophthirus sp.	Parasitic (freshwater host)	Canada	42.79
Loxocephalida	*Incertae sedis*	FJ868188_Sathrophilus_holtae	FJ868188	Sathrophilus holtae	Marine	China	44.26
Loxocephalida	*Incertae sedis*	FJ868186_Sathrophilus_planus	FJ868186	Sathrophilus planus	Marine	China	43.56
Loxocephalida	Loxocephalidae	AY881632_Cardiostomatella_vermiformis	AY881632	Cardiostomatella vermiformis	Marine	China	45.01
Loxocephalida	Loxocephalidae	KF878931_Dexiotricha_cf._granulosa	KF878931	Dexiotricha cf. granulosa	Freshwater	China	46.35
Loxocephalida	Loxocephalidae	MG819725_Dexiotricha_colpidiopsis	MG819725	Dexiotricha colpidiopsis	Freshwater	Iceland	43.24
Loxocephalida	Loxocephalidae	KF878932_Dexiotricha_elliptica	KF878932	Dexiotricha elliptica	Freshwater	Saudi Arabia	44.79
Loxocephalida	Loxocephalidae	JQ723963_Dexiotricha_sp.	JQ723963	Dexiotricha sp.	Freshwater	Jamaica	46.58
Loxocephalida	Loxocephalidae	MK354012_Dexiotricha_sp.	MK354012	Dexiotricha sp.	Freshwater	India	46.14
Loxocephalida	Loxocephalidae	MN704273_Dexiotricha_sp.	MN704273	Dexiotricha sp.	Freshwater	Italy	43.31
Loxocephalida	Loxocephalidae	AY212805_Dexiotrichides_pangi	AY212805	Dexiotrichides pangi	Marine	China	43.63
Loxocephalida	Loxocephalidae	FJ876969_Paratetrahymena_parawassi	FJ876969	Paratetrahymena parawassi	Marine	China	47.26
Loxocephalida	Loxocephalidae	EU744176_Paratetrahymena_sp.	EU744176	Paratetrahymena sp.	Marine	China	46.80
Loxocephalida	Loxocephalidae	GQ292767_Paratetrahymena_wassi	GQ292767	Paratetrahymena wassi	Marine	China	47.46
Loxocephalida	Loxocephalidae	JX310019_Paratetrahymena_wassi	JX310019	Paratetrahymena wassi	Marine	China	46.66
Philasterida	Citrithrixidae	MT982807_Citrithrix_smalli	MT982807	Citrithrix smalli	Marine	China	45.00
Philasterida	Cohnilembidae	HM236339_Cohnilembus_verminus	HM236339	Cohnilembus verminus	Marine	China	44.57
Philasterida	Cohnilembidae	Z22878_Cohnilembus_verminus	Z22878	Cohnilembus verminus	Marine	UK	44.63
Philasterida	Cryptochilidae	JQ956541_Biggaria_bermudensis	JQ956541	Biggaria bermudensis	Parasitic (freshwater host)	China	44.18
Philasterida	Entodiscidae	AY541687_Entodiscus_borealis	AY541687	Entodiscus borealis	Marine	Canada	42.13
Philasterida	Entorhipidiidae	JQ956545_Entorhipidium_enchi	JQ956545	Entorhipidium enchi	Marine	China	43.58
Philasterida	Entorhipidiidae	AY541689_Entorhipidium_pilatum	AY541689	Entorhipidium pilatum	Marine	Canada	45.34
Philasterida	Entorhipidiidae	AY541688_Entorhipidium_tenue	AY541688	Entorhipidium tenue	Marine	Canada	44.05
Philasterida	Entorhipidiidae	JQ956544_Entorhipidium_tenue	JQ956544	Entorhipidium tenue	Marine	China	43.93
Philasterida	Entorhipidiidae	AY541690_Entorhipidium_triangularis	AY541690	Entorhipidium triangularis	Marine	Canada	44.54
Philasterida	Homalogastridae	MG581969_Homalogastra_parasetosa	MG581969	Homalogastra parasetosa	Brackish water	China	43.89
Philasterida	Homalogastridae	EF158847_Homalogastra_setosa	EF158847	Homalogastra setosa	Soil	Germany	44.30
Philasterida	Homalogastridae	EF158848_Homalogastra_setosa	EF158848	Homalogastra setosa	Soil	Germany	44.53
Philasterida	Homalogastridae	GU590870_Homalogastra_setosa	GU590870	Homalogastra setosa	Soil	South Korea	44.46
Philasterida	Homalogastridae	MT982808_Homalogastra_binucleata	MT982808	Homalogastra similis	Freshwater	China	43.64
Philasterida	*Incertae sedis*	GQ214552_Glauconema_trihymene	GQ214552	Glauconema trihymene	Marine	China	43.76
Philasterida	*Incertae sedis*	JQ956550_Madsenia_indomita	JQ956550	Madsenia indomita	Marine	China	42.75
Philasterida	*Incertae sedis*	MT271859_Madsenia_sp.	MT271859	Madsenia sp.	Parasitic (marine host)	Saint Kitts and Nevis	41.60
Philasterida	*Incertae sedis*	AY550080_Miamiensis_avidus	AY550080	Miamiensis avidus	Parasitic (marine host)	South Korea	44.87
Philasterida	*Incertae sedis*	AY642280_Miamiensis_avidus	AY642280	Miamiensis avidus	Marine	South Korea	44.93
Philasterida	*Incertae sedis*	EU831192_Miamiensis_avidus	EU831192	Miamiensis avidus	Marine	South Korea	44.49
Philasterida	*Incertae sedis*	EU831193_Miamiensis_avidus	EU831193	Miamiensis avidus	Marine	South Korea	44.49
Philasterida	*Incertae sedis*	EU831194_Miamiensis_avidus	EU831194	Miamiensis avidus	Marine	South Korea	44.49
Philasterida	*Incertae sedis*	EU831195_Miamiensis_avidus	EU831195	Miamiensis avidus	Marine	South Korea	44.49
Philasterida	*Incertae sedis*	EU831196_Miamiensis_avidus	EU831196	Miamiensis avidus	Marine	South Korea	44.49
Philasterida	*Incertae sedis*	EU831198_Miamiensis_avidus	EU831198	Miamiensis avidus	Marine	South Korea	44.49
Philasterida	*Incertae sedis*	JN689229_Miamiensis_avidus	JN689229	Miamiensis avidus	Marine	South Korea	44.48
Philasterida	*Incertae sedis*	JN689230_Miamiensis_avidus	JN689230	Miamiensis avidus	Marine	South Korea	44.87
Philasterida	*Incertae sedis*	JN885091_Miamiensis_avidus	JN885091	Miamiensis avidus	Marine	China	44.14
Philasterida	*Incertae sedis*	KU720304_Miamiensis_avidus	KU720304	Miamiensis avidus	Marine	China	44.46
Philasterida	*Incertae sedis*	KU992658_Miamiensis_avidus	KU992658	Miamiensis avidus	Marine	China	44.87
Philasterida	*Incertae sedis*	KX259260_Miamiensis_avidus	KX259260	Miamiensis avidus	Marine	Peru	44.85
Philasterida	*Incertae sedis*	KX357144_Miamiensis_avidus	KX357144	Miamiensis avidus	Marine	Spain	43.85
Philasterida	*Incertae sedis*	KY082893_Miamiensis_avidus	KY082893	Miamiensis avidus	Marine	China	44.45
Philasterida	*Incertae sedis*	MN611447_Miamiensis_avidus	MN611447	Miamiensis avidus	Marine	China	44.85
Philasterida	*Incertae sedis*	MN611448_Miamiensis_avidus	MN611448	Miamiensis avidus	Marine	China	44.85
Philasterida	*Incertae sedis*	FJ936000_Miamiensis_sp.	FJ936000	Miamiensis sp.	Marine	New Zealand	46.51
Philasterida	*Incertae sedis*	GU572375_Philasterides_dicentrarchi	GU572375	Philasterides dicentrarchi	Marine	South Korea	44.54
Philasterida	*Incertae sedis*	JX914665_Philasterides_dicentrarchi	JX914665	Philasterides dicentrarchi	Marine	Spain	44.79
Philasterida	*Incertae sedis*	MK002746_Philasterides_dicentrarchi	MK002746	Philasterides dicentrarchi	Parasitic (marine host)	Spain	44.43
Philasterida	Orchitophryidae	U51554_Anophyroides_haemophila	U51554	Anophyroides haemophila	Parasitic (marine host)	Canada	43.68
Philasterida	Orchitophryidae	AY103189_Mesanophrys_carcini	AY103189	Mesanophrys carcini	Marine	China	42.94
Philasterida	Orchitophryidae	JN885085_Mesanophrys_carcini	JN885085	Mesanophrys carcini	Marine	China	42.67
Philasterida	Orchitophryidae	JN885086_Mesanophrys_carcini	JN885086	Mesanophrys carcini	Marine	China	43.10
Philasterida	Orchitophryidae	FJ936002_Mesanophrys_sp.	FJ936002	Mesanophrys sp.	Marine	New Zealand	44.08
Philasterida	Orchitophryidae	MN260367_Mesanophrys_sp.	MN260367	Mesanophrys sp.	Parasitic (marine host)	China	44.01
Philasterida	Orchitophryidae	JN885084_Metanophrys_orientalis	JN885084	Metanophrys orientalis	Marine	China	44.71
Philasterida	Orchitophryidae	AY314803_Metanophrys_similis	AY314803	Metanophrys similis	Marine	China	43.31
Philasterida	Orchitophryidae	HM236336_Metanophrys_sinensis	HM236336	Metanophrys sinensis	Marine	China	43.94
Philasterida	Orchitophryidae	MH574792_Paramesanophrys_typica	MH574792	Paramesanophrys typica	Marine	China	44.03
Philasterida	Orchitophryidae	AY103191_Paranophrys_magna	AY103191	Paranophrys magna	Marine	China	43.70
Philasterida	Orchitophryidae	JN885089_Paranophrys_magna	JN885089	Paranophrys magna	Marine	China	43.72
Philasterida	Orchitophryidae	JQ956548_Paranophrys_magna	JQ956548	Paranophrys magna	Marine	Germany	43.66
Philasterida	Orchitophryidae	FJ858379_Paranophrys_marina	FJ858379	Paranophrys marina	Marine	South Korea	43.67
Philasterida	Paralembidae	JQ956549_Paralembus_digitiformis	JQ956549	Paralembus digitiformis	Marine	China	44.39
Philasterida	Philasteridae	FJ648350_Philaster_apodigitiform	FJ648350	Philaster apodigitiformis	Marine	China	44.28
Philasterida	Philasteridae	KJ815049_Philaster_sinensis	KJ815049	Philaster sinensis	Marine	China	43.88
Philasterida	Philasteridae	FJ848877_Philasterides_armatalis	FJ848877	Philasterides armatalis	Marine	China	44.02
Philasterida	Philasteridae	HM236335_Porpostoma_notata	HM236335	Porpostoma notata	Marine	China	44.09
Philasterida	Pseudocohnilembidae	AY212806_Pseudocohnilembus_hargisi	AY212806	Pseudocohnilembus hargisi	Marine	China	44.61
Philasterida	Pseudocohnilembidae	AY833087_Pseudocohnilembus_hargisi	AY833087	Pseudocohnilembus hargisi	Marine	South Korea	44.56
Philasterida	Pseudocohnilembidae	JN885090_Pseudocohnilembus_hargisi	JN885090	Pseudocohnilembus hargisi	Marine	China	44.78
Philasterida	Pseudocohnilembidae	FJ899594_Pseudocohnilembus_longisetus	FJ899594	Pseudocohnilembus longisetus	Marine	South Korea	44.44
Philasterida	Pseudocohnilembidae	Z22880_Pseudocohnilembus_marinus	Z22880	Pseudocohnilembus marinus	Marine	UK	44.41
Philasterida	Pseudocohnilembidae	AY551906_Pseudocohnilembus_persalinus	AY551906	Pseudocohnilembus persalinus	Marine	South Korea	45.06
Philasterida	Pseudocohnilembidae	AY835669_Pseudocohnilembus_persalinus	AY835669	Pseudocohnilembus persalinus	Marine	South Korea	45.03
Philasterida	Pseudocohnilembidae	GQ265955_Pseudocohnilembus_persalinus	GQ265955	Pseudocohnilembus persalinus	Marine	China	44.97
Philasterida	Pseudocohnilembidae	GU584096_Pseudocohnilembus_persalinus	GU584096	Pseudocohnilembus persalinus	Freshwater	Canada	45.12
Philasterida	Pseudocohnilembidae	JQ956554_Pseudocohnilembus_persalinus	JQ956554	Pseudocohnilembus persalinus	Marine	China	44.91
Philasterida	Pseudocohnilembidae	MG452732_Pseudocohnilembus_persalinus	MG452732	Pseudocohnilembus persalinus	Marine	Germany	45.22
Philasterida	Pseudocohnilembidae	MG452733_Pseudocohnilembus_persalinus	MG452733	Pseudocohnilembus persalinus	Marine	Germany	45.17
Philasterida	Pseudocohnilembidae	MG452734_Pseudocohnilembus_persalinus	MG452734	Pseudocohnilembus persalinus	Marine	Germany	45.18
Philasterida	Pseudocohnilembidae	MG452735_Pseudocohnilembus_persalinus	MG452735	Pseudocohnilembus persalinus	Marine	Germany	45.13
Philasterida	Pseudocohnilembidae	MT081565_Pseudocohnilembus_persalinus	MT081565	Pseudocohnilembus persalinus	Marine	Caribbean Sea	45.33
Philasterida	Schizocaryidae	AF527756_Schizocaryum_dogieli	AF527756	Schizocaryum dogieli	Marine	Canada	44.29
Philasterida	Schizocaryidae	EU597807_Schizocaryum_sp.	EU597807	Schizocaryum sp.	Marine	Ireland	47.17
Philasterida	Thyrophylacidae	AY541685_Plagiopyliella_pacifica	AY541685	Plagiopyliella pacifica	Marine	Canada	44.05
Philasterida	Thyrophylacidae	AY541686_Thyrophylax_vorax	AY541686	Thyrophylax vorax	Marine	Canada	43.47
Philasterida	Uronematidae	MN524103_Apouronema_harbinensis	MN524103	Apouronema harbinensis	Freshwater	China	43.45
Philasterida	Uronematidae	FJ595488_Parauronema_cf._virginianum	FJ595488	Parauronema cf. virginianum	Marine	China	42.15
Philasterida	Uronematidae	JN885082_Parauronema_cf._virginianum	JN885082	Parauronema cf. virginianum	Marine	China	42.10
Philasterida	Uronematidae	AY212807_Parauronema_longum	AY212807	Parauronema longum	Marine	China	44.96
Philasterida	Uronematidae	HM236338_Parauronema_longum	HM236338	Parauronema longum	Marine	China	44.63
Philasterida	Uronematidae	AY392128_Parauronema_virginianum	AY392128	Parauronema virginianum	Marine	China	42.15
Philasterida	Uronematidae	JN885087_Parauronema_virginianum	JN885087	Parauronema virginianum	Marine	China	42.21
Philasterida	Uronematidae	MG581965_Uronema_apomarinum	MG581965	Uronema apomarinum	Brackish water	China	43.28
Philasterida	Uronematidae	AY103190_Uronema_elegans	AY103190	Uronema elegans	Marine	China	43.11
Philasterida	Uronematidae	FJ870100_Uronema_heteromarinum	FJ870100	Uronema heteromarinum	Marine	China	43.29
Philasterida	Uronematidae	AY551905_Uronema_marinum	AY551905	Uronema marinum	Marine	South Korea	42.23
Philasterida	Uronematidae	DQ867072_Uronema_marinum	DQ867072	Uronema marinum	Marine	South Korea	42.21
Philasterida	Uronematidae	GQ259744_Uronema_marinum	GQ259744	Uronema marinum	Marine	Spain	42.29
Philasterida	Uronematidae	GQ259749_Uronema_marinum	GQ259749	Uronema marinum	Marine	Tasman Sea	42.09
Philasterida	Uronematidae	GQ465466_Uronema_marinum	GQ465466	Uronema marinum	Marine	China	42.21
Philasterida	Uronematidae	MF418591_Uronema_marinum	MF418591	Uronema marinum	Marine	China	42.26
Philasterida	Uronematidae	MF992240_Uronema_marinum	MF992240	Uronema marinum	Marine	China	42.28
Philasterida	Uronematidae	MF992241_Uronema_marinum	MF992241	Uronema marinum	Marine	China	42.32
Philasterida	Uronematidae	MF992242_Uronema_marinum	MF992242	Uronema marinum	Marine	China	42.31
Philasterida	Uronematidae	MF992243_Uronema_marinum	MF992243	Uronema marinum	Marine	China	42.43
Philasterida	Uronematidae	Z22881_Uronema_marinum	Z22881	Uronema marinum	Marine	UK	42.43
Philasterida	Uronematidae	MF072399_Uronema_nigricans	MF072399	Uronema nigricans	Freshwater	China	43.13
Philasterida	Uronematidae	KF840517_Uronema_orientalis	KF840517	Uronema orientalis	Marine	China	42.38
Philasterida	Uronematidae	MH574791_Uronema_orientalis	MH574791	Uronema orientalis	Marine	China	42.32
Philasterida	Uronematidae	MT982806_Uronema_orientalis	MT982806	Uronema orientalis	Marine	China	42.38
Philasterida	Uronematidae	FJ936001_Uronema_sp.	FJ936001	Uronema sp.	Marine	New Zealand	43.80
Philasterida	Uronematidae	JN885088_Uronema_sp.	JN885088	Uronema sp.	Marine	China	42.32

Philasterida	Uronematidae	KY569295_Uronema_sp.	KY569295	Uronema sp.	Marine	France	42.03
Philasterida	Uronematidae	MG452736_Uronema_sp.	MG452736	Uronema sp.	Marine	Germany	42.91
Philasterida	Uronematidae	EF486866_Uronemella_filificum	EF486866	Uronemella filificum	Marine	China	43.15
Philasterida	Uronematidae	MH574793_Uronemita_filificum	MH574793	Uronemita filificum	Brackish water	China	43.08
Philasterida	Uronematidae	KU199245_Uronemita_parabinucleata	KU199245	Uronemita parabinucleata	Marine	China	42.78
Philasterida	Uronematidae	HM236337_Uronemita_parafilificum	HM236337	Uronemita parafilificum	Marine	China	42.43
Philasterida	Uronematidae	MW082832_Uronemella_parafilificum	MW082832	Uronemita parafilificum	Marine	China	43.08
Philasterida	Uronematidae	JN885083_Uronemita_sinensis	JN885083	Uronemita sinensis	Marine	China	42.97
Philasterida	Uronematidae	KT266872_Uronemita_sp.	KT266872	Uronemita sp.	Salt water	USA	42.91
Pleuronematida	Ancistridae	HM236340_Ancistrum_crassum	HM236340	Ancistrum crassum	Parasitic (marine host)	China	43.91
Pleuronematida	Ancistridae	HQ445964_Ancistrum_crassum	HQ445964	Ancistrum crassum	Parasitic (marine host)	China	44.07
Pleuronematida	Ancistridae	JQ956537_Ancistrum_crassum	JQ956537	Ancistrum crassum	Parasitic (marine host)	China	43.82
Pleuronematida	Ancistridae	JQ956538_Ancistrum_crassum	JQ956538	Ancistrum crassum	Parasitic (marine host)	China	43.88
Pleuronematida	Ancistridae	JQ956539_Ancistrum_crassum	JQ956539	Ancistrum crassum	Parasitic (marine host)	China	44.06
Pleuronematida	Ancistridae	MF407351_Ancistrum_crassum	MF407351	Ancistrum crassum	Parasitic (marine host)	China	44.05
Pleuronematida	Ancistridae	JQ956536_Ancistrum_japonicum	JQ956536	Ancistrum japonicum	Parasitic (marine host)	China	43.88
Pleuronematida	Ancistridae	JQ956535_Ancistrum_mytili	JQ956535	Ancistrum mytili	Parasitic (marine host)	China	43.96
Pleuronematida	Ancistridae	HQ445963_Ancistrum_sp.	HQ445963	Ancistrum sp.	Parasitic (marine host)	China	43.98
Pleuronematida	Ancistridae	JQ956552_Protophyra_ovicola	JQ956552	Protophyra ovicola	Marine	China	44.71
Pleuronematida	Ctedoctematidae	FJ868182_Falcicyclidium_atractodes	FJ868182	Falcicyclidium atractodes	Marine	China	43.59
Pleuronematida	Ctedoctematidae	FJ868183_Falcicyclidium_fangi	FJ868183	Falcicyclidium fangi	Marine	China	42.83
Pleuronematida	Ctedoctematidae	FJ868184_Falcicyclidium_fangi	FJ868184	Falcicyclidium fangi	Marine	China	42.91
Pleuronematida	Ctedoctematidae	FJ868185_Falcicyclidium_fangi	FJ868185	Falcicyclidium fangi	Marine	China	42.18
Pleuronematida	Ctedoctematidae	FJ868181_Falcicyclidium_plouneouri	FJ868181	Falcicyclidium plouneouri	Marine	China	42.04
Pleuronematida	Ctedoctematidae	KF256818_Cyclidium_plouneouri	KF256818	Falcicyclidium plouneouri	Marine	China	42.04
Pleuronematida	Ctedoctematidae	KF256819_Cyclidium_plouneouri	KF256819	Falcicyclidium plouneouri	Marine	China	42.16
Pleuronematida	Ctedoctematidae	U27816_Falcicyclidium_plouneouri	U27816	Falcicyclidium plouneouri	Marine	UK	41.33
Pleuronematida	Ctedoctematidae	JX310012_Hippocomos_salinus	JX310012	Hippocomos salinus	Marine	China	42.75
Pleuronematida	Cyclidiidae	FJ868180_Cristigera_media	FJ868180	Cristigera media	Marine	China	42.75
Pleuronematida	Cyclidiidae	KF256816_Cristigera_pleuronemoides	KF256816	Cristigera pleuronemoides	Marine	China	42.95
Pleuronematida	Cyclidiidae	EU032356_Cyclidium_glaucoma	EU032356	Cyclidium glaucoma	Freshwater	Switzerland	45.30
Pleuronematida	Cyclidiidae	KY476313_Cyclidium_glaucoma	KY476313	Cyclidium glaucoma	Marine	China	45.18
Pleuronematida	Cyclidiidae	KY886366_Cyclidium_glaucoma	KY886366	Cyclidium glaucoma	Marine	China	45.56
Pleuronematida	Cyclidiidae	Z22879_Cyclidium_glaucoma	Z22879	Cyclidium glaucoma	Marine	UK	44.96
Pleuronematida	Cyclidiidae	JQ956553_Cyclidium_marinum	JQ956553	Cyclidium marinum	Marine	Germany	44.20
Pleuronematida	Cyclidiidae	KY886367_Cyclidium_marinum	KY886367	Cyclidium marinum	Marine	China	44.03
Pleuronematida	Cyclidiidae	KX853100_Cyclidium_sinicum	KX853100	Cyclidium sinicum	Freshwater	China	45.44
Pleuronematida	Cyclidiidae	KF256817_Cyclidium_varibonneti	KF256817	Cyclidium varibonneti	Marine	China	44.17
Pleuronematida	Cyclidiidae	MN524102_Cyclidium_vorax	MN524102	Cyclidium vorax	Freshwater	China	45.75
Pleuronematida	Cyclidiidae	KF256820_Protocyclidium_citrullus	KF256820	Protocyclidium citrullus	Marine	China	42.90
Pleuronematida	Cyclidiidae	KF256821_Protocyclidium_citrullus	KF256821	Protocyclidium citrullus	Marine	China	43.16
Pleuronematida	Cyclidiidae	KF256822_Protocyclidium_sinica	KF256822	Protocyclidium sinica	Marine	China	47.21
Pleuronematida	Cyclidiidae	JQ956551_Pseudocyclidium_longum	JQ956551	Pseudocyclidium longum	Marine	China	44.41
Pleuronematida	Eurystomatellidae	FJ012143_Eurystomatella_sinica	FJ012143	Eurystomatella sinica	Marine	China	42.58
Pleuronematida	Eurystomatellidae	JX310021_Eurystomatella_sinica	JX310021	Eurystomatella sinica	Marine	China	42.64
Pleuronematida	Eurystomatellidae	FJ490551_Wilbertia_typica	FJ490551	Wilbertia typica	Marine	China	42.70
Pleuronematida	Eurystomatellidae	JX310022_Wilbertia_typica	JX310022	Wilbertia typica	Marine	China	42.82
Pleuronematida	Hemispeiridae	MF407350_Boveria_labialis	MF407350	Boveria labialis	Parasitic (marine host)	China	42.11
Pleuronematida	Hemispeiridae	FJ848878_Boveria_subcylindrica	FJ848878	Boveria subcylindrica	Parasitic (marine host)	China	42.43
Pleuronematida	Hemispeiridae	HQ445965_Boveria_subcylindrica	HQ445965	Boveria subcylindrica	Parasitic (marine host)	China	42.53
Pleuronematida	Histiobalantiidae	MT886454_Histiobalantium_bodamicum	MT886454	Histiobalantium bodamicum	Freshwater	Austria	42.16
Pleuronematida	Histiobalantiidae	KU665372_Histiobalantium_comosa	KU665372	Histiobalantium comosa	Freshwater	Canada	41.89
Pleuronematida	Histiobalantiidae	JX310013_Histiobalantium_minor	JX310013	Histiobalantium minor	Marine	China	41.84
Pleuronematida	Histiobalantiidae	AB450957_Histiobalantium_natans	AB450957	Histiobalantium natans	Freshwater	Japan	42.04
Pleuronematida	Peniculistomatidae	JQ956546_Mytilophilus_pacificae	JQ956546	Mytilophilus pacificae	Marine	China	42.88
Pleuronematida	Peniculistomatidae	KU665346_Mytilophilus_pacificae	KU665346	Mytilophilus pacificae	Parasitic (marine host)	Canada	43.05
Pleuronematida	Peniculistomatidae	KU665347_Mytilophilus_pacificae	KU665347	Mytilophilus pacificae	Parasitic (marine host)	Canada	43.05
Pleuronematida	Peniculistomatidae	KU665348_Mytilophilus_pacificae	KU665348	Mytilophilus pacificae	Parasitic (marine host)	Canada	43.10
Pleuronematida	Peniculistomatidae	KU665349_Mytilophilus_pacificae	KU665349	Mytilophilus pacificae	Parasitic (marine host)	Canada	43.05
Pleuronematida	Peniculistomatidae	KU665350_Mytilophilus_pacificae	KU665350	Mytilophilus pacificae	Parasitic (marine host)	Canada	43.05
Pleuronematida	Peniculistomatidae	KU665351_Mytilophilus_pacificae	KU665351	Mytilophilus pacificae	Parasitic (marine host)	Canada	43.08
Pleuronematida	Peniculistomatidae	KU665353_Mytilophilus_pacificae	KU665353	Mytilophilus pacificae	Parasitic (marine host)	Canada	42.77
Pleuronematida	Peniculistomatidae	KU665354_Mytilophilus_pacificae	KU665354	Mytilophilus pacificae	Parasitic (marine host)	Canada	42.77
Pleuronematida	Peniculistomatidae	KU665355_Peniculistoma_mytili	KU665355	Peniculistoma mytili	Parasitic (marine host)	Canada	42.77
Pleuronematida	Peniculistomatidae	KU665356_Peniculistoma_mytili	KU665356	Peniculistoma mytili	Parasitic (marine host)	Canada	42.77
Pleuronematida	Peniculistomatidae	KU665357_Peniculistoma_mytili	KU665357	Peniculistoma mytili	Parasitic (marine host)	Canada	42.82
Pleuronematida	Peniculistomatidae	KU665358_Peniculistoma_mytili	KU665358	Peniculistoma mytili	Parasitic (marine host)	Canada	42.82
Pleuronematida	Peniculistomatidae	KU665359_Peniculistoma_mytili	KU665359	Peniculistoma mytili	Parasitic (marine host)	Canada	42.82
Pleuronematida	Peniculistomatidae	KU665360_Peniculistoma_mytili	KU665360	Peniculistoma mytili	Parasitic (marine host)	Canada	42.87
Pleuronematida	Peniculistomatidae	KU665361_Peniculistoma_mytili	KU665361	Peniculistoma mytili	Parasitic (marine host)	Canada	42.89
Pleuronematida	Peniculistomatidae	KU665362_Peniculistoma_mytili	KU665362	Peniculistoma mytili	Parasitic (marine host)	Canada	42.92
Pleuronematida	Peniculistomatidae	KU665363_Peniculistoma_mytili	KU665363	Peniculistoma mytili	Parasitic (marine host)	Canada	42.89
Pleuronematida	Peniculistomatidae	KU665364_Peniculistoma_mytili	KU665364	Peniculistoma mytili	Parasitic (marine host)	Canada	42.87
Pleuronematida	Peniculistomatidae	KU665365_Peniculistoma_mytili	KU665365	Peniculistoma mytili	Parasitic (marine host)	Canada	42.82
Pleuronematida	Peniculistomatidae	KU665366_Peniculistoma_mytili	KU665366	Peniculistoma mytili	Parasitic (marine host)	Canada	42.82
Pleuronematida	Peniculistomatidae	KU665367_Peniculistoma_mytili	KU665367	Peniculistoma mytili	Parasitic (marine host)	Canada	42.82
Pleuronematida	Peniculistomatidae	KU665368_Peniculistoma_mytili	KU665368	Peniculistoma mytili	Parasitic (marine host)	Canada	42.82
Pleuronematida	Peniculistomatidae	KU665369_Peniculistoma_mytili	KU665369	Peniculistoma mytili	Parasitic (marine host)	Canada	42.92
Pleuronematida	Peniculistomatidae	KU665370_Peniculistoma_mytili	KU665370	Peniculistoma mytili	Parasitic (marine host)	Canada	42.92
Pleuronematida	Peniculistomatidae	KU665371_Peniculistoma_mytili	KU665371	Peniculistoma mytili	Parasitic (marine host)	Canada	42.92
Pleuronematida	Pleuronematidae	KT033424_Pleuronema_binucleatum	KT033424	Pleuronema binucleatum	Brackish water	China	42.75
Pleuronematida	Pleuronematidae	FJ848875_Pleuronema_cf._setigerum	FJ848875	Pleuronema cf. setigerum	Marine	China	43.01
Pleuronematida	Pleuronematidae	AY103188_Pleuronema_coronatum	AY103188	Pleuronema coronatum	Marine	China	42.77
Pleuronematida	Pleuronematidae	HM140396_Pleuronema_coronatum	HM140396	Pleuronema coronatum	Unknown	South Korea	42.77
Pleuronematida	Pleuronematidae	JX310014_Pleuronema_coronatum	JX310014	Pleuronema coronatum	Marine	China	42.51
Pleuronematida	Pleuronematidae	JX310018_Pleuronema_coronatum	JX310018	Pleuronema coronatum	Marine	China	42.82
Pleuronematida	Pleuronematidae	EF486863_Pleuronema_czapikae	EF486863	Pleuronema czapikae	Marine	China	42.00
Pleuronematida	Pleuronematidae	KF840518_Pleuronema_elegans	KF840518	Pleuronema elegans	Marine	China	42.73
Pleuronematida	Pleuronematidae	OL654416_Pleuronema_foissneri	OL654416	Pleuronema foissneri	Brackish water	China	43.01
Pleuronematida	Pleuronematidae	KF840519_Pleuronema_grolierei	KF840519	Pleuronema grolierei	Marine	China	42.98
Pleuronematida	Pleuronematidae	KF206428_Pleuronema_marinum	KF206428	Pleuronema marinum	Brackish water	China	42.50
Pleuronematida	Pleuronematidae	KF206429_Pleuronema_orientale	KF206429	Pleuronema orientale	Brackish water	China	43.87
Pleuronematida	Pleuronematidae	OL654419_Pleuronema_paraorientale	OL654419	Pleuronema paraorientale	Brackish water	China	43.50
Pleuronematida	Pleuronematidae	OL654418_Pleuronema_parasalmastra	OL654418	Pleuronema parasalmastra	Brackish water	China	44.36
Pleuronematida	Pleuronematidae	OL654417_Pleuronema_parasmalli	OL654417	Pleuronema parasmalli	Freshwater	China	43.26
Pleuronematida	Pleuronematidae	KT033423_Pleuronema_parawiackowskii	KT033423	Pleuronema parawiackowskii	Brackish water	China	41.99
Pleuronematida	Pleuronematidae	KF206430_Pleuronema_paucisaetosum	KF206430	Pleuronema paucisaetosum	Brackish water	China	43.27
Pleuronematida	Pleuronematidae	KF840520_Pleuronema_puytoraci	KF840520	Pleuronema puytoraci	Marine	China	43.35
Pleuronematida	Pleuronematidae	FJ848874_Pleuronema_setigerum	FJ848874	Pleuronema setigerum	Marine	China	40.84
Pleuronematida	Pleuronematidae	JX310015_Pleuronema_setigerum	JX310015	Pleuronema setigerum	Marine	China	42.81
Pleuronematida	Pleuronematidae	EF486864_Pleuronema_sinica	EF486864	Pleuronema sinica	Marine	China	42.19
Pleuronematida	Pleuronematidae	FJ848876_Pleuronema_sp.	FJ848876	Pleuronema sp.	Marine	China	42.21
Pleuronematida	Pleuronematidae	JX310017_Pleuronema_sp.	JX310017	Pleuronema sp.	Marine	China	41.96
Pleuronematida	Pleuronematidae	JX310016_Pleuronema_wiackowskii	JX310016	Pleuronema wiackowskii	Marine	China	41.95
Pleuronematida	Pleuronematidae	DQ777744_Schizocalyptra_aeschtae	DQ777744	Schizocalyptra aeschtae	Marine	China	40.57
Pleuronematida	Pleuronematidae	EU744177_Schizocalyptra_similis	EU744177	Schizocalyptra similis	Marine	China	42.25
Pleuronematida	Pleuronematidae	FJ156106_Schizocalyptra_sinica	FJ156106	Schizocalyptra sinica	Marine	China	41.05
Pleuronematida	Pleuronematidae	FJ848873_Schizocalyptra_sp.	FJ848873	Schizocalyptra sp.	Marine	China	42.19
Pleuronematida	Thigmophryidae	MT649635_Myxophyllum_steenstrupi	MT649635	Myxophyllum steenstrupi	Parasitic (terrestrial host)	Slovakia	44.83
Pleuronematida	Thigmophryidae	MT649636_Myxophyllum_steenstrupi	MT649636	Myxophyllum steenstrupi	Parasitic (terrestrial host)	Slovakia	44.83
Pleuronematida	Thigmophryidae	MT649637_Myxophyllum_steenstrupi	MT649637	Myxophyllum steenstrupi	Parasitic (terrestrial host)	Slovakia	44.83
Pleuronematida	Thigmophryidae	MT649638_Myxophyllum_steenstrupi	MT649638	Myxophyllum steenstrupi	Parasitic (terrestrial host)	Slovakia	44.83
Pleuronematida	Thigmophryidae	MT649639_Myxophyllum_steenstrupi	MT649639	Myxophyllum steenstrupi	Parasitic (terrestrial host)	Slovakia	44.83
Pleuronematida	Thigmophryidae	MT649640_Myxophyllum_steenstrupi	MT649640	Myxophyllum steenstrupi	Parasitic (terrestrial host)	Slovakia	44.83
	Environmental	AB330052_Uncultured_eukaryote	AB330052	Uncultured	Marine	Japan	45.57
	Environmental	AB505462_Uncultured_eukaryote	AB505462	Uncultured	Marine	Japan	44.87
	Environmental	AB505463_Uncultured_eukaryote	AB505463	Uncultured	Marine	Japan	42.92
	Environmental	AB505464_Uncultured_eukaryote	AB505464	Uncultured	Marine	Japan	44.10
	Environmental	AB505509_Uncultured_eukaryote	AB505509	Uncultured	Marine	Japan	43.58
	Environmental	AB505510_Uncultured_eukaryote	AB505510	Uncultured	Marine	Japan	44.94
	Environmental	AB505511_Uncultured_eukaryote	AB505511	Uncultured	Marine	Japan	43.54
	Environmental	AB695448_Uncultured_eukaryote	AB695448	Uncultured	Freshwater	Antarctica	44.80
	Environmental	AB725343_Uncultured_ciliate	AB725343	Uncultured	Marine	Denmark	45.87
	Environmental	AB749127_Uncultured_ciliate	AB749127	Uncultured	Freshwater	Japan	43.34
	Environmental	AY180044_Uncultured_ciliate	AY180044	Uncultured	Marine	USA	43.93
	Environmental	AY821919_Uncultured_pleuronemati	AY821919	Uncultured	Freshwater	France	43.29
	Environmental	AY821939_Uncultured_oligohymenop	AY821939	Uncultured	Freshwater	France	43.91
	Environmental	AY821941_Uncultured_oligohymenop	AY821941	Uncultured	Freshwater	France	46.11
	Environmental	AY876050_Uncultured_ciliate	AY876050	Uncultured	Marine	Australia	43.62
	Environmental	DQ103796_Uncultured_marine_eukaryote	DQ103796	Uncultured	Marine	Denmark	43.43

Environmental	DQ103831_Uncultured_marine_eukaryote	DQ103831	Uncultured	Marine	Denmark	44.99
Environmental	DQ103844_Uncultured_marine_eukaryote	DQ103844	Uncultured	Marine	Denmark	45.04
Environmental	DQ103857_Uncultured_marine_eukaryote	DQ103857	Uncultured	Marine	Denmark	44.41
Environmental	DQ103858_Uncultured_marine_eukaryote	DQ103858	Uncultured	Marine	Denmark	45.68
Environmental	DQ103868_Uncultured_marine_eukaryote	DQ103868	Uncultured	Marine	Denmark	45.38
Environmental	DQ103872_Uncultured_marine_eukaryote	DQ103872	Uncultured	Marine	Denmark	45.53
Environmental	DQ103877_Uncultured_marine_eukaryote	DQ103877	Uncultured	Marine	Denmark	46.70
Environmental	DQ244025_Uncultured_alveolate	DQ244025	Uncultured	Freshwater	France	41.82
Environmental	DQ310188_Uncultured_marine_eukaryote	DQ310188	Uncultured	Marine	Norway	42.94
Environmental	DQ310189_Uncultured_marine_eukaryote	DQ310189	Uncultured	Marine	Norway	43.00
Environmental	DQ310260_Uncultured_marine_eukaryote	DQ310260	Uncultured	Marine	Norway	46.02
Environmental	DQ310280_Uncultured_marine_eukaryote	DQ310280	Uncultured	Marine	Norway	43.80
Environmental	DQ310284_Uncultured_marine_eukaryote	DQ310284	Uncultured	Marine	Norway	42.45
Environmental	DQ310294_Uncultured_marine_eukaryote	DQ310294	Uncultured	Marine	Norway	42.79
Environmental	DQ310328_Uncultured_marine_eukaryote	DQ310328	Uncultured	Marine	Norway	42.83
Environmental	DQ310329_Uncultured_marine_eukaryote	DQ310329	Uncultured	Marine	Norway	43.04
Environmental	DQ310334_Uncultured_marine_eukaryote	DQ310334	Uncultured	Marine	Norway	42.98
Environmental	DQ504341_Uncultured_ciliate	DQ504341	Uncultured	Marine	Atlantic Ocean	45.16
Environmental	EF024585_Orchitophryidae	EF024585	Uncultured	Soil	USA	44.87
Environmental	EF526717_Uncultured_marine_eukaryote	EF526717	Uncultured	Marine	Norway	44.71
Environmental	EF526727_Uncultured_marine_eukaryote	EF526727	Uncultured	Marine	Norway	43.18
Environmental	EF526747_Uncultured_marine_eukaryote	EF526747	Uncultured	Marine	Norway	42.36
Environmental	EF526754_Uncultured_marine_eukaryote	EF526754	Uncultured	Marine	Norway	44.67
Environmental	EF526761_Uncultured_marine_eukaryote	EF526761	Uncultured	Marine	Norway	44.55
Environmental	EF526794_Uncultured_marine_eukaryote	EF526794	Uncultured	Marine	Norway	43.54
Environmental	EF526811_Uncultured_marine_eukaryote	EF526811	Uncultured	Marine	Norway	46.09
Environmental	EF526828_Uncultured_marine_eukaryote	EF526828	Uncultured	Marine	Norway	44.09
Environmental	EF526835_Uncultured_marine_eukaryote	EF526835	Uncultured	Marine	Norway	44.66
Environmental	EF526877_Uncultured_marine_eukaryote	EF526877	Uncultured	Marine	Norway	42.44
Environmental	EF526917_Uncultured_marine_eukaryote	EF526917	Uncultured	Marine	Norway	44.02
Environmental	EF526927_Uncultured_marine_eukaryote	EF526927	Uncultured	Marine	Norway	43.22
Environmental	EF526934_Uncultured_marine_eukaryote	EF526934	Uncultured	Marine	Norway	46.41
Environmental	EF526935_Uncultured_marine_eukaryote	EF526935	Uncultured	Marine	Norway	43.21
Environmental	EF526949_Uncultured_marine_eukaryote	EF526949	Uncultured	Marine	Norway	45.14
Environmental	EF526955_Uncultured_marine_eukaryote	EF526955	Uncultured	Marine	Norway	46.18
Environmental	EF527007_Uncultured_marine_eukaryote	EF527007	Uncultured	Marine	Norway	44.52
Environmental	EF527113_Uncultured_marine_eukaryote	EF527113	Uncultured	Marine	Norway	42.94
Environmental	EF527121_Uncultured_marine_eukaryote	EF527121	Uncultured	Marine	Norway	44.90
Environmental	EF527123_Uncultured_marine_eukaryote	EF527123	Uncultured	Marine	Norway	42.72
Environmental	EF527129_Uncultured_marine_eukaryote	EF527129	Uncultured	Marine	Norway	44.37
Environmental	EF527130_Uncultured_marine_eukaryote	EF527130	Uncultured	Marine	Norway	43.38
Environmental	EF527169_Uncultured_marine_eukaryote	EF527169	Uncultured	Marine	Norway	44.55
Environmental	EF527178_Uncultured_marine_eukaryote	EF527178	Uncultured	Marine	Norway	44.54
Environmental	EF527202_Uncultured_marine_eukaryote	EF527202	Uncultured	Marine	Norway	44.86
Environmental	EU446300_Uncultured_marine_eukaryote	EU446300	Uncultured	Marine	Mediterranean Sea	42.06
Environmental	EU446324_Uncultured_marine_eukaryote	EU446324	Uncultured	Marine	Mediterranean Sea	42.08
Environmental	EU446335_Uncultured_marine_eukaryote	EU446335	Uncultured	Marine	Mediterranean Sea	42.52
Environmental	EU446345_Uncultured_marine_eukaryote	EU446345	Uncultured	Marine	Mediterranean Sea	42.27
Environmental	EU446371_Uncultured_marine_eukaryote	EU446371	Uncultured	Marine	Mediterranean Sea	42.12
Environmental	EU446379_Uncultured_marine_eukaryote	EU446379	Uncultured	Marine	Mediterranean Sea	43.97
Environmental	EU446383_Uncultured_marine_eukaryote	EU446383	Uncultured	Marine	Mediterranean Sea	43.20
Environmental	EU446386_Uncultured_marine_eukaryote	EU446386	Uncultured	Marine	Mediterranean Sea	42.19
Environmental	EU446391_Uncultured_marine_eukaryote	EU446391	Uncultured	Marine	Mediterranean Sea	42.12
Environmental	EU446392_Uncultured_marine_eukaryote	EU446392	Uncultured	Marine	Mediterranean Sea	42.09
Environmental	EU446397_Uncultured_marine_eukaryote	EU446397	Uncultured	Marine	Mediterranean Sea	42.36
Environmental	EU446403_Uncultured_marine_eukaryote	EU446403	Uncultured	Marine	Mediterranean Sea	43.44
Environmental	EU446412_Uncultured_marine_eukaryote	EU446412	Uncultured	Marine	Mediterranean Sea	42.06
Environmental	EU446413_Uncultured_marine_eukaryote	EU446413	Uncultured	Marine	Mediterranean Sea	42.06
Environmental	EU446415_Uncultured_marine_eukaryote	EU446415	Uncultured	Marine	Mediterranean Sea	42.13
Environmental	FJ000241_Uncultured_eukaryote	FJ000241	Uncultured	Marine	Mediterranean Sea	43.51
Environmental	FJ153705_Uncultured_marine_eukaryote	FJ153705	Uncultured	Marine	Baltic Sea	42.34
Environmental	FJ153742_Uncultured_marine_eukaryote	FJ153742	Uncultured	Marine	Baltic Sea	42.04
Environmental	FJ592458_Uncultured_eukaryote	FJ592458	Uncultured	Soil	USA	48.77
Environmental	FJ810603_Uncultured_eukaryote	FJ810603	Uncultured	Freshwater	USA	45.30
Environmental	FN598312_Uncultured_Scuticocilia	FN598312	Uncultured	Marine	Pacific Ocean	40.95
Environmental	FN598313_Uncultured_Scuticocilia	FN598313	Uncultured	Marine	Pacific Ocean	41.34
Environmental	FN598351_Uncultured_Scuticocilia	FN598351	Uncultured	Marine	Pacific Ocean	41.04
Environmental	FN598371_Uncultured_Scuticocilia	FN598371	Uncultured	Marine	Pacific Ocean	41.55
Environmental	FN598378_Uncultured_Scuticocilia	FN598378	Uncultured	Marine	Pacific Ocean	41.55
Environmental	FN690015_Uncultured_alveolate	FN690015	Uncultured	Marine	Finland	44.28
Environmental	FN690016_Uncultured_alveolate	FN690016	Uncultured	Marine	Sweden	44.77
Environmental	FN690017_Uncultured_alveolate	FN690017	Uncultured	Marine	Finland	44.68
Environmental	GQ330629_Uncultured_Scuticocilia	GQ330629	Uncultured	Freshwater	Switzerland	46.18
Environmental	GQ330630_Uncultured_Scuticocilia	GQ330630	Uncultured	Freshwater	Switzerland	46.31
Environmental	GQ330631_Uncultured_Scuticocilia	GQ330631	Uncultured	Freshwater	Switzerland	46.83
Environmental	GU385667_Uncultured_marine_eukar	GU385667	Uncultured	Marine	USA	42.39
Environmental	GU479975_Uncultured_Scuticocilia	GU479975	Uncultured	Freshwater	Switzerland	47.01
Environmental	HM030717_Scuticociliatia_sp.	HM030717	Uncultured	Marine	China	43.82
Environmental	HM030718_Scuticociliatia_sp.	HM030718	Uncultured	Marine	China	43.31
Environmental	HM030719_Scuticociliatia_sp.	HM030719	Uncultured	Marine	China	43.58
Environmental	HM135062_Uncultured_alveolate	HM135062	Uncultured	Freshwater	China	42.20
Environmental	HM749904_Uncultured_marine_eukaryote	HM749904	Uncultured	Marine	Black Sea	43.27
Environmental	HM749906_Uncultured_marine_eukaryote	HM749906	Uncultured	Marine	Black Sea	42.42
Environmental	HM749908_Uncultured_marine_eukaryote	HM749908	Uncultured	Marine	Black Sea	45.80
Environmental	HM749909_Uncultured_marine_eukaryote	HM749909	Uncultured	Marine	Black Sea	42.68
Environmental	HM749910_Uncultured_marine_eukaryote	HM749910	Uncultured	Marine	Black Sea	42.47
Environmental	HM799981_Uncultured_alveolate	HM799981	Uncultured	Marine	Puerto Rico Trench	42.48
Environmental	HM800140_Uncultured_alveolate	HM800140	Uncultured	Marine	Puerto Rico Trench	46.68
Environmental	HQ219368_Uncultured_ciliate	HQ219368	Uncultured	Freshwater	France	44.58
Environmental	HQ219418_Uncultured_ciliate	HQ219418	Uncultured	Freshwater	France	44.72
Environmental	JF730754_Uncultured_eukaryote	JF730754	Uncultured	Freshwater	Canada	42.10
Environmental	JF730790_Uncultured_eukaryote	JF730790	Uncultured	Freshwater	Canada	42.18
Environmental	JN406267_Uncultured_ciliate	JN406267	Uncultured	Marine	Australia	44.18
Environmental	JN705530_Uncultured_microeukaryote	JN705530	Uncultured	Freshwater	USA	45.98
Environmental	JN705536_Uncultured_microeukaryote	JN705536	Uncultured	Freshwater	USA	46.72
Environmental	JQ692041_Uncultured_Scuticocilia	JQ692041	Uncultured	Marine	Argentina	43.98
Environmental	JQ692043_Uncultured_Scuticocilia	JQ692043	Uncultured	Marine	Argentina	44.10
Environmental	KC287215_Scuticociliatia	KC287215	Uncultured	Marine	Antarctica	42.21
Environmental	KF130445_Uncultured_eukaryote	KF130445	Uncultured	Marine	China	45.18
Environmental	KJ760283_Uncultured_eukaryote	KJ760283	Uncultured	Marine	USA	44.96
Environmental	KP404657_Uncultured_eukaryote	KP404657	Uncultured	Marine	China	42.62
Environmental	KP404747_Uncultured_eukaryote	KP404747	Uncultured	Marine	China	42.62
Environmental	KP404895_Uncultured_eukaryote	KP404895	Uncultured	Marine	China	42.62
Environmental	KT346283_Uncultured_ciliate	KT346283	Uncultured	Marine	USA	43.10
Environmental	KT346303_Uncultured_ciliate	KT346303	Uncultured	Marine	USA	43.67
Environmental	KT346313_Uncultured_ciliate	KT346313	Uncultured	Marine	USA	43.49
Environmental	KX465196_Uncultured_ciliate	KX465196	Uncultured	Freshwater	Kenya	44.90
Environmental	KX465197_Uncultured_ciliate	KX465197	Uncultured	Freshwater	Kenya	45.57
Environmental	KX465198_Uncultured_ciliate	KX465198	Uncultured	Freshwater	Kenya	45.58
Environmental	KX602069_Uncultured_Scuticocilia	KX602069	Uncultured	Marine	Argentina	43.98
Environmental	KX602071_Uncultured_Scuticocilia	KX602071	Uncultured	Marine	Argentina	44.10
Environmental	KX602081_Uncultured_Mamiellophyc	KX602081	Uncultured	Marine	Argentina	44.02
Environmental	KX602122_Uncultured_Ancistrum	KX602122	Uncultured	Marine	Argentina	44.04
Environmental	LC466977_Scuticociliatia_sp.	LC466977	Uncultured	Sludge (sewage treatment reactor)	Japan	44.97
Environmental	LC466978_Scuticociliatia_sp.	LC466978	Uncultured	Sludge (sewage treatment reactor)	Japan	44.85
Environmental	LC466979_Scuticociliatia_sp.	LC466979	Uncultured	Sludge (sewage treatment reactor)	Japan	44.91
Environmental	LC466980_Scuticociliatia_sp.	LC466980	Uncultured	Sludge (sewage treatment reactor)	Japan	44.91
Environmental	LC466981_Scuticociliatia_sp.	LC466981	Uncultured	Sludge (sewage treatment reactor)	Japan	44.85
Environmental	LC466982_Scuticociliatia_sp.	LC466982	Uncultured	Sludge (sewage treatment reactor)	Japan	44.97
Environmental	LC466983_Scuticociliatia_sp.	LC466983	Uncultured	Sludge (sewage treatment reactor)	Japan	44.91
Environmental	LC466984_Scuticociliatia_sp.	LC466984	Uncultured	Sludge (sewage treatment reactor)	Japan	45.03
Environmental	LN869943_Scuticociliatia_sp.	LN869943	Uncultured	Freshwater	Italy	43.06
Environmental	LN870017_Scuticociliatia_sp.	LN870017	Uncultured	Freshwater	Italy	44.46
Environmental	LN870030_Scuticociliatia_sp.	LN870030	Uncultured	Freshwater	Italy	43.94
Environmental	LR025746_uncultured_ciliate	LR025746	Uncultured	Freshwater	Switzerland	44.69
Environmental	LS999901_uncultured_ciliate	LS999901	Uncultured	Freshwater	Switzerland	41.89
Environmental	MK177608_Uncultured_Pseudocohnilembus	MK177608	Uncultured	Marine	South Korea	44.74
Environmental	MK177640_Uncultured_Uronema_sp.	MK177640	Uncultured	Marine	South Korea	43.33
Environmental	MK177641_Uncultured_eukaryote	MK177641	Uncultured	Marine	South Korea	43.99
Environmental	MK946018_Uncultured_soil_eukaryote	MK946018	Uncultured	Soil	USA	46.13

	Environmental	MK946032_Uncultured_soil_eukaryote	MK946032	Uncultured	Soil	USA	44.93
APOSTOMATIA		MN537438_Hyalophysa_bradburyae	MN537438	Hyalophysa bradburyae	Parasitic (freshwater host)	USA	42.14
APOSTOMATIA		EU503538_Hyalophysa_lwoffi	EU503538	Hyalophysa lwoffi	Parasitic (freshwater host)	USA	42.24
APOSTOMATIA		MH200618_Metacollinia_luciensis	MH200618	Metacollinia luciensis	Parasitic (marine host)	Canada	43.91
APOSTOMATIA		HQ591477_Pseudocollinia_beringen	HQ591477	Pseudocollinia beringensis	Parasitic (marine host)	Pacific Ocean	40.86
APOSTOMATIA		HQ591470_Pseudocollinia_brintoni	HQ591470	Pseudocollinia brintoni	Parasitic (marine host)	Pacific Ocean	41.09
APOSTOMATIA		HQ591473_Pseudocollinia_oregonen	HQ591473	Pseudocollinia oregonensis	Parasitic (marine host)	Pacific Ocean	40.72
APOSTOMATIA		HQ591485_Pseudocollinia_similis	HQ591485	Pseudocollinia similis	Parasitic (marine host)	Pacific Ocean	40.80
ASTOMATIA		HQ446281_Almophrya_bivacuolata	HQ446281	Almophrya bivacuolata	Parasitic (terrestrial host)	Cameroon	44.30
ASTOMATIA		MN121061_Anoplophrya_lumbrici	MN121061	Anoplophrya lumbrici	Parasitic (terrestrial host)	Slovakia	43.65
ASTOMATIA		MN121065_Anoplophrya_vulgaris	MN121065	Anoplophrya vulgaris	Parasitic (terrestrial host)	Slovakia	44.40
ASTOMATIA		MH035978_Haptophrya_planariarum	MH035978	Haptophrya planariarum	Parasitic (freshwater host)	Slovakia	42.61
ASTOMATIA		MK454737_Haptophrya_planariarum	MK454737	Haptophrya planariarum	Parasitic (freshwater host)	Slovakia	43.03
ASTOMATIA		MK454738_Haptophrya_planariarum	MK454738	Haptophrya planariarum	Parasitic (freshwater host)	Slovakia	42.67
ASTOMATIA		MK454740_Haptophrya_planariarum	MK454740	Haptophrya planariarum	Parasitic (freshwater host)	Slovakia	42.67
ASTOMATIA		MK454741_Haptophrya_planariarum	MK454741	Haptophrya planariarum	Parasitic (freshwater host)	Slovakia	42.55
ASTOMATIA		MK454742_Haptophrya_planariarum	MK454742	Haptophrya planariarum	Parasitic (freshwater host)	Slovakia	42.55
ASTOMATIA		MK454743_Haptophrya_planariarum	MK454743	Haptophrya planariarum	Parasitic (freshwater host)	Slovakia	42.55
ASTOMATIA		MK454744_Haptophrya_planariarum	MK454744	Haptophrya planariarum	Parasitic (freshwater host)	Slovakia	42.55
ASTOMATIA		MK454746_Haptophrya_planariarum	MK454746	Haptophrya planariarum	Parasitic (freshwater host)	Slovakia	42.55
ASTOMATIA		MK454747_Haptophrya_planariarum	MK454747	Haptophrya planariarum	Parasitic (freshwater host)	Slovakia	42.63
ASTOMATIA		HQ446277_Metaracoelophrya_sp.	HQ446277	Metaracoelophrya sp.	Parasitic (terrestrial host)	Cameroon	43.58
ASTOMATIA		HQ446282_Metaracoelophrya_sp.	HQ446282	Metaracoelophrya sp.	Parasitic (terrestrial host)	Cameroon	43.91
ASTOMATIA		MN121068_Metaradiophrya_lumbrici	MN121068	Metaradiophrya lumbrici	Parasitic (terrestrial host)	Slovakia	44.44
ASTOMATIA		MN121069_Metaradiophrya_lumbrici	MN121069	Metaradiophrya lumbrici	Parasitic (terrestrial host)	Slovakia	44.44
ASTOMATIA		MN121070_Metaradiophrya_lumbrici	MN121070	Metaradiophrya lumbrici	Parasitic (terrestrial host)	Slovakia	44.44
ASTOMATIA		MN121071_Metaradiophrya_lumbrici	MN121071	Metaradiophrya lumbrici	Parasitic (terrestrial host)	Slovakia	44.50
ASTOMATIA		MN121072_Metaradiophrya_lumbrici	MN121072	Metaradiophrya lumbrici	Parasitic (terrestrial host)	Slovakia	44.44
ASTOMATIA		MN121073_Metaradiophrya_lumbrici	MN121073	Metaradiophrya lumbrici	Parasitic (terrestrial host)	Slovakia	44.50
ASTOMATIA		MN121074_Metaradiophrya_lumbrici	MN121074	Metaradiophrya lumbrici	Parasitic (terrestrial host)	Slovakia	44.44
ASTOMATIA		MN121075_Metaradiophrya_lumbrici	MN121075	Metaradiophrya lumbrici	Parasitic (terrestrial host)	Slovakia	44.44
ASTOMATIA		HQ446279_Metaradiophrya_sp.	HQ446279	Metaradiophrya sp.	Parasitic (terrestrial host)	Cameroon	44.30
ASTOMATIA		MN121076_Metaradiophrya_varians	MN121076	Metaradiophrya varians	Parasitic (terrestrial host)	Slovakia	43.92
ASTOMATIA		MN121077_Metaradiophrya_varians	MN121077	Metaradiophrya varians	Parasitic (terrestrial host)	Slovakia	43.92
ASTOMATIA		MN121078_Metaradiophrya_varians	MN121078	Metaradiophrya varians	Parasitic (terrestrial host)	Slovakia	43.92
ASTOMATIA		MN121079_Metaradiophrya_varians	MN121079	Metaradiophrya varians	Parasitic (terrestrial host)	Slovakia	43.92
ASTOMATIA		HQ446276_Njinella_prolifera	HQ446276	Njinella prolifera	Parasitic (terrestrial host)	Cameroon	45.43
ASTOMATIA		HQ446275_Paraclausilocola_constricta	HQ446275	Paraclausilocola constricta	Parasitic (terrestrial host)	Cameroon	44.58
ASTOMATIA		HQ446274_Paraclausilocola_elongata	HQ446274	Paraclausilocola elongata	Parasitic (terrestrial host)	Cameroon	44.58
ASTOMATIA		MN121063_Subanoplophrya_nodulata	MN121063	Subanoplophrya nodulata	Parasitic (terrestrial host)	Slovakia	43.67
COLPODEA		EU039895_Colpoda_lucida	EU039895	Colpoda lucida	Freshwater	USA	44.48
COLPODEA		EU039896_Colpoda_magna	EU039896	Colpoda magna	Freshwater	USA	44.29
COLPODEA		JF747217_Maryna_umbrellata	JF747217	Maryna umbrellata	Freshwater	Austria	44.59
COLPODEA		EU039905_Platyophrya_bromelicola	EU039905	Platyophrya bromelicola	Freshwater	USA	45.46
HYMENOSTOMATIA		U17354_Ichthyophthirius_multifiliis	U17354	Ichthyophthirius multifiliis	Parasitic (freshwater host)	Canada	43.71
HYMENOSTOMATIA		U17355_Ophryoglena_catenula	U17355	Ophryoglena catenula	Parasitic (freshwater host)	Canada	43.68
HYMENOSTOMATIA		EF070253_Tetrahymena_paravorax	EF070253	Tetrahymena paravorax	Freshwater	Canada	42.95
HYMENOSTOMATIA		M26358_Tetrahymena_pigmentosa	M26358	Tetrahymena pigmentosa	Freshwater	USA	42.64
HYMENOSTOMATIA		M98021_Tetrahymena_pyriformis	M98021	Tetrahymena pyriformis	Freshwater	USA	42.78
PENICULIA		FJ876953_Frontonia_magna	FJ876953	Frontonia magna	Brackish water	China	44.65
PENICULIA		KU729877_Paramecium_biaurelia	KU729877	Paramecium biaurelia	Freshwater	Germany	43.99
PENICULIA		KX302699_Paramecium_caudatum	KX302699	Paramecium caudatum	Freshwater	China	44.97
PENICULIA		HE662760_Paramecium_jenningsi	HE662760	Paramecium jenningsi	Freshwater	Pakistan	43.96
PENICULIA		AF100315_Paramecium_primaurelia	AF100315	Paramecium primaurelia	Freshwater	Canada	44.62
PENICULIA		X03772_Paramecium_tetraurelia	X03772	Paramecium tetraurelia	Freshwater	USA	44.19
PERITRICHIA		HM852991_Carchesium_polypinum	HM852991	Carchesium polypinum	Freshwater	Canada	42.35
PERITRICHIA		DQ190462_Epicarchesium_abrae	DQ190462	Epicarchesium abrae	Marine	China	42.61
PERITRICHIA		DQ662847_Pseudovorticella_paracratera	DQ662847	Pseudovorticella paracratera	Marine	China	42.93
PERITRICHIA		AY788099_Trichodina_heterodentata	AY788099	Trichodina heterodentata	Freshwater	China	50.64
PERITRICHIA		AY102176_Trichodinella_myakkae	AY102176	Trichodinella myakkae	Freshwater	China	50.54
PERITRICHIA		DQ662849_Vorticella_campanula	DQ662849	Vorticella campanula	Freshwater	China	42.65
PERITRICHIA		KM222118_Zoothamnium_hentscheli	KM222118	Zoothamnium hentscheli	Marine	China	42.72
UROCENTRIA		EF114301_Urocentrum_sp1	EF114301	Urocentrum sp1	Freshwater	Germany	43.01
UROCENTRIA		EF114302_Urocentrum_sp2	EF114302	Urocentrum sp2	Freshwater	Germany	42.83
UROCENTRIA		AF255357_Urocentrum_turbo	AF255357	Urocentrum turbo	Freshwater	Canada	42.77
UROCENTRIA		EF114299_Urocentrum_turbo	EF114299	Urocentrum turbo	Freshwater	Germany	42.89
UROCENTRIA		EF114300_Urocentrum_turbo	EF114300	Urocentrum turbo	Freshwater	Germany	42.57
SCUTICOCILIATIA	Anaerocyclidiidae	Z29517_Cyclidium_porcatum	Z29517	Anaerocyclidium porcatum	Freshwater	UK	43.47
SCUTICOCILIATIA	Anaerocyclidiidae	LC497866_Cyclidium_sp.	LC497866	Anaerocyclidium sp.	Sludge (sewage treatment reactor)	Japan	44.47
SCUTICOCILIATIA	Cinetochilididae	FJ870103_Cinetochilum_ovale	FJ870103	Cinetochilides ovalis	Marine	China	44.61

Supplementary Table S4 Statistical data of sequence identity within Scuticociliatia and its three orders

Overall pairwise sequence identity		Groups			
**Genes**		**Scuticociliatia**	**Loxocephalida**	**Pleuronematida**	**Philasterida**
SSU rRNA gene	Range	76.0%–100%	85.9%–100%	79.2%–100%	83.7%–100%
	Average	87.7%	90.1%	87.7%	92.5%
	Alignment length	1590	1650	1695	1512
	Number of sequences[a]	239	24	107	108
LSU rRNA gene	Range	41.7%–100%	78.9%–98.3%	39.6%–100%	81.8%–100%
	Average	73.6%	85.8%	72.4%	87.1%
	Alignment length	1043	1735	1080	1198
	Number of sequences	55	7	16	32
ITS1-5.8S-ITS2 region	Range	37.9%–100%	46.0%–70.0%	47.6%–100%	41.5%–100%
	Average	61.0%	55.2%	57.6%	72.2%
	Alignment length	668	564	610	609
	Number of sequences	73	7	22	44
COI gene	Range	59.2%–100%	69.2%–81.2%	61.5%–100%	74.8%–100%
	Average	72.1%	73.5%	71.2%	82.4%
	Alignment length	678	770	742	600
	Number of sequences	90	5	42	43

[a]Six SSU rRNA gene sequences of Scuticociliatia that significantly shortened were deleted from the 245 Scuticociliatia sequences when calculating identity

Supplementary Table S5 Summary of topological characteristics of putative ITS2 secondary structures in the present work

	Topological characteristics	Min	Max	Mean	M	SD	SE	CV	n
Philasterida	Total length	167	190	173.4	172	4.90	0.76	2.83	42
	G + C content	30.86%	45.71%	36.16%	36.05%	0.03	0.00	8.05	42
	Length of helix II	22	32	26.83	26	2.41	0.37	8.98	42
	Length of helix III	89	113	93.76	93	3.26	0.50	3.48	42
	Length of helix IV	NA	NA	NA	NA	NA	NA	NA	NA
	Unpaired bases in central loop	33	50	37.79	37	3.03	0.47	8.03	42
	Unpaired bases in terminal loop of helix II	3	10	4.52	4	1.40	0.22	30.97	42
	Unpaired bases in terminal loop of helix III	4	10	5.29	5	0.94	0.15	17.87	42
	Unpaired bases in terminal loop of helix IV	NA	NA	NA	NA	NA	NA	NA	NA
	Unpaired bases in bulge(s) of helix II	4	6	4.07	4	0.34	0.05	8.39	42
	Unpaired bases in bulge(s) of helix III	14	23	16.81	16	1.58	0.24	9.41	42
	Unpaired bases in bulge(s) of helix IV	NA	NA	NA	NA	NA	NA	NA	NA
	Bulges in helix III, no.	4	8	5.98	6	0.60	0.09	10.11	42
	GU pairings, no.	2	7	4.14	4	1.32	0.20	31.80	42
	ΔG (37°C, kcal/mol)	-69.41	-51.6	-59.45	-58.3	3.68	0.57	-6.19	42
	Topological characteristics	Min	Max	Mean	M	SD	SE	CV	n
Pleuronematida	Total length	157	242	187.8	175	24.81	6.40	13.21	15
	G + C content	30.46%	47.77%	40.85%	41.63%	0.05	0.01	11.14	15
	Length of helix II	24	63	36.93	34	11.11	2.87	30.09	15
	Length of helix III	76	128	94.73	93	13.87	3.58	14.64	15
	Length of helix IV	NA	NA	NA	NA	NA	NA	NA	NA
	Unpaired bases in central loop	24	53	39.40	39	6.39	1.65	16.22	15
	Unpaired bases in terminal loop of helix II	3	6	4.80	5	1.15	0.30	23.88	15
	Unpaired bases in terminal loop of helix III	3	5	4.00	4	0.38	0.10	9.45	15
	Unpaired bases in terminal loop of helix IV	NA	NA	NA	NA	NA	NA	NA	NA
	Unpaired bases in bulge(s) of helix II	4	17	6.67	4	4.15	1.07	62.28	15
	Unpaired bases in bulge(s) of helix III	16	42	22.20	20	7.54	1.95	33.97	15
	Unpaired bases in bulge(s) of helix IV	NA	NA	NA	NA	NA	NA	NA	NA
	Bulges in helix III, no.	4	9	6.00	6	1.56	0.40	25.97	15
	GU pairings, no.	4	10	7.00	8	1.93	0.50	27.53	15
	ΔG (37°C, kcal/mol)	-92.18	-47.49	-67.84	-66.24	10.95	2.83	-16.14	15
	Topological characteristics	Min	Max	Mean	M	SD	SE	CV	n
Loxocephalida	Total length	130	215	170.0	169	25.85	9.77	15.20	7
	G + C content	31.25%	50.28%	40.73%	39.75%	0.07	0.02	16.20	7
	Length of helix II	20	52	30.57	27	10.77	4.07	35.22	7
	Length of helix III	61	94	82.71	86	10.55	3.99	12.75	7
	Length of helix IV	11	20	14.60	14	3.29	1.47	22.51	5
	Unpaired bases in central loop	21	49	34.71	32	9.91	3.75	28.55	7
	Unpaired bases in terminal loop of helix II	3	4	3.86	4	0.38	0.14	9.80	7
	Unpaired bases in terminal loop of helix III	3	5	4.14	4	0.69	0.26	16.66	7
	Unpaired bases in terminal loop of helix IV	3	6	4.20	4	1.10	0.49	26.08	5
	Unpaired bases in bulge(s) of helix II	4	10	5.00	4	2.24	0.85	44.72	7
	Unpaired bases in bulge(s) of helix III	9	26	18.57	19	5.91	2.23	31.83	7
	Unpaired bases in bulge(s) of helix IV	NA	NA	NA	NA	NA	NA	NA	NA
	Bulges in helix III, no.	2	6	4.71	5	1.50	0.57	31.73	7
	GU pairings, no.	3	11	5.71	4	2.81	1.06	49.20	7
	ΔG (37°C, kcal/mol)	-87.75	-46.18	-60.44	-52.76	15.54	5.87	-25.72	7

CV, coefficient of variation in %; *M*, median; *Max*, maximum; *Mean*, arithmetic mean; *Min*, minimum; *n*, number of specimens observed; *NA*, not applicable or data not available; *SD*, standard deviation; *SE*, standard error of arithmetic mean; ΔG, Gibbs free energy

Supplementary Table S6 Topological characteristics of ITS2 secondary structures in the present work

**Order**	**Species**	**GenBank accession number**	**Total length**	**G + C content**	**Length of helix** I	II	III	IV	**Unpaired bases, no.** Central loop	Terminal loop of helix II	Terminal loop of helix III	Terminal loop of helix IV	Bulge(s) of helix II	Bulge(s) of helix III	Bulge(s) of helix IV	**Other information** Bulges in helix III, no.	GU pairings, no.	ΔG (37° C, kcal/mol)
Philasterida	*Uronema marinum*	GQ259751	171	35.09%	NA	26	93	NA	37	4	5	NA	4	16	NA	6	3	-58.30
	Uronema marinum	GQ259752	171	35.09%	NA	26	93	NA	37	4	5	NA	4	16	NA	6	3	-58.30
	Uronema marinum	GQ259753	171	35.09%	NA	26	93	NA	37	4	5	NA	4	16	NA	6	3	-58.30
	Uronema marinum	GQ259754	171	35.09%	NA	26	93	NA	37	4	5	NA	4	16	NA	6	3	-58.30
	Uronema marinum	GQ259755	171	36.84%	NA	26	93	NA	37	4	5	NA	4	16	NA	6	4	-58.00
	Uronema marinum	MF992244	171	36.26%	NA	26	93	NA	37	4	5	NA	4	16	NA	6	4	-57.90
	Uronema marinum	MF992245	171	36.26%	NA	26	93	NA	37	4	5	NA	4	16	NA	6	4	-57.90
	Uronema marinum	MF992246	171	36.84%	NA	26	93	NA	37	4	5	NA	4	16	NA	6	4	-58.00
	Uronema marinum	MF992247	171	36.84%	NA	26	93	NA	37	4	5	NA	4	16	NA	6	4	-58.00
	Uronema marinum	JN885102	171	35.67%	NA	26	93	NA	37	4	5	NA	4	16	NA	6	4	-57.90
	Uronema apomarinum	MN727051	171	35.09%	NA	26	93	NA	41	4	5	NA	4	16	NA	6	4	-57.39
	Uronema orientalis	PP852880	172	36.63%	NA	26	93	NA	38	4	5	NA	4	16	NA	6	4	-57.97
	Uronema elegans	AY513760	170	31.76%	NA	24	93	NA	38	4	5	NA	4	18	NA	6	3	-55.47
	Uronema heteromarinum	JN885101	173	35.84%	NA	29	93	NA	36	5	5	NA	4	14	NA	7	5	-59.33
	Uronema nigricans	PP852883	175	30.86%	NA	30	93	NA	37	4	5	NA	4	20	NA	7	2	-53.10
	Uronema sp.	JN885107	170	34.71%	NA	26	93	NA	36	4	5	NA	4	16	NA	6	3	-58.33
	Uronemita sinensis	JN885108	172	32.56%	NA	27	93	NA	37	7	5	NA	4	20	NA	7	2	-51.60
	Uronemita filificum	PP852882	168	32.14%	NA	24	94	NA	35	4	5	NA	4	17	NA	6	3	-55.86
	Uronemita parafilificum	JN885103	174	31.61%	NA	27	95	NA	37	3	5	NA	4	18	NA	6	3	-56.30
	Homalogastra setosa	EF158844	183	37.70%	NA	32	95	NA	41	4	6	NA	4	17	NA	6	6	-67.69
	Homalogastra parasetosa	MN727050	188	31.38%	NA	26	113	NA	34	4	6	NA	4	23	NA	8	4	-59.29
	Parauronema virginianum	JN885109	171	35.67%	NA	26	93	NA	37	4	5	NA	4	16	NA	6	4	-57.90
	Parauronema cf. *virginianum*	JN885106	171	35.67%	NA	26	93	NA	37	4	5	NA	4	16	NA	6	3	-58.40
	Parauronema longum	AY513759	173	36.42%	NA	22	94	NA	42	4	5	NA	4	17	NA	6	3	-56.56
	Parauronema longum	JN885096	173	36.42%	NA	22	94	NA	42	4	5	NA	4	17	NA	6	3	-56.56
	Miamiensis avidus	JN885095	176	38.07%	NA	30	89	NA	42	10	5	NA	4	16	NA	6	5	-61.26
	Glauconema trihymene	HM099917	173	35.26%	NA	28	94	NA	35	4	5	NA	4	17	NA	6	6	-58.26
	Cohnilembus verminus	JN885093	175	45.71%	NA	26	93	NA	41	4	4	NA	6	19	NA	6	5	-61.99
	Pseudocohnilembus hargisi	AY513753	176	40.34%	NA	26	93	NA	38	4	5	NA	4	16	NA	6	4	-69.41
	Pseudocohnilembus persalinus	EU262622	167	43.71%	NA	26	91	NA	35	4	5	NA	4	14	NA	4	7	-65.46
	Philaster sinensis	PP852879	170	37.06%	NA	26	94	NA	35	4	5	NA	4	17	NA	6	3	-61.36
	Philaster apodigitiformis	JN885097	170	37.06%	NA	26	94	NA	35	4	5	NA	4	17	NA	6	3	-61.36
	Philasterides armatalis	JN885098	167	35.93%	NA	22	95	NA	35	4	5	NA	4	18	NA	6	3	-57.26
	Porpostoma notata	JN885099	172	36.05%	NA	26	94	NA	37	4	5	NA	4	17	NA	6	6	-59.00
	Metanophrys similis	AY513757	172	36.05%	NA	29	92	NA	36	5	10	NA	4	16	NA	5	5	-57.53
	Metanophrys sinensis	JN885092	190	33.68%	NA	30	95	NA	50	5	5	NA	5	18	NA	6	4	-59.57
	Metanophrys orientalis	JN885110	179	35.75%	NA	29	94	NA	41	5	6	NA	4	18	NA	5	7	-60.99
	Mesanophrys carcini	JN885094	172	38.95%	NA	29	95	NA	33	7	5	NA	4	16	NA	6	7	-62.22
	Mesanophrys carcini	JN885104	172	38.37%	NA	29	93	NA	35	3	5	NA	4	16	NA	6	5	-64.66
	Paranophrys magna	AY513755	180	36.11%	NA	32	92	NA	41	8	6	NA	4	16	NA	5	5	-63.39
	Paranophrys magna	JN885105	180	36.11%	NA	32	92	NA	41	8	8	NA	4	16	NA	5	5	-62.69
	Citrithrix smalli	PP852881	178	41.01%	NA	28	96	NA	39	4	6	NA	4	18	NA	6	6	-68.04
Pleuronematida	*Pleuronema coronatum*	AY513754	166	46.99%	NA	30	84	NA	42	6	4	NA	4	16	NA	4	10	-65.36
	Pleuronema parasmalli	PP852884	157	47.77%	NA	24	80	NA	39	4	4	NA	4	20	NA	6	4	-56.24
	Pleuronema setigerum	JX310006	158	34.18%	NA	27	76	NA	41	3	4	NA	4	16	NA	4	4	-47.49
	Pleuronema setigerum	JX310011	175	40.00%	NA	31	92	NA	38	5	4	NA	4	16	NA	4	8	-68.07
	Pleuronema cf. *setigerum*	JX310003	173	38.73%	NA	30	90	NA	39	4	4	NA	4	20	NA	5	8	-66.24
	Pleuronema sp.	JX310008	174	30.46%	NA	37	78	NA	45	4	4	NA	5	18	NA	4	5	-59.48
	Schizocalyptra sinica	JX310010	168	42.86%	12	25	93	NA	24	5	3	NA	4	18	NA	7	5	-64.68
	Histiobalantium minor	JX310005	174	39.08%	NA	30	96	NA	34	4	4	NA	6	20	NA	7	8	-57.69
	Hippocomos salinus	JX310023	203	44.33%	NA	34	115	NA	40	4	4	NA	4	35	NA	7	8	-70.71
	Wilbertia typica	JX310031	197	40.10%	NA	46	96	NA	39	6	5	NA	14	19	NA	6	7	-70.84
	Cyclidium varibonneti	KF256834	192	41.67%	NA	42	92	NA	42	6	4	NA	4	18	NA	6	10	-78.76
	Protocyclidium citrullus	KF256832	215	37.67%	NA	40	99	25	35	6	4	5	8	23	0	8	6	-81.36
	Cristigera media	KF256827	214	43.46%	NA	40	107	NA	53	3	4	NA	7	29	NA	9	6	-65.90
	Cristigera pleuronemoid	KF256828	242	43.80%	NA	55	128	NA	45	6	4	NA	11	42	NA	6	8	-92.18
	Ancistrum crassum	KF256831	209	41.63%	NA	63	95	NA	35	6	4	NA	17	23	NA	7	8	-72.56
Loxocephalida	*Pseudoplatynematum denticulatum*	JX310004	176	31.25%	NA	28	86	20	31	4	3	4	4	19	0	4	7	-57.19
	Sathrophilus planus	JX310007	158	38.61%	NA	26	83	14	21	4	4	6	4	23	0	6	3	-49.92
	Sathrophilus holtae	JX310029	161	39.75%	NA	24	80	11	32	4	4	3	4	22	0	6	4	-52.76
	Paratetrahymena wassi	JX310009	181	50.28%	NA	37	86	14	27	4	5	4	5	13	0	5	11	-87.75
	Cardiostomatella vermiformis	EU262621	215	47.44%	NA	52	94	14	38	4	4	4	10	26	0	6	7	-76.57
	Dexitrichides pangi	AY513758	169	35.50%	NA	27	89	NA	49	3	5	NA	4	18	NA	4	4	-52.69
	Cinetochilum ovale	JX310025	130	42.31%	NA	20	61	NA	45	4	4	NA	4	9	NA	2	4	-46.18

NA , not applicable; *no.* , number

Supplementary Table S7 Sequence comparison of ITS2 region that with similar secondary structures

ITS2 region sequences	**1**	**2**	**3**	**4**	**5**	**6**	**7**	**8**	**9**	**10**	**11**	**12**
**1** JN885109 *Parauronema virginianum*	ID	99.0%	99.0%	99.5%	99.5%	99.0%	99.0%	96.0%	96.0%	96.0%	95.5%	99.0%
**2** JN885106 *Parauronema* cf. *virginianum*	2	ID	99.0%	99.5%	99.5%	99.0%	99.0%	96.0%	96.0%	96.0%	95.5%	99.0%
**3** JN885102 *Uronema marinum*	2	2	ID	99.5%	99.5%	99.0%	99.0%	97.0%	97.0%	97.0%	96.5%	99.0%
**4** MF992244 *Uronema marinum*	1	1	1	ID	100%	99.5%	99.5%	96.5%	96.5%	96.5%	96.0%	99.5%
**5** MF992245 *Uronema marinum*	1	1	1	0	ID	99.5%	99.5%	96.5%	96.5%	96.5%	96.0%	99.5%
**6** MF992246 *Uronema marinum*	2	2	2	1	1	ID	100%	97.0%	97.0%	97.0%	96.5%	100%
**7** MF992247 *Uronema marinum*	2	2	2	1	1	0	ID	97.0%	97.0%	97.0%	96.5%	100%
**8** GQ259751 *Uronema marinum*	8	8	6	7	7	6	6	ID	100%	100%	99.5%	97.0%
**9** GQ259752 *Uronema marinum*	8	8	6	7	7	6	6	0	ID	100%	99.5%	97.0%
**10** GQ259753 *Uronema marinum*	8	8	6	7	7	6	6	0	0	ID	99.5%	97.0%
**11** GQ259754 *Uronema marinum*	9	9	7	8	8	7	7	1	1	1	ID	96.5%
**12** GQ259755 *Uronema marinum*	2	2	2	1	1	0	0	6	6	6	7	ID

Supplementary Table S8 Comparison of *Uronema* and *Parauronema* SSU rRNA gene sequence within the same clade

SSU rRNA gene sequences	**1**	**2**	**3**	**4**	**5**	**6**	**7**	**8**	**9**	**10**	**11**	**12**	**13**	**14**	**15**	**16**
**1** JN885087 *Parauronema virginianum*	ID	99.3%	99.1%	99.2%	99.2%	99.3%	99.5%	99.4%	99.4%	99.4%	98.2%	98.2%	97.9%	98.1%	98.1%	99.4%
**2** AY392128 *Parauronema virginianum*	10	ID	99.2%	99.4%	99.5%	99.6%	99.8%	99.7%	99.7%	99.7%	98.0%	98.2%	97.8%	98.0%	98.0%	99.8%
**3** JN885082 *Parauronema* cf. *virginianum*	13	11	ID	99.2%	99.2%	99.4%	99.4%	99.4%	99.4%	99.4%	98.0%	98.1%	97.7%	97.9%	97.9%	99.4%
**4** FJ595488 *Parauronema* cf. *virginianum*	12	8	11	ID	99.4%	99.6%	99.6%	99.6%	99.6%	99.6%	97.9%	98.0%	97.6%	97.8%	97.8%	99.6%
**5** MF992243 *Uronema marinum*	11	7	12	9	ID	99.5%	99.7%	99.8%	99.8%	99.8%	97.8%	98.0%	97.6%	97.8%	97.8%	99.6%
**6** GQ465466 *Uronema marinum*	10	6	9	6	7	ID	99.8%	99.7%	99.7%	99.7%	98.0%	98.2%	97.8%	98.0%	98.0%	99.7%
**7** GQ259749 *Uronema marinum*	7	3	8	5	4	3	ID	99.9%	99.9%	99.9%	98.1%	98.2%	97.8%	98.0%	98.0%	99.9%
**8** MF992242 *Uronema marinum*	8	4	9	6	3	4	1	ID	100%	100%	98.0%	98.2%	97.8%	98.0%	98.0%	99.8%
**9** MF992241 *Uronema marinum*	8	4	9	6	3	4	1	0	ID	100%	98.0%	98.2%	97.8%	98.0%	98.0%	99.8%
**10** MF992240 *Uronema marinum*	8	4	9	6	3	4	1	0	0	ID	98.0%	98.2%	97.8%	98.0%	98.0%	99.8%
**11** AY551905 *Uronema marinum*	27	29	30	31	32	29	28	29	29	29	ID	99.8%	99.6%	99.8%	99.8%	98.2%
**12** DQ867072 *Uronema marinum*	27	27	28	29	30	27	26	27	27	27	2	ID	99.6%	99.8%	99.8%	98.3%
**13** Z22881 *Uronema marinum*	31	33	34	35	36	33	32	33	33	33	6	6	ID	99.5%	99.5%	97.9%
**14** MF418591 *Uronema marinum*	28	30	31	32	33	30	29	30	30	30	3	3	7	ID	99.7%	98.1%
**15** GQ259744 *Uronema marinum*	28	30	31	32	33	30	29	30	30	30	3	3	7	4	ID	98.1%
**16** KY569295 *Uronema* sp.	8	2	9	6	5	4	1	2	2	2	27	25	31	28	28	ID
